# Supplementary material for: Differential IRF8 Transcription Factor Requirement Defines Two Pathways of Dendritic Cell Development in Humans
Source: Immunity. 2020 Aug 18;53(2):353–370.e8. doi: 10.1016/j.immuni.2020.07.003 (PMC7447982; doi:10.1016/j.immuni.2020.07.003)
Supplement: Document S1. Tables S1–S4 and Figures S1–S7 [file mmc1.pdf]

Immunity, Volume *53*

Supplemental Information

Differential IRF8 Transcription Factor Requirement Defines Two Pathways of Dendritic Cell Development in Humans

Urszula Cytlak, Anastasia Resteu, Sarah Pagan, Kile Green, Paul Milne, Sheetal Maisuria, David McDonald, Gillian Hulme, Andrew Filby, Benjamin Carpenter, Rachel Queen, Sophie Hambleton, Rosie Hague, Hana Lango Allen, James E.D. Thaventhiran, Gina Doody, Matthew Collin, and Venetia Bigley

Table S1. DC2 and DC3 marker genes identified by Villani et al., (related to Figure S1)

Transcripts identified as DC2 and DC3 marker genes by Single Cell RNA-Seq in Villani et al., and present in our BM Single Cell RNA-Seq dataset, used to cluster BM DCs in Figure S1J,K

DC2 Genes	DC3 Genes	
SLC2A3	MGST1	IL1RN
FCGR2B	MTMR11	ASGR1
PTGS1	VCAN	NLRP12
CD33	SLC2A3	ADAM15
AREG	RAB27A	S100A8
CLEC4A	FCN1	MPP7
CCR6	LAT2	HNMT
CD2	LYZ	NR4A2
MBOAT7	RETN	PID1
CLEC10A	IL27RA	CD1D
ENTPD1	RAB3D	LMNA
ADAM8	BST1	ITGA5
NR4A2	HBEGF	NLRP3
PID1	CSF3R	S100A9
CLIC2	PLBD1	S100A12
CACNA2D3	CSTA	MNDA
ETS2	NFE2	NOD2
CD1D	F13A1	RNASE2
CD1C	TREM1	PTAFR
CD1E	EREG	CD14
ITGA5	IL1B	FPR1
PEA15	IL13RA1	CD163
NOD2	CLEC10A	FCER1A
PTAFR	MICAL2	TMEM173
PER1	ANXA1	CES1
FCER1A	CD36	NAIP
CLEC17A	AOAH	

Table S2. CyTOF Panel (Related to Figures 1 and 5)

Antigens included in the CyTOF panel and the antibody metal conjugate. For antibody details see **Key Resources Table**

Antigen	Metal	Surface/intracellular
CD14	113In	S
CD370/CLECL9A	141Pr	S
AXL	142Md	S
CD123	143Nd	S
CD11b	144Nd	S
CD116	145Nd	S
SIGLEC6	146Nd	S
CD303	147Sm	S
SIRPA	148Nd	S
IRF8	149Sm	IC
FCER1	150Nd	S
CD2	141Eu	S
IRF4	152Sm	IC
CD45RA	153Eu	S
CD38	154Sm	S
CD36	155Gd	S
CD10	156Gd	S
Lineage-FITC (CD3, 19, 20, 56)	157Gd (secondary)	S
CD33	158Gd	S
CD11c	159Tb	S
ID2	160Gd	S
CD90	161Dy	S
SLAN-PE	162Dy (secondary)	S
BTLA	163Dy	S
CD15	164Dy	S
CD141	165Ho	S
CD34	166Er	S
CD115	167Er	S
CD100	168Er	S
CD304	169Tm	S
CD135	170Er	S
CD88	171Yb	S
CD117	172Yb	S
HLA=DR	173Yb	S
CD1c	174Yb	S
CD5	175Lu	S
CX3CR1-APC	176Yb	S
CD16	209Bi	S
CD45 (PBMC)	115In	S
CD45 (BM)	89Y	S
Cisplatin	195Pt	
DNA	191Ir	

Table S3. Single Cell analysis parameters (related to Figures 1, 3, 4, S3, S4)

Dataset		BM CD34+ Progenitors	BM CD34med pre-DC and DC	PB pre-DC
Figures		Figures 3E-J; S3D-H	1H, S1J-L, S4E-K, S4H-J	S4K-Q
Raw Data Analysis and QC CELLS	Total cells sequenced	399	260	184
	ERCC % threshold (adjusted for concentration of ERCC spike added)	30%	70%	25%
	Mitochondrial % threshold	25%	15%	30%
	Total features threshold	>2500	>1700	>3000, <5000
	Total counts threshold	>50000	>50000	>25000
	Number of cells retained after cell filtering	262	244	116
Raw Data Analysis and QC GENES	Number of genes expressed at > 2 counts in > 2 cells	18791	17850	14458
	Number of genes retained after removing cell cycle genes	18181	17237	13920
	Number of protein-coding genes retained	12406	12137	10346
Whole dataset (all cells, all genes)	**Hierarchical Clustering**	**Figure 3F, S3H**	**Figure F, S4J**	**Figure S4N**
	Cells in analysis	262	244	116
	Genes retained for analysis by SC3	6,846	6,838	7,265
	Clustering solutions explored	2:10	2:15	2:15
	Clustering solution	10	15	8
	Average Silhouette width	0.45	0.58	0.27
	Highest cluster stability index	0.63	0.7	0.4
	Marker gene p value threshold	0.01	0.01	0.05
	AUROC threshold	0.75	0.85	0.7
	**tSNE**	**Figure 3E-G**	**Figure 4E-G, S4I**	**Figure S4O-P**
	Cells in analysis	262	244	116
	Genes in analysis	6,846	6,838	7,265
	PC for tSNE	10	20	5
	Total variance explained by PCs selected for tSNE	25	35	26
	Perplexity setting for tSNE	13	15	20
	**Diffusion Map**	**Figure 3H-I**	**Figure 4H-I**	
	Number of PC selected for diffusion map	20	10	
Partial dataset (cell subsets)	**Hierarchical Clustering**	**Figure 3D (GMP only)**	**Figure 1H (mature DC only, all genes)**	
	Cells in analysis	58	88	
	Genes retained for analysis by SC3	6,846	6,838	
	Clustering solutions explored	2:15	2:15	
	Clustering solution	4	8	
	Average Silhouette width	0.5	0.67	

	Highest cluster stability index	0.3	0.8	
	Marker gene p value threshold	0.1	0.01	
	AUROC threshold	0.75	0.85	
	**tSNE**	**Figure S3E-G**		
	Cells in analysis	58		
	Genes in analysis	6,846		
	PC for tSNE	5		
	Total variance explained by PCs selected for tSNE	30		
	Perplexity setting for tSNE	13		
**Partial dataset (cell and gene subsets)**	**Hierarchical Clustering**		**Figure S1K (mature DC only, DC2/3 genes)**	
	Cells in analysis		88	
	Genes retained for analysis by SC3		61 out of 71 genes found in Villani et al.	
	Clustering solutions explored		2:15	
	Clustering solution		7	
	Average Silhouette width		0.53	
	Highest cluster stability index		0.75	
	Marker gene p value threshold		0.05	
	AUROC threshold		0.75	
	**tSNE**		**Figure S1L (71 DC2/3 genes)**	
	Cells in analysis		88	
	Genes in analysis		71	
	PC for tSNE		35	
	Perplexity setting for tSNE		15	

Table S4. Cell input/output and donor details for *in vitro* DC Culture experiments (related to figures 3, 4, S3, S4).

Figure	Input subset	Donor	Input Cell No	Output Cell No				
				Mono	CD14+ DC3	CD14- DC2	cDC1	pDC
CD34+ Progenitor analysis 3C, S3B	Bulk CD34+	1	1000	243	393	7515	1690	408
		1	3258	529	1614	5617	217	12
		1	3258	554	1081	2037	275	9
		2	496	10	343	1332	44	24
		3	500	868	3520	1250	355	50
		3	3090	53	262	742	180	120
		3	3090	27	265	844	145	33
		3	3090	400	374	288	145	65
		4	2600	208	1588	8185	347	179
		4	1642	0	22	1256	125	138
		5	792	6	295	4664	712	106
		6	800	94	328	1583	91	23
		7	6200	355	2250	2556	195	57
		8	3135	45	838	2783	624	50
		8	3135	53	367	1014	279	8
		8	3248	56	61	424	67	14
		9	3056	46	1477	4577	315	51
		10	1712	6	625	706	11	7
		10	2911	29	1574	2650	17	66
		10	2911	802	4996	1925	43	37
		11	848	0	10	120	10	6
		12	1343	5250	5734	1043	0	0
		13	446	22	1854	343	0	0
	CMP	1	3362	122	301	95	0	0
		1	1426	31	272	33	0	0
		2	500	12	0	0	0	0
		4	1558	0	0	1489	0	2
		5	2409	0	210	120	0	0
		14	3040	0	1	0	0	0
		14	1646	51	99	11	1	0
	GMP33+	1	2000	643	4369	11989	1511	51
		2	507	324	1570	397	2	0
		3	500	1538	4467	4346	633	226
		4	1168	142	1572	12966	24	20
		5	1670	144	1169	2211	271	52
		6	669	858	99	39	0	0
		6	70	140	20	0	0	0
	LMPP	1	1500	126	605	3546	3589	827
		1	2957	150	223	1525	1277	757
		2	502	238	618	6479	714	413
		3	428	99	298	7017	4556	3800
		4	215	0	6	9628	358	778
		5	1729	2	21	7262	793	993
		14	1708	7	105	2439	169	86

	GMP33-	1	3042	268	750	456	451	178
		3	558	37	511	3541	2649	480
		4	364	0	246	1148	495	59
		5	848	22	194	2998	437	77
		10	502	0	27	30	36	23
		14	1709	4	64	163	205	34
	GMP123low	3	550	9	278	1502	119	222
		4	419	0	10	192	23	52
		5	491	1	19	363	145	40
	GMP123med	1	700	0	35	271	709	234
		1	2462	0	54	37	466	159
		3	445	0	52	65	394	39
		4	252	0	9	191	45	134
		5	152	0	34	138	190	37
		10	953	0	6	10	20	2
		14	497	0	0	3	4	4
		15	1913	26	75		312	158
**CD34^{med} Precursor analysis 4B, S4C**	123high 303/4low	1	2182	0	12	202	75	191
		1	2182	0	5	150	73	35
		8	1622	0	0	7	40	30
		8	1622	0	1	23	23	2
		16	1521	0	0	2	5	50
		17	1126	0	2	11	1	57
	CD2+ pre-pDC	15	1229	0	0	2	10	181
		16	1005	0	1	2	4	84
		16	1005	0	1	6	1	103
		17	536	0	0	1	0	55
		17	536	0	0	0	0	49
	CD123high CD5+ early pre-DC2	1	434	0	0	10	0	0
		5	444	0	14	65	4	1
		8	469	0	5	41	0	0
		8	1194	0	4	97	1	3
	CD123med CD5+ pre-DC2	3	548	1	113	108	0	3
		4	717	0	0	11	2	1
		8	679	0	2	34	0	0
		8	483	0	0	4	0	0
	CD34med CD123med SIRPA-	1	683	0	16	64	159	0
		9	1596	0	36	54	156	8
		18	630	0	0	16	29	3
		19	1701	0	13	54	144	194
	CD34med SIRPA+	3	3818	16	109	39	18	0
		8	3040	15	27	0	0	0
		10	2703	59	295	29	0	0
		15	1802	0	24	30	0	0
		20	869	18	2557	118	8	0
	SIRPA+CD2+ pre-DC3	3	4045	2	6	1	0	0
		4	329	0	21	17	0	0
		5	2449	18	37	13	0	0
		10	2724	6	6	1	0	0
	SIRPA+CD2- pre-mono	3	4942	14	5	0	0	0
		10	3176	4	3	1	0	0
		15	4966	1	0	0	0	0
		20	1419	6	810	53	1	0

Figure S1, related to Figure 1

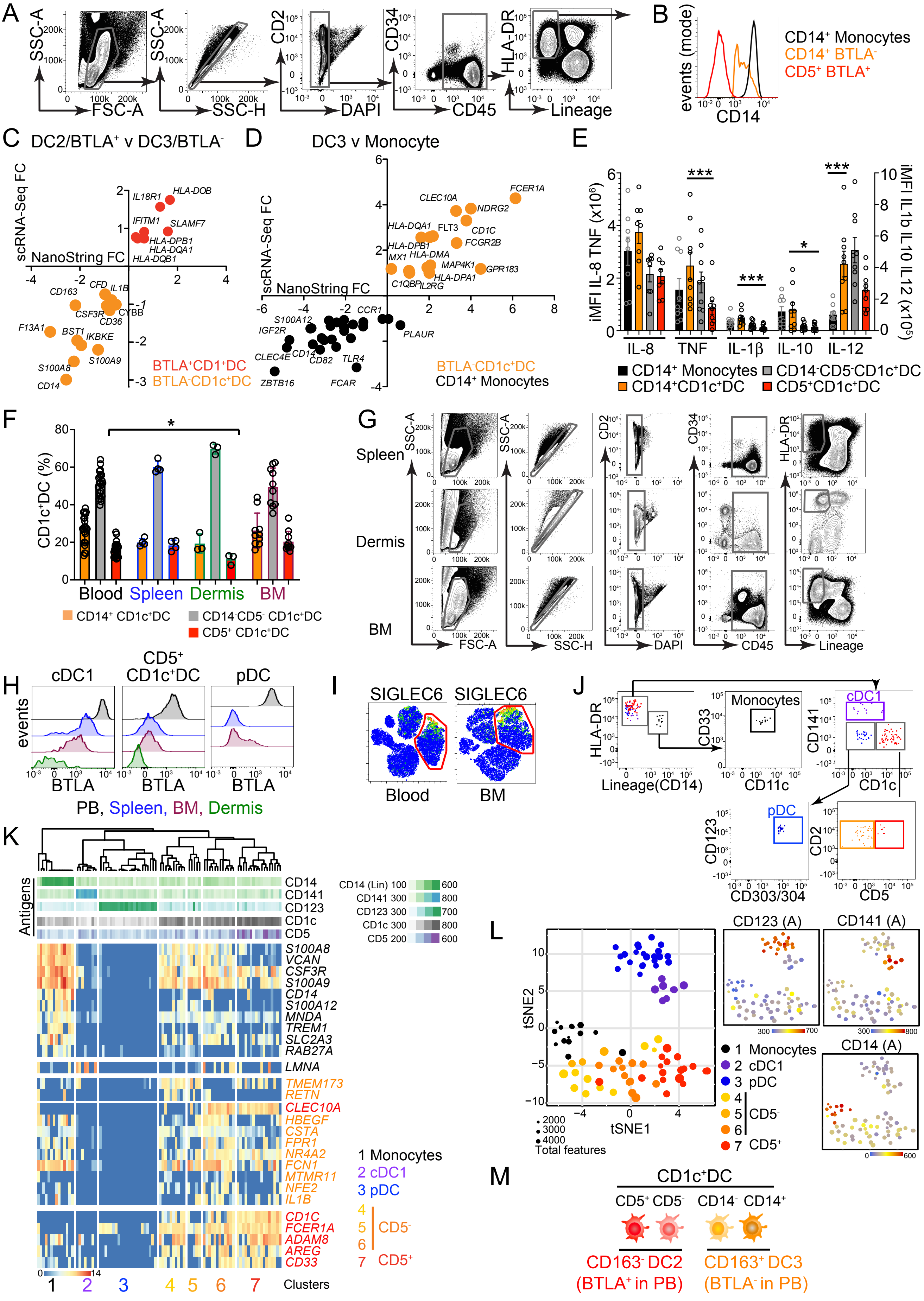

Figure S1: CD1c^{+}DC heterogeneity is evident in human bone marrow

A. Upstream gating steps for the flow cytometric analysis of HC PB monocyte and DC subsets identified PBMC by light scatter properties, excluded doublets, dead and CD45^{-} cells before lin(CD3, CD16, CD19, CD20, CD56)$^{-}$HLA-DR^{+} cells were selected for further analysis in **Figure 1A**. Representative example of n=22

B. Histogram of CD14 expression on CD5^{+}BTLA^{+}CD1c^{+}DCs (red), CD14^{+}CD1c^{+}DCs (orange) and CD14^{+}CD88^{+} monocytes (black), by flow cytometric analysis

C. Correlation of expression fold change of differentially expressed genes by NanoString analysis with the fold change of differentially expressed genes in single cell RNA-Seq analysis described by Villani et al., comparing BTLA^{+}CD1c^{+}DCs (red) (mean of n=3) and BTLA^{-}CD1c^{+}DCs (orange) (mean of n=3) with DC2 and DC3 described by Villani et al. 2017.

D. Correlation of expression fold change of differentially expressed genes by NanoString analysis with the fold change of differentially expressed genes in single cell RNA-Seq analysis described by Villani et al., comparing BTLA^{-}CD1c^{+}DCs(orange) (mean of n=3) and monocytes (black) (mean of n=3) with DC3 and monocytes described by Villani et al. 2017.

E. Intracellular flow cytometric analysis of *in vitro* cytokine elaboration by PB monocytes (black), CD14^{+}CD1c^{+}DC (orange), CD14^{-}CD5^{-}CD1c^{+}DC (gray) and CD5^{+}CD1c^{+}DC (red) from n=9 healthy donors in response to 14hrs stimulation with TLR agonists (CpG, poly(I:C), CL075, LPS). Integrated median fluorescence intensity (iMFI) was calculated by multiplying the frequency of positive cells by the MFI of a given marker. P values were derived from paired two-tailed t-tests (* $p<0.05$; ** $p<0.01$; *** $p<0.005$). Bars show mean±SEM and circles represent individual donors.

F. Relative proportions of CD5^{+}CD1c^{+}DCs (red), CD14^{+}CD1c^{+}DCs (orange) and CD14^{-}CD5^{-}CD1c^{+}DCs (gray) in HC PB (n=22) and BM (n=13) expressed as a percentage of the total CD1c^{+}DC (gated as shown in **Figure 1A**). * $p=0.046$ (Mann Whitney U, two-tailed). Bars show mean±SD and circles represent individual donors.

G. Upstream gating steps for the flow cytometric analysis of HC spleen, skin (dermis) and BM monocyte and DC subsets identified mononuclear cells by light scatter properties, excluded doublets, dead and CD45^{-} cells before lin(CD3, CD16, CD19, CD20, CD56)$^{-}$HLA-DR^{+} cells were selected for further analysis in **Figure 1E** (representative example of n=3 for each tissue) and **F** (representative example of n=13)

H. Histograms of BTLA expression on cDC1, CD5^{+}CD1c^{+}DC and pDC from PB, spleen, BM and dermis (pDC absent), by flow cytometric analysis.

I. tSNE visualization of the expression of TFs IRF4 and IRF8 and surface markers across HC PB and BM lin(CD3,19,20,56,161)$^{-}$HLA-DR^{+} cells by simultaneous CyTOF analysis as in **Figure 1G**. Heat map shows SIGLEC6 expression.

J. Flow cytometric identification of index sorted human BM DC and monocytes for single cell transcriptomic analysis in **Figure 1H** and **S1K,L**.

K. Hierarchical clustering of single cell transcriptomes of mature DC from BM using 71 DC2 and DC3 marker genes identified in Villani et al. to define 7 clusters used to annotate **Figure S1L** (in SC3 software $p<0.05$, AUROC>0.75). Heatmap shows log2 expression. Genes enriched in DC2 or DC3 are shown in red and orange type, respectively. The top rows display fluorescence intensity of surface antigens (‘Antigens’) from index sorted cells.

L. tSNE plots of the first 35 principal components of the DC2 and DC3 single cell transcriptome dataset (as described in **J,K**) of index sorted pDC, cDC1, CD1c^{+}DC and monocytes. The large panel depicts cells annotated by 7 clusters generated in **K**. Smaller panels show the expression of key subset-specific antigens taken from indexed flow cytometry.

M. Schematic of the phenotypic definition of DC2 and DC3 in PB and tissues; CD163^{-} CD5$^{+/-}$(BTLA^{+} in PB) DC2 and CD163^{+}CD14$^{+/-}$(BTLA^{-} in PB) DC3.

Figure S2, related to Figure 2

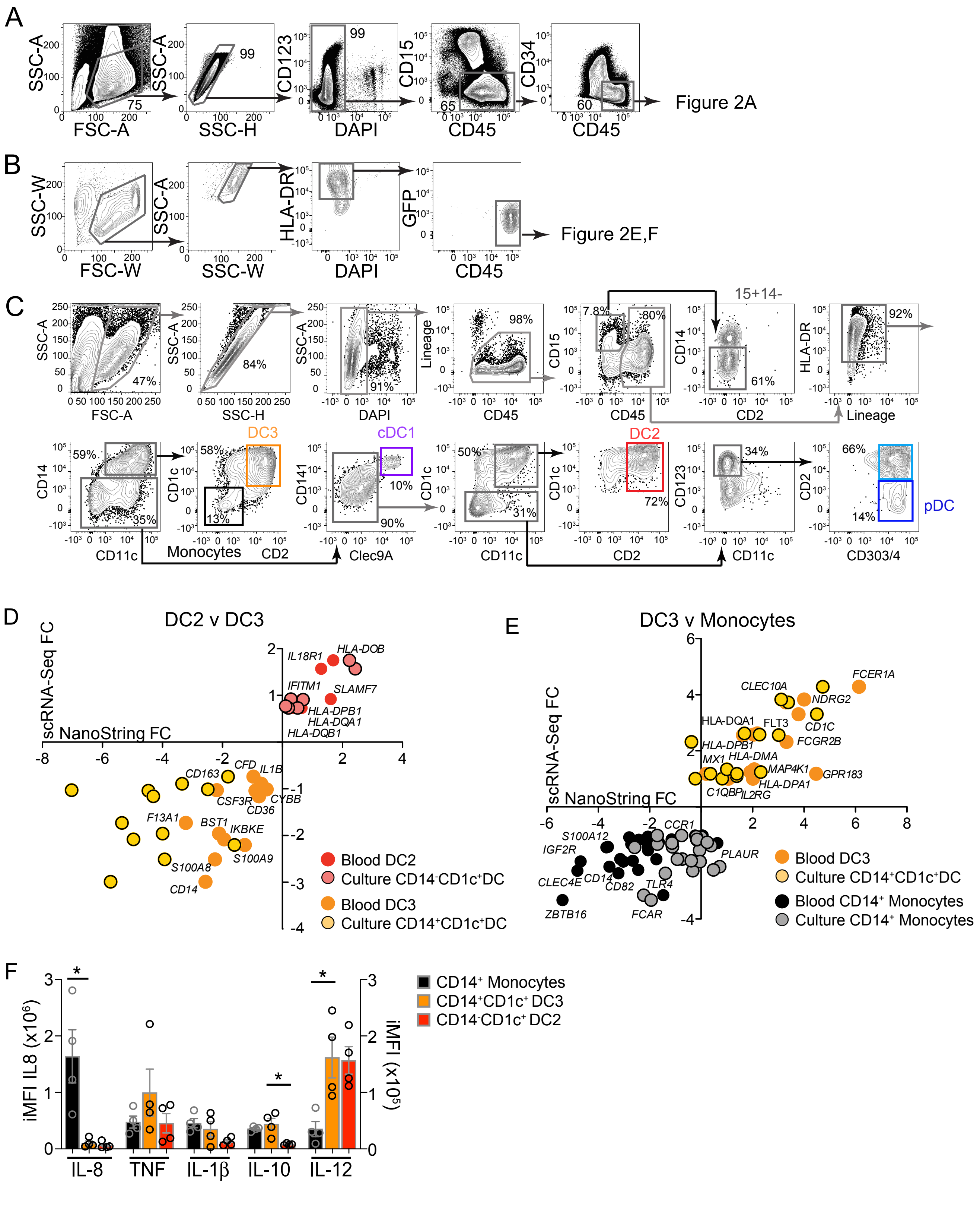

Figure S2: CD14 expression distinguishes between CD1c+DC subsets generated *in vitro*

A. Upstream gating steps for the flow cytometric analysis of *in vitro* derived DC and monocyte subsets. Gates identified live, singlet, cells and excluded $CD15^+$ neutrophils and $CD34^+$progenitors before DC and monocyte identification as shown in **Figure 2A**.

B. Upstream gating steps for the analysis of FACS-purified $CD1c^+$DC subsets after 7 days of culture (**Figure 2E,F**). A representative example of the upstream gating from the culture output of $CD14^-BTLA^-$DC3 (n=7).

C. Full gating strategy for FACS-purification of *in vitro* generated DC and monocyte subsets derived from BM $CD34^+$ progenitors after 21days in culture, for NanoString gene expression analysis (**Figure 2G,H**) and TLR elaboration (**Figure 2I**).

D, E. Correlation of expression fold change of differentially expressed genes by NanoString analysis with the fold change of differentially expressed genes in single cell RNA-Seq described by Villani et al. (in a similar analysis to **Figure S1**), comparing D) DC2 (red) and DC3 (orange) or E) DC3 and monocytes (black). PB DC ($BTLA^+CD1c^+$DC2 and $BTLA^-CD1c^+$DC3) and their culture-derived counterparts ($CD14^-CD1c^+$DC2 and $CD14^+CD1c^+$DC3, outlined dots) are shown. Fold change derived from mean expression of n=3 for PB and n=3 for culture-derived cells.

F. Intracellular flow cytometric analysis of *in vitro* cytokine elaboration by $CD14^+1c^-$ monocytes (black), $CD14^+CD1c^+$DC3 (orange) and $CD14^-CD1c^+$DC2 (red) generated from n=4 BM donors after 21 days in culture, in response to TLR agonists (CpG, poly(I:C), CL075, LPS). Integrated median fluorescence intensity (iMFI) was calculated by multiplying the frequency of positive cells by the MFI of a given marker. P values were derived from paired two-tailed t-tests (* $p<0.05$). Bars show mean±SEM and circles represent individual donors.

Figure S3, related to Figure 3

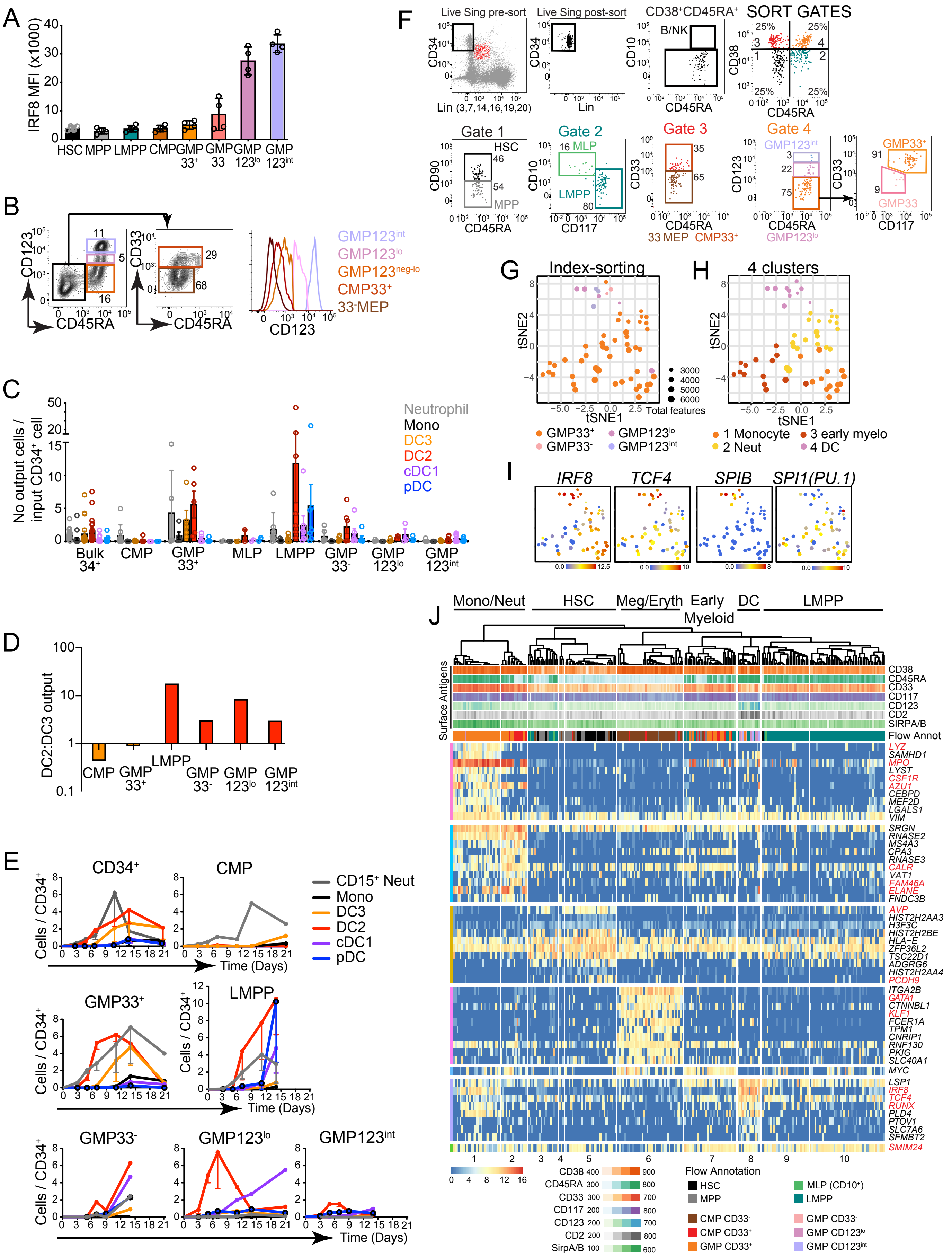

Figure S3. High IRF8 expression defines LMPP-associated DC progenitors

A. Median Fluorescence intensity of Intracellular IRF8 by flow analysis across gates identifying $CD34^{+}$progenitor populations of HC BM (n=4). HSC, hematopoietic stem cell; MPP, multipotent progenitor; MEP, megakaryocyte erythroid progenitor; MLP, multilymphoid progenitor; LMPP, lymphoid primed multipotent progenitor; CMP, common myeloid progenitor; GMP, granulocyte macrophage progenitor. Bars represent mean±SD.

B. Flow analysis of $CD34^{+}CD38^{+}CD10^{-}$ progenitor populations (as shown in **Figure 3A**) showing the relative levels of CD123 expression across $CD33^{+}CD123^{neg\text{-}lo}$ (predicted CMP) and $CD33^{-}CD123^{-}$ (predicted MEP) $CD45RA^{-}$ populations and $CD45RA^{+}$GMP fractions.

C. Absolute proliferative capacity and differentiation potential of progenitor fractions subjected to 14 days in DC differentiation culture, as depicted in **Figure 3C**, expressed as the number of cells generated per input $CD34^{+}$ cell. $CD15^{+}$ neutrophils are included. Bars show mean±SEM, circles represent individual donors.

D. The relative output (ratio) of DC2 and DC3 from the specified $CD34^{+}$progenitor fractions. Orange bars DC2<DC3; red bars DC2>DC3.

E. Kinetics of DC, monocyte and neutrophil output from progenitor fractions over 14-21 days in DC differentiation culture. $CD34^{+}$ n=3 (n=2-3/timepoint), CMP n=1, $GMP33^{+}$ n=3 (n=1-3/timepoint), LMPP n=3 (n=2-3/timepoint), $GMP33^{-}$ n=3 (n=1-2/timepoint), $GMP123^{lo}$ n=2 (n=1-2/timepoint), $GMP123^{int}$ n=3 (n=1-3/timepoint). Dots and bars represent mean–SEM.

F. Flow identification of index sorted human BM progenitors for single cell transcriptomic analysis in **Figures 3D-J and S3G-J.** A tight lin(CD3, 7, 14, 16, 19, 20)$^{-}$ gate was used to select $CD34^{+}$ progenitors for single cell sorting. This excluded $lineage^{lo}$ $CD10^{+}$B/NK progenitors, as defined in **Figure 3A**, shown backgated (red dots) onto the pre-sort BM live, singlet, mononuclear cells (gray). Top middle panel shows the same $Lin^{-}34^{+}$ gate on sorted single cells. Top right panel demonstrates the absence of $CD10^{+}$B/NK cells in the sorted $CD38^{+}CD45RA^{+}$ population.

G-I. tSNE visualization of the first 5 principal components (30% total variance) of the transcriptomes of 58 single GMP, analyzed independently of surface phenotype. tSNE plots are annotated by **G**, gate of origin from index-linked flow (**Figure S3F**) or **H**, 4 hierarchical clusters (**Figure 3D**) showing clustering of cells from the $CD33^{-}$, $CD123^{lo}$, and $CD123^{int}$ gates (cluster 4) away from clusters 1-3 containing $GMP33^{+}$ cells. Heatmaps (**I**) show log2 expression of key DC TF genes on tSNE plot in **G** and **H**.

J. Unsupervised hierarchical clustering of single cell transcriptomes of all progenitor cells, using all protein-coding, non-cell cycle genes (independent of surface antigen expression). Marker genes for 10 clusters generated within SC3 ($p<0.01$, AUROC>0.75) are displayed, examples of which identify cluster 1, monocyte (*LYZ*, *CSF1R*); cluster 2, granulocyte (*CALR*, *ELANE*), clusters 3-5, HSC and MPP (*AVP*, *PCDH9*), cluster 6, MEP (*GATA1*, *KLF1*), cluster 8, DC (*IRF8*, *TCF4*, *RUNX*), clusters 9-10, LMPP (*SMIM24*). Heatmap shows log2 gene expression. The top rows display fluorescence intensity of surface antigens ('Surface Antigens') from index sorted cells, 'Flow annotation (Flow annot)' denotes the classification of index sorted cells by their surface phenotype (**Figure S3F**).

Figure S4, related to Figure 4

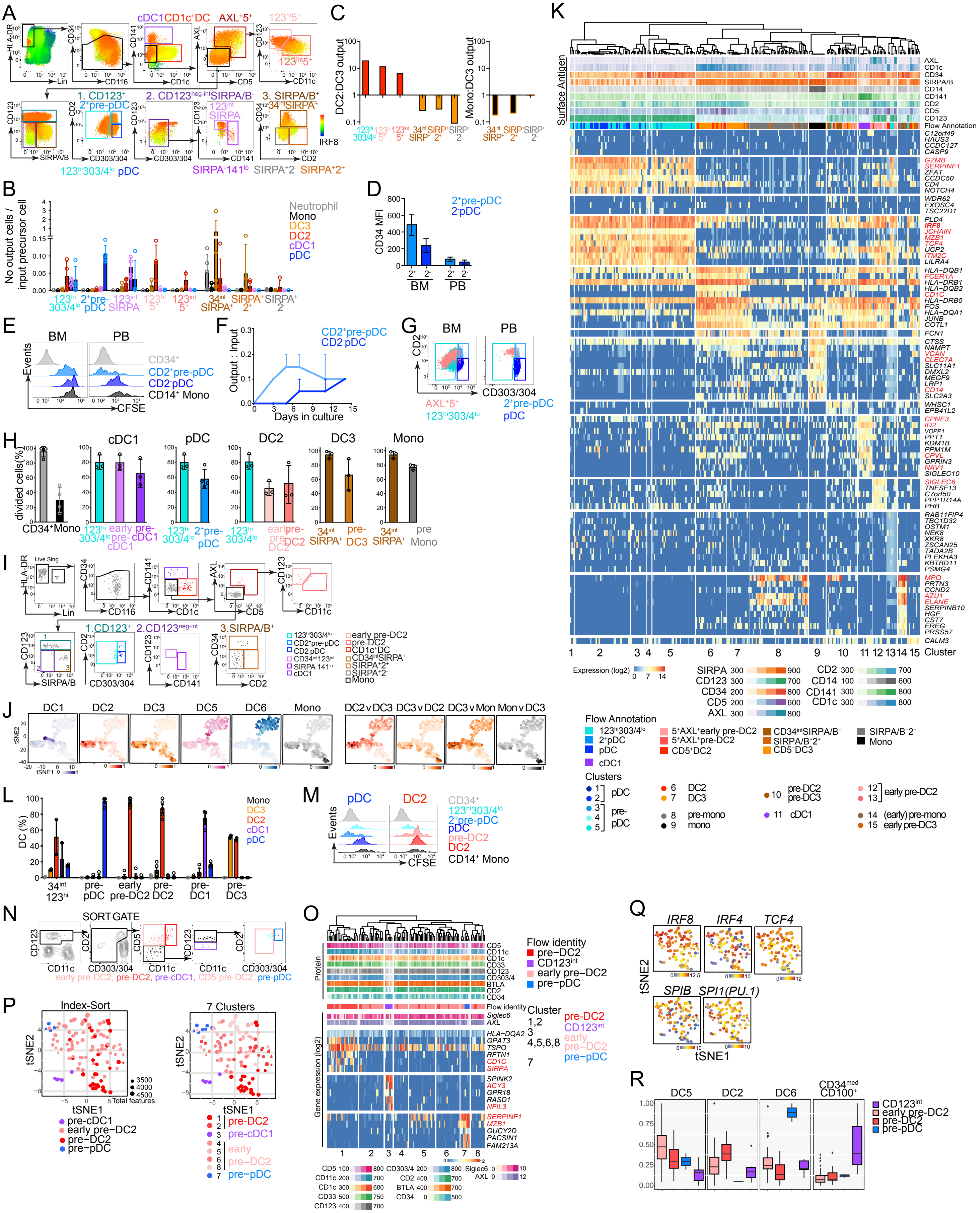

Figure S4. Two trajectories of DC development connect the progenitor compartment with mature DC

A. Heatmaps showing IRF8 expression by intracellular flow cytometric analysis across sequential bivariate plots, using the gating strategy to identify DC and pre-DC shown in **Figure 4A**. Heatmaps generated in FlowJo 10.6.1.

B. Absolute proliferative capacity and differentiation potential of $CD34_{int}$ precursor fractions subjected to 14 days in DC differentiation culture, as depicted in **Figure 4C**, expressed as the number of cells generated per input precursor cell. $CD15_{+}$ neutrophils are included. Bars show mean±SEM, circles represent individual donors.

C. The relative output (ratio) of DC2 versus DC3 (left graph) or Monocyte versus DC3 (right graph) from the specified $CD34_{int}$ precursor fractions. Red bars DC2>DC3; orange bars DC3>DC2, black bars with orange outline DC3>monocyte.

D. Mean fluorescence intensity (MFI) of CD34 by flow cytometric analysis of $CD2_{+}$ and $CD2_{-}$ pDC from BMMC (n=3) and PBMC (n=3). Plots show mean±SEM.

E. CFSE dilution in $CD2_{+}$ and $CD2_{-}$ pDC from BM and PB after 3 days in DC culture, to assess proliferation. Histograms show $CD34_{+}$ progenitors and monocytes as controls. Plots shown are representative of n=3 experiments in PB and BM.

F. Kinetics of the output generated from 14 days culture of $CD2_{+}$ pDC, expressed as the number of cells generated per input cell. n=2 donors with n=2 analyses at each time point. Dots and bars show mean+SEM.

G. Flow cytometric analysis of $Lin_{-}HLA\text{-}DR_{+}CD1c_{-}CD141_{-}CD123_{hi}$ PBMC and BMMC showing the location of $AXL_{+}CD5_{+}CD123_{+}CD11c_{-}$ early pre-DC2 (pink) on the bivariate plot of CD2 versus CD303/4, overlying the $CD34_{int}\,CD123_{hi}CD303/4_{lo}$ population (turquoise).

H. Summary of the proliferative capacity of BM DC precursors. BMMCs were CFSE stained then precursors FACS-purified and cultured in DC-culture conditions for 3 days before flow cytometric analysis of CFSE. Bulk $CD34_{+}$ progenitors and $CD14_{+}$ monocytes were included as positive and negative controls, respectively. Bars represent mean±SD of n=3-4 donors, circles represent individual donors.

I. Location, in flow cytometry parameter space, of index-sorted precursor cells purified for scRNA-Seq depicted in **Figure 4E-J, S4J-K)**.

J. DC subset signature scores generated from Villani et al, mapped across the tSNE plot of precursor and mature DC single cell transcriptomes (**Figure 4E-J**). To generate signature scores, the dataset was mined for expression of cell subset 'signature' genes defined by single cell RNA-Seq in Villani et al., including DC1 (cDC1), DC2 (cDC2a), DC3 (cDC2b), DC5 (AXL_{+}), DC6 (pDC) and monocytes. Normalized counts were then rescaled from 0 to 1 and the average scaled score displayed on t-SNE plots generated as described in the STAR methods. The left panel shows the level of expression of DC subset signature scores. The right panel shows the mapping across tSNE space of similar scores generated from differentially expressed genes after pairwise comparisons of subsets, as indicated.

K. Unsupervised hierarchical clustering of single cell transcriptomes of BM precursor and mature DC subsets and CD14^{+} monocytes, using all protein-coding, non-cell cycle genes (independent of surface antigen expression). Marker genes for 15 clusters generated within SC3 ($p<0.01$, AUROC>0.85) are shown. Examples used to identify clusters are marked in red and derived from subset signature genes in Villani et al.. Clusters were used to annotate **Figure 4F, H**. The top rows display fluorescence intensity of surface antigens (‘Surface Antigens’) from index sorted cells. ‘Flow Annotation’ denotes the classification of index sorted cells by their surface phenotype (in **Figure S4I**), Cluster color code related to **Figure 4F,H**.

L. The output of *in vitro* culture of DC precursors from PB, FACS-purified using the gating strategy shown in **Figure 4C** showing lineage-specific enrichment from precursors, analogous to the output from phenotypically similar BM precursors. Output is expressed as the proportion of each DC and monocyte population as a percentage of the total cells captured by all DC and monocyte gates. n=3-5 donors for each population. Bars represent mean+SEM, circles represent individual experiments.

M. Proliferative potential of PB DC precursors at day 3 of culture, estimated by CFSE dilution. Bulk CD34^{+} progenitors and CD14^{+}monocytes were included as positive and negative controls, respectively. The CFSE dilution histograms for each precursor are grouped and ordered according to their proposed position in the developmental trajectory for pDC or DC2 lineages. Results are representative of n=3 experiments.

N. Sorting strategy applied to Lin^{-}HLA-DR^{+} CD1c^{-}CD141^{-} cells, and location, in flow cytometry parameter space, of subsequently index-sorted PB precursor cells (CD2^{-} pDC excluded) purified for scRNA-Seq analysis depicted in **Figure S4O-R**.

O. Hierarchical clustering of single cell transcriptomes of PB pre-DC populations showing signature genes that identify 8 clusters ($p<0.05$ and AUROC>0.7), used to annotate **Figure S4P**. These defined CD11c^{+} pre-DC2 (cluster 1 and 2; marked by *CD1c* and *SIRPA* at RNA level), AXL^{+}CD5$^{+/-}$CD11c^{lo} early pre-DC2 (clusters 4,5,6,8), CD34^{int}CD123^{int}AXL^{-} cells, marked by *NFIL3* (critical for cDC1 development, Bagadia et al., 2019) and shown to have enriched cDC1 potential in culture (**Figure S4L**) and a small number of AXL^{-}CD5^{-}CD303/4^{hi} pre-pDC (contained within cluster 7, marked by *SERPINF1*, *MZB1*).

P. tSNE of the first 5 principal components (26% total variance) of 116 single cell transcriptomes sampled from PB pre-DC populations, annotated by the gate of origin from index-linked flow cytometry (Index Sort) or by 7 clusters generated from hierarchical clustering **Figure S4O**. Pre-DC2 and pre-pDC were polarized in tSNE space, linked by early pre-DC2 which showed variable expression of both pDC and CD1c^{+}DC genes.

Q. Heatmaps show expression of key TF on the tSNE plot from **Figure S4P**.

R. Mapping of single cell transcriptomes to cell populations identified by Villani et al. Cells identified as early pre-DC2 and pre-DC2(CD123^{+}CD5^{+}CD303/4^{lo}) showed enrichment of gene expression with ‘AS’ DC (DC5) and DC2; pre-pDC (CD123^{+}CD2^{+}CD303/4^{hi}) mapped to pDC (DC6) and the CD34^{int}CD123^{int}SIRPA^{-} population (early pre-cDC1) showed enrichment of genes expressed by a CD34^{int}CD100^{+} population also found in Villani et al. to contain cDC1 potential *in vitro*.

Figure S5, related to Figure 5

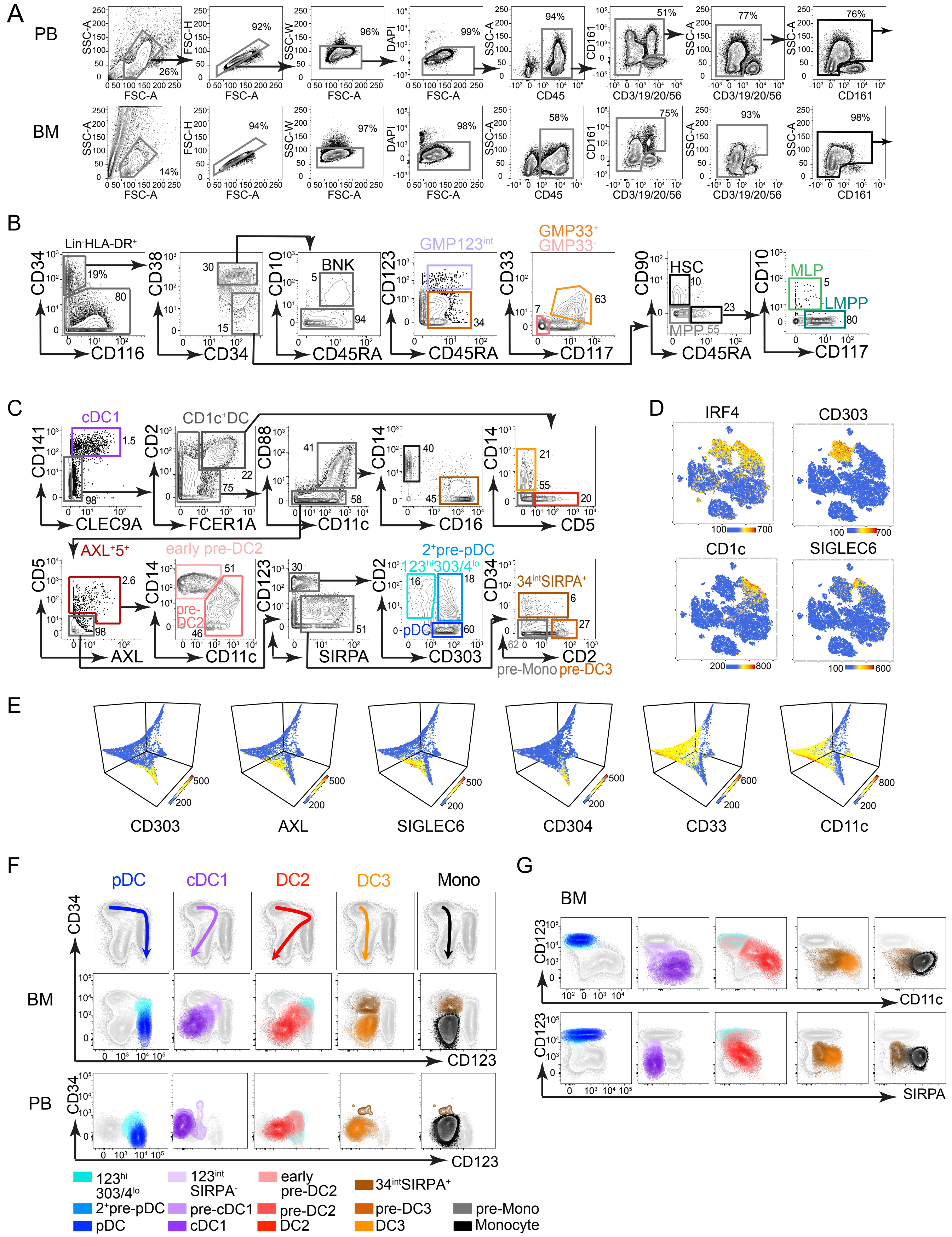

Figure S5. Differential IRF8 expression defines the two trajectories of DC development

A. Flow cytometric gating strategy for the FACS-purification of PBMC and BMMC to enrich for $CD45^+$ cells and exclude lin(CD3,19,20,56,161)$^+$ lymphocytes prior to CyTOF analysis (**Figure 5A-E**).

B. Gating of bivariate plots from CyTOF analysis to identify $CD34^+$ progenitor subsets, comparable with flow cytometric analysis in **Figure 3A**.

C. Gating of bivariate plots from CyTOF analysis to identify DC and monocyte subsets and their precursors, comparable with flow cytometric analysis in **Figure 4A**.

D. Heatmap expression of IRF4 and additional key antigens (CD303, CD1c, SIGLEC6) across the tSNE visualization plot shown in **Figure 5A-E**.

E. Heatmaps of the expression (log2) of additional key antigens superimposed across the diffusion map trajectories generated with 14,000 GMP, precursor and mature DC and monocyte cells and 29 markers to infer pseudo-temporal ordering of cells and reconstruct lineage branching, as shown in **Figure 5F**.

F. Back-gating of $CD34_{neg\text{-}int}$ DC precursors and DC subsets (defined in **Figure 4A**) on bivariate plots, to relate the DC developmental pathways to standard flow analysis. The relative expression of CD34 and CD123 is visualized across populations comprising lineage-specific developmental pathways as defined by the previous data. Schematic arrows summarize the proposed sequence of maturation of gated populations across the 2D space, from $CD34^+$ progenitor compartment to mature DC populations. The utility of BM as a source material and relative paucity of DC precursors in PB is also illustrated.

G. Lineage-specific $CD34_{neg\text{-}int}$ DC precursors and DC subsets from BM, as defined in **Figure 4A**, backgated onto bivariate plots of CD123 versus CD11c and CD123 versus SIRPA/B to visualize the relative expression of these antigens on populations comprising lineage-specific developmental pathways.

Figure S6, related to Figure 6

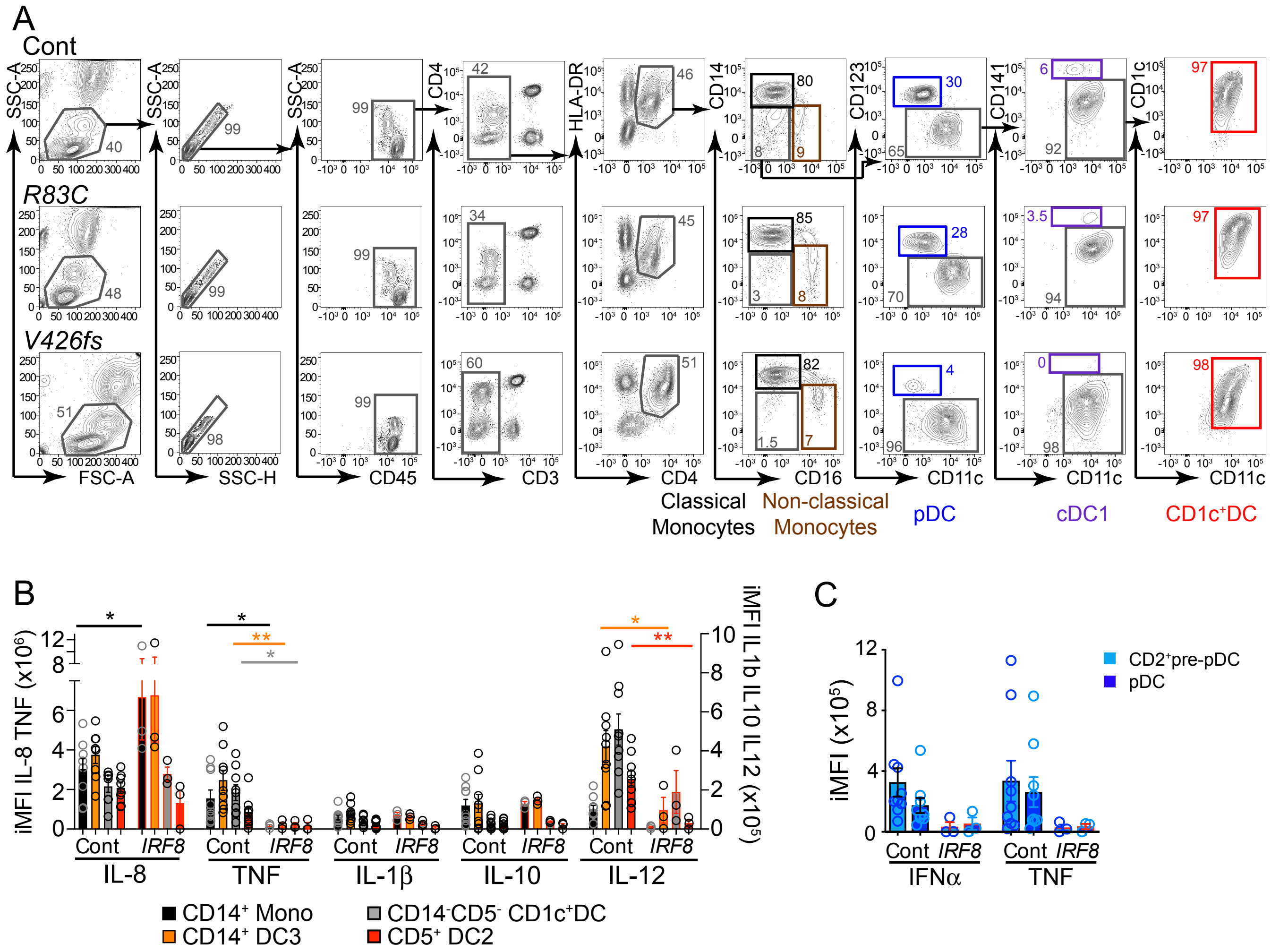

Figure S6. IRF8^{hi} and IRF8^{lo} pathways are differentially compromised in IRF8 deficiency

A. Flow cytometric gating strategy for whole PB Trucount™ analysis of a HC (Cont), subject carrying heterozygous *IRF8* mutation (*R83C*) and subject carrying dominant negative mutation (*V426fs*), summarized in **Figure 6B**. Numbers represent the percent of cells from the parent gate.

B-C. Intracellular flow cytometric analysis of *in vitro* cytokine elaboration by monocytes (black bars), CD14^{+}DC3 (orange), CD14^{-}CD5^{-}CD1c^{+}DC (gray) and CD5^{+}DC2 (red) (B) and CD2^{+}pre-pDC and pDC (C) from HC (n=8) and subjects carrying heterozygous *IRF8* mutations (*R83C*, *R291Q*, mean of technical duplicates; and *V426fs*) in response to 14hrs stimulation with TLR agonists (CpG, poly(I:C), CL075, LPS). Integrated median fluorescence intensity (iMFI) was calculated by multiplying the frequency of positive cells by the MFI of a given marker. Bars show mean±SEM. P values from Mann Whitney U analysis (* p<0.05, **p<0.01, ***p<0.001).

Figure S7, related to Figure 7

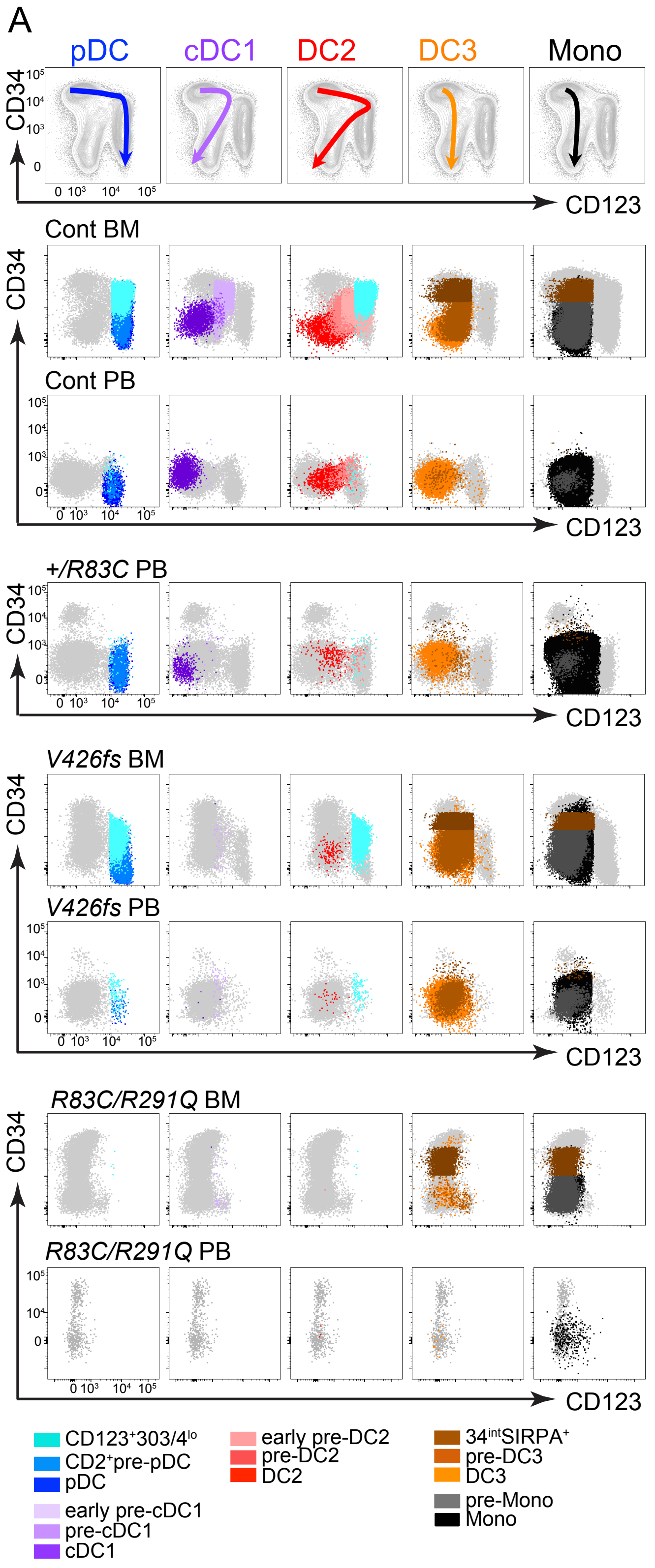

Figure S7. IRF8 deficiency causes dose-dependent blockade of the IRF8$_{hi}$ pathway

A. Summary of DC and monocyte differentiation pathways from BM (where available) and PB of subjects carrying heterozygous *R83C*, dominant negative *V426fs* and bi-allelic *R83C/R291Q IRF8* mutations. DC and precursor populations were gated as shown in **Figure 7B**. These were backgated on bivariate plots to visualize the relative expression of CD34 and CD123 on populations comprising lineage-specific developmental pathways. Arrows indicate the proposed sequence of maturation of gated populations from CD34^{+} progenitor compartment to mature DC populations.
