# Supplementary material for: Differential IRF8 Transcription Factor Requirement Defines Two Pathways of Dendritic Cell Development in Humans
Source: Immunity. 2020 Aug 18;53(2):353–370.e8. doi: 10.1016/j.immuni.2020.07.003 (PMC7447982; doi:10.1016/j.immuni.2020.07.003)
Supplement: Document S2. Article plus Supplemental Information [file mmc5.pdf]

Immunity

Article

Differential IRF8 Transcription Factor Requirement Defines Two Pathways of Dendritic Cell Development in Humans

Graphical Abstract

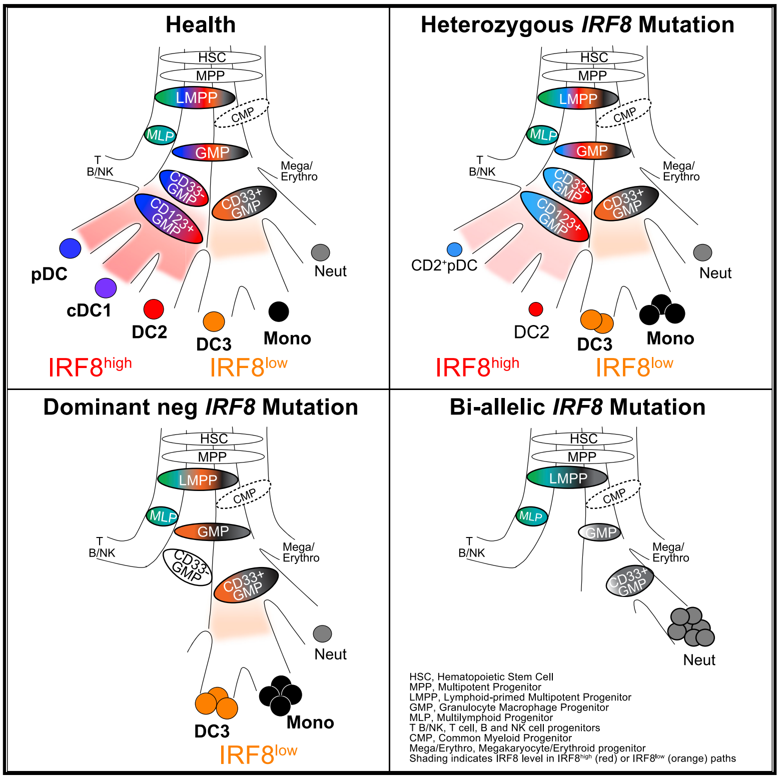

Highlights

- Distinct development trajectories of DC2 and DC3 underpin human cDC2 heterogeneity
- pDC, cDC1, and DC2 (classical DCs) develop from LMPPs along a CD123^{+} IRF8^{high} pathway
- DC3 and monocytes develop from CD33^{+} GMPs along an IRF8^{low} SIRPA^{+} pathway
- IRF8 deficiency causes gene dose-dependent loss of IRF8^{high} then IRF8^{low} pathway DCs

Authors

Urszula Cytlak, Anastasia Resteu, Sarah Pagan, ..., Gina Doody, Matthew Collin, Venetia Bigley

Correspondence

venetia.bigley@ncl.ac.uk

In Brief

Heterogeneity of human CD1c^{+} dendritic cells (cDC2s) is described, but how this arises is unknown. Cytlak and colleagues demonstrate that the cDC2 subsets, DC2 and DC3, develop along distinct hematopoietic trajectories, defined by differential IRF8 expression. DC2s develop from LMPPs along an IRF8^{hi} pathway, while DC3 differentiation follows an IRF8^{low} trajectory.

Cytlak et al., 2020, Immunity *53*, 353–370
August 18, 2020 © 2020 The Authors. Published by Elsevier Inc.
https://doi.org/10.1016/j.immuni.2020.07.003

CellPress

Immunity

CellPress
OPEN ACCESS

Article

Differential IRF8 Transcription Factor Requirement Defines Two Pathways of Dendritic Cell Development in Humans

Urszula Cytlak,[1,12] Anastasia Resteu,[1,12] Sarah Pagan,[1] Kile Green,[1] Paul Milne,[1] Sheetal Maisuria,[2] David McDonald,[3] Gillian Hulme,[3] Andrew Filby,[3] Benjamin Carpenter,[4] Rachel Queen,[3] Sophie Hambleton,[1,5] Rosie Hague,[6] Hana Lango Allen,[7,8] James E.D. Thaventhiran,[9] Gina Doody,[10] Matthew Collin,[1,11,13] and Venetia Bigley[1,11,13,14,*]
[1]Translational and Clinical Research Institute, Newcastle University, Newcastle upon Tyne NE2 4HH, UK
[2]Institute of Rheumatic and Musculoskeletal Medicine, University of Leeds, Leeds LS9 7TF, UK
[3]Biosciences Institute, Newcastle University, Newcastle upon Tyne NE2 4HH, UK
[4]Oxford Genomics Centre, Wellcome Centre for Human Genetics, Nuffield Department of Medicine, University of Oxford, Oxford OX3 7BN, UK
[5]Great North Children's Hospital, Newcastle upon Tyne Hospitals NHS Foundation Trust, Newcastle upon Tyne NE1 4LP, UK
[6]Department of Paediatric Immunology and Infectious Diseases, Royal Hospital for Children, Glasgow G51 4TF, UK
[7]Department of Haematology, University of Cambridge, Cambridge Biomedical Campus, Cambridge CB2 0SP, UK
[8]NIHR BioResource, Cambridge University Hospitals, Cambridge Biomedical Campus, Cambridge CB2 0SP, UK
[9]MRC Toxicology Unit, School of Biological Sciences, University of Cambridge, Cambridge CB2 1QR, UK
[10]Leeds Institute of Medical Research, University of Leeds, Leeds LS9 7TF, UK
[11]Northern Centre for Cancer Care, Newcastle upon Tyne Hospitals NHS Foundation Trust, Newcastle upon Tyne NE7 7DN, UK
[12]These authors contributed equally
[13]Senior author
[14]Lead Contact
*Correspondence: venetia.bigley@ncl.ac.uk
https://doi.org/10.1016/j.immuni.2020.07.003

SUMMARY

The formation of mammalian dendritic cells (DCs) is controlled by multiple hematopoietic transcription factors, including IRF8. Loss of IRF8 exerts a differential effect on DC subsets, including plasmacytoid DCs (pDCs) and the classical DC lineages cDC1 and cDC2. In humans, cDC2-related subsets have been described including AXL$^+$SIGLEC6$^+$ pre-DC, DC2 and DC3. The origin of this heterogeneity is unknown. Using high-dimensional analysis, *in vitro* differentiation, and an allelic series of human IRF8 deficiency, we demonstrated that cDC2 (CD1c$^+$DC) heterogeneity originates from two distinct pathways of development. The lymphoid-primed IRF8^{hi} pathway, marked by CD123 and BTLA, carried pDC, cDC1, and DC2 trajectories, while the common myeloid IRF8^{lo} pathway, expressing SIRPA, formed DC3s and monocytes. We traced distinct trajectories through the granulocyte-macrophage progenitor (GMP) compartment showing that AXL$^+$SIGLEC6$^+$ pre-DCs mapped exclusively to the DC2 pathway. In keeping with their lower requirement for IRF8, DC3s expand to replace DC2s in human partial IRF8 deficiency.

INTRODUCTION

The hematopoiesis of dendritic cells (DCs) is controlled by a network of transcription factors (TFs), including GATA2, SPI1 (PU.1), TCF4 (E2-2), ZEB2, IRF4, IRF8, and IKZF1 (IKAROS) (Murphy et al., 2016; Collin and Bigley, 2018). Critical roles have been demonstrated in humans for GATA2 (Dickinson et al., 2014), IRF8 (Hambleton et al., 2011; Bigley et al., 2018), and IKZF1 (Cytlak et al., 2018). DC potential traverses the phenotypic space of hematopoietic stem cells (HSCs), multipotent progenitors (MPPs), common myeloid progenitors (CMPs), lymphoid-primed multipotent progenitors (LMPPs), and granulocyte-macrophage progenitors (GMPs) (Lee et al., 2015, 2017; Helft et al., 2017). Single-cell cloning experiments demonstrate oligo- and unipotent differentiation pathways and highlight critical interactions between TFs such as SPI1 (PU.1) and IRF8 in priming and directing DC development (Lee et al., 2017; Velten et al., 2017; Giladi et al., 2018).

Functionally distinct populations of DCs arise directly from hematopoiesis itself (Lee et al., 2017; See et al., 2017; Villani et al., 2017). Plasmacytoid DCs (pDCs) are distinct from myeloid or classical DCs (cDCs), comprising two subsets, cDC1s and cDC2s, evolutionarily conserved across mammalian species (Guilliams et al., 2016; Granot et al., 2017). DC potential is found in CD123$^+$ regions of human GMPs (Lee et al., 2015; Helft et al., 2017), where most cells have unipotential fates for pDCs, cDC1s,

This is an open access article under the CC BY license (http://creativecommons.org/licenses/by/4.0/).

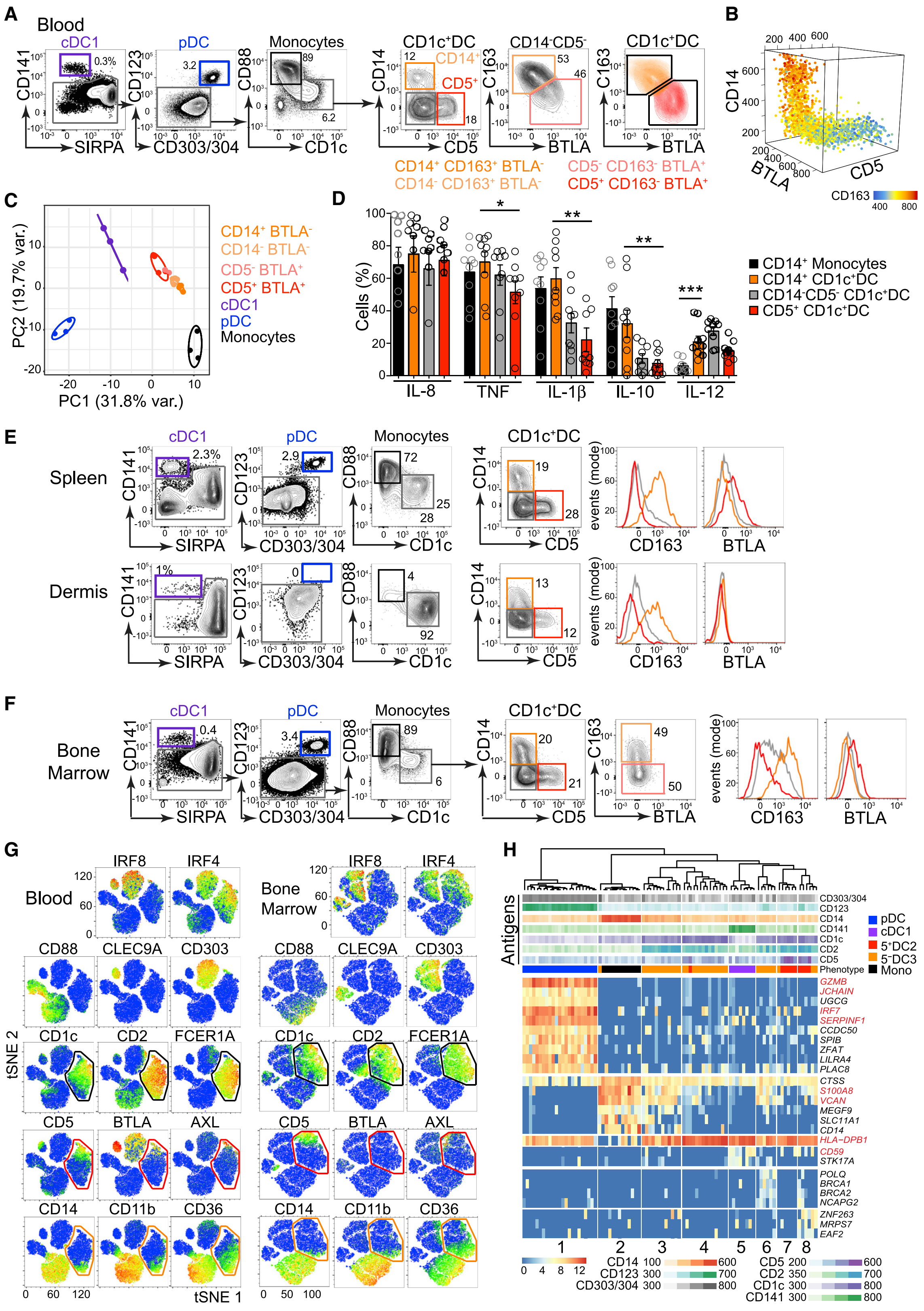

(legend on next page)

or cDC2s (Lee et al., 2017). These observations are more consistent with contemporary lineage-primed models of hematopoiesis in which cell fate specification occurs in the early stem and progenitor cell compartments and development progresses along increasingly stable unipotent trajectories (Naik et al., 2013; Notta et al., 2016; Velten et al., 2017; Giladi et al., 2018; Laurenti and Göttgens, 2018). However, the phenotypic identities of GMPs that contain discrete DC potentials leading to pDCs, cDC1s, and cDC2s in human have not been described.

Human cDC2s, hereafter referred to as CD1c$^+$ DCs, comprise two subpopulations in peripheral blood (PB), one closer in gene expression and function to cDC1s and the other to monocytes (Schrøder et al., 2016; Yin et al., 2017; Alcántara-Hernández et al., 2017; Korenfeld et al., 2017; Villani et al., 2017). However, it is not known if both types of CD1c$^+$ DC arise from distinct lineage trajectories, differentially regulated by TFs, or whether they represent two alternative transcriptional states of a common lineage originating from the CD123$^+$ GMP.

IRF8 plays a major role in DC development. In mice, it is required for normal development of cDC1s and pDCs (Tailor et al., 2008; Grajales-Reyes et al., 2015; Sichien et al., 2016). Acting at multiple stages, it balances neutrophil, monocyte, and DC fate in combination with the TFs CEBPα and PU.1 (Lee et al., 2017; Giladi et al., 2018; Becker et al., 2012; Kurotaki et al., 2014). In common with other TFs regulated by super-enhancers, IRF8 effects are dose-dependent (Afzali et al., 2017).

We have previously described two humans with bi-allelic *IRF8* mutations (*IRF8$^{K108E/K108E}$* and *IRF8$^{R83C/R291Q}$*) with a complete absence of monocytes and DCs (Hambleton et al., 2011; Bigley et al., 2018). *K108E* mutation results in loss of nuclear localization and transcriptional activity, concomitant with decreased protein stability (Salem et al., 2014). *R291Q* is orthologous to *R294*, mutated in the BXH2 Irf8-deficient mouse. *R83C* shows reduced nuclear translocation, and neither *R291Q* nor *R83C* is able to regulate the Ets-IRF composite element or interferon (IFN)-stimulated response element, although *R291Q* retains BATF-JUN interactions *in vitro* (Bigley et al., 2018). The heterozygous parents of these individuals, together with a new kindred affected by an intermediate autosomal-dominant phenotype caused by a frameshift at *V426*, provide an allelic series of IRF8 activity.

In the present study, we use *in vitro* cultures, single-cell analysis, and the series of human *IRF8* variants to resolve two discrete pathways of DC development differentially dependent upon IRF8, each forming distinct subsets of the CD1c$^+$ DC population. The IRF8^{hi} pathway is linked to a classical pathway shared by cDC1s and pDCs. The IRF8^{lo} pathway is linked to the development of monocytes.

RESULTS

CD1c$^+$DC Heterogeneity Is Evident in Human Bone Marrow

We first sought to define CD1c$^+$ DC heterogeneity in healthy control (HC) human PB by conventional flow cytometry. This revealed differential expression of monocyte-related antigens CD14 and CD163 and lymphoid-associated antigens CD5 and BTLA (Figures 1A, 1B, and S1A) within the CD1c$^+$ DC population. CD14 and CD5 expression marked the poles of a phenotypic continuum and CD163$^+$BTLA$^-$ and CD163$^-$BTLA$^+$ populations were identifiable within the CD14$^-$CD5$^-$ gate. Notably, CD14 expression on CD14$^+$CD1c$^+$ DCs is at least 1 log lower than on classical monocytes (Figure S1B), which were excluded by CD88 expression. This continuum was mirrored at the transcriptomic level (Figure 1C) and was concordant with the differential expression of genes distinguishing DC2s from DC3s and DC3s from monocytes, as described previously (Villani et al., 2017; Figures S1C and S1D; Table S1). In response to Toll-like receptor (TLR) stimulation, all fractions of CD1c$^+$ DCs were able to elaborate interleukin-12 (IL-12), in contrast to monocytes. However, the monocyte-related cytokines IL-1β and IL-10 were produced by CD14$^+$CD1c$^+$ DCs (Figures 1D and S1E).

CD5$^+$ and CD14$^+$ CD1c$^+$ DC subsets, with differential CD163 expression, were present in HC spleen and dermis (Figures 1E, S1F, and S1G). However, BTLA expression was much lower in spleen and only just detectable in dermis (Figures 1E and S1H). Bone marrow (BM) also contained homologous populations, although BTLA was not well expressed in this tissue (Figures 1F and S1F–S1H). To simultaneously interrogate PB and BM, the panel was extended using mass cytometry (cytometry by time of flight [CyTOF]). CD1c$^+$ DCs were delineated by the expression

Figure 1. CD1c$^+$ DC Heterogeneity Is Evident in Human BM

(A) Flow phenotyping of CD1c$^+$ DCs from HC PB mononuclear cells (PBMCs) (representative example of n = 22), distinct from SIRPA$^-$CD141$^+$ cDC1s, CD123$^+$CD303/4$^+$ pDCs, and CD88$^+$monocytes (Mono). CD14$^+$CD163$^+$BTLA$^-$ (orange), CD14$^-$CD163$^+$BTLA$^-$ (light orange), CD163$^-$BTLA$^+$CD5$^-$ (light red), and CD163$^-$BTLA$^+$CD5$^+$ (red) CD1c$^+$ DC subsets are indicated.

(B) 3D representation of CD14, CD5, and BTLA expression (flow cytometry) across the CD1c$^+$ DC population. Heatmap shows expression of CD163.

(C) PCA of NanoString gene expression profiling of fluorescence-activated cell sorting (FACS)-purified DC subsets from n = 3 HC PBMCs. CD1c$^+$ DCs were purified based on their expression of CD14, CD5, and BTLA (A).

(D) Intracellular flow analysis of *in vitro* cytokine elaboration (percentage of positive cells) by PB monocytes (black) and CD1c$^+$DC subsets CD14$^+$ (orange), CD14$^-$CD5$^-$ (gray), and CD5$^+$ (red) from n = 9 HC donors in response to 14-h stimulation with TLR agonists (CpG, poly(I:C), CL075, and lipopolysaccharide [LPS]). p values were derived from paired two-tailed t tests; *p < 0.05; **p < 0.01; ***p < 0.005. Bars show mean ± SEM, and circles represent individual donors.

(E and F) Representative examples of the flow profiling of DC subsets in human spleen (n = 3), dermis (n = 3) (E) and BM (n = 13) (F), gated as in (A). Histograms show CD163 and BTLA expression on CD14$^+$ (orange), CD5$^+$ (red) and CD14$^-$CD5$^-$ (gray) CD1c$^+$ DCs.

(G) tSNE visualization of the expression of TFs and surface markers across HC PB and BM lineage(lin, CD3,19,20,56,161)$^-$HLA-DR$^+$ cells by CyTOF analysis. Black gates indicate the CD1c$^+$DC population distinct from CD88$^+$monocytes, CLEC9A$^+$cDC1 and CD303$^+$pDC. Red and orange gates indicate expression of lymphocyte- or monocyte-associated antigens, respectively.

(H) Hierarchical clustering of single-cell transcriptomes of mature DCs from BM using all protein-coding, non-cell-cycle genes. Marker genes were identified within SC3 with parameters p < 0.01, area under the receiver operating characteristic curve (AUROC) > 0.85; cluster 1, pDCs (*GZMB*, *JCHAIN*); cluster 2, monocytes (*S100A8*, *VCAN*); cluster 3, CD14$^+$ DC3s (*HLA-DPB1*); cluster 5, cDC1s (*CD59*). The top rows show fluorescence intensity of surface antigens (“Antigens”) from index-sorted cells, and “Phenotype” denotes their classification defined by surface markers.

See also Figure S1.

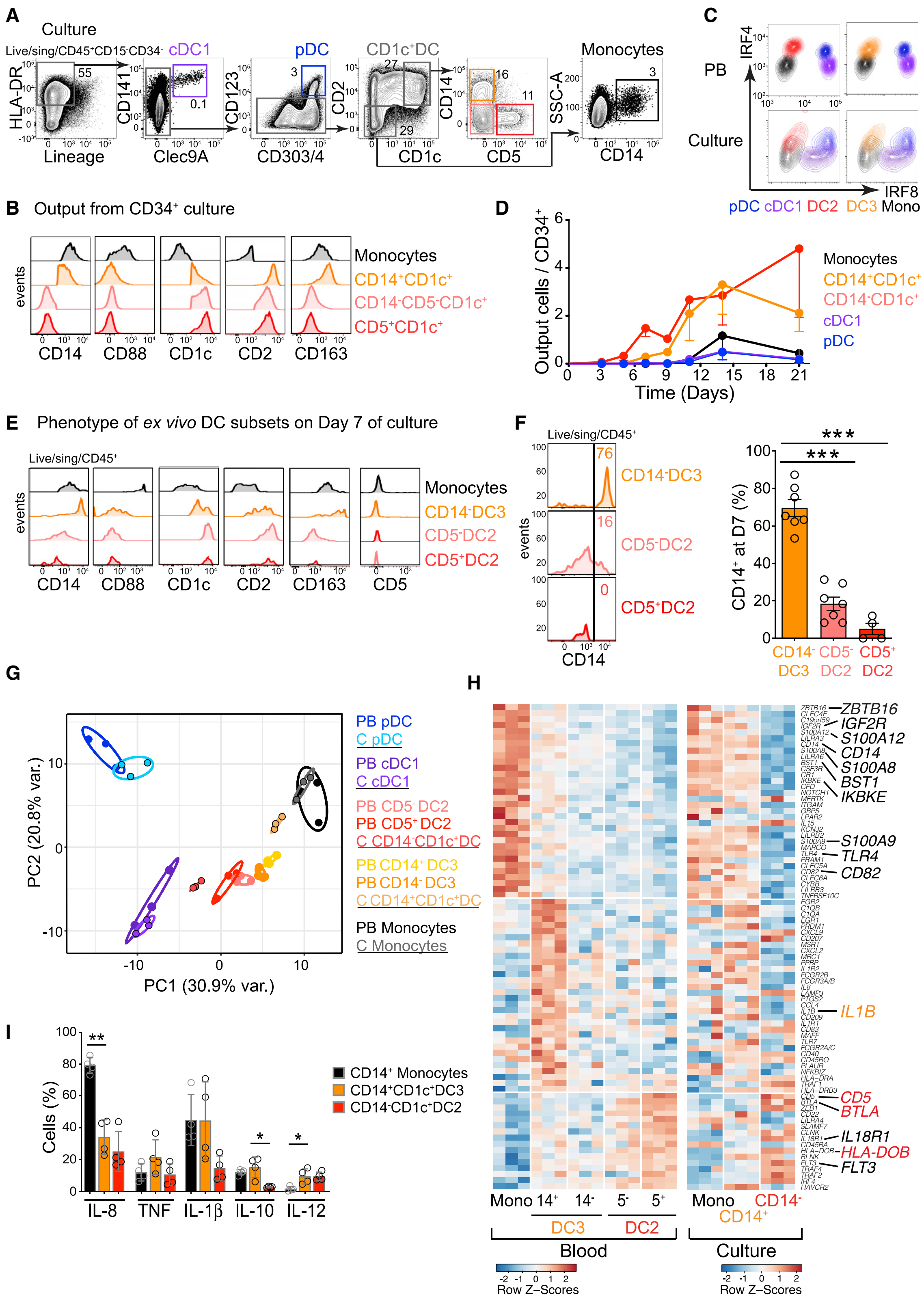

(legend on next page)

of CD1c, CD2, FcεR1A, and IRF4, distinct from CD88$^+$ monocytes and other DC subsets (Figures 1G, S1H, and S1I; Table S2). In both tissues, the CD5$^+$ pole was apposed to a small SIGLEC6^{+-} AXL$^+$ population (both BTLA$^+$ in PB), while the CD14$^+$ pole expressed monocyte-related antigens CD11b and CD36.

Index-sorted single-cell RNA sequencing (scRNA-seq) and unsupervised hierarchical clustering of mature DCs from HC BM confirmed that CD1c$^+$ DCs were heterogeneous and transcriptionally distinct from monocytes (Figures 1H and S1J; Table S3). CD14$^+$CD1c$^+$ DCs (cluster 3, high *HLA-DPB1*) clustered separately from CD14^{bright} monocytes (cluster 2, marked by *S100A8*, *VCAN*) but shared some monocyte-related transcripts. In contrast, clusters 6–8, encompassing CD5$^+$CD1c$^+$ DCs, shared features with cDC1s (cluster 5, *CD59*).

These experiments defined a set of antigens marking heterogeneity of CD1c$^+$ DCs in multiple tissues. Depending on the context, one or more antigens may be used to bisect the population into DCs enriched for lymphoid- (CD5 and BTLA) or monocyte-related (CD14 and CD163) markers. For consistency with recent literature, we will refer to CD163$^-$ (CD5$^+$ and CD5$^-$) cells as DC2s (BTLA$^+$ in PB) and CD163$^+$ (CD14$^+$ and CD14$^-$) cells as DC3s (BTLA$^-$ in PB) (Figure S1M). The presence of a discrete population of DC3s in BM is consistent with a direct hematopoietic origin rather peripheral conversion of monocytes.

CD14 Expression Distinguishes Heterogeneous CD1c$^+$ DC Subsets Generated *In Vitro*

The generation of CD1c$^+$ DCs subsets has not been previously demonstrated by *in vitro* culture. To probe this potential in human progenitor and precursor subsets we tested a system containing stem cell factor (SCF), FLT3 ligand (FL) and granulocyte-macrophage-colony-stimulating factor (GM-CSF) with *Csf1*$^{-/-}$ OP9 stromal cells to prevent overgrowth of monocytes (Nakano et al., 1994). It was possible to differentiate all primary DC subsets and some CD14$^+$ monocytes in this system (Figures 2A and S2A). The output was analyzed by at least two surface markers per subset. CD1c$^+$ DCs were distinguished from monocytes by their expression of CD1c and CD2 and lack of CD88 (Figures 2A and 2B). Within the CD1c$^+$DC compartment, CD163 was exclusively expressed by CD14$^+$ cells, while CD5$^+$ cells were contained within the CD14$^-$ population. In this system, CD14 expression defined populations corresponding to PB DC2s (CD14$^-$CD163$^-$) and DC3 (CD14$^+$CD163$^+$) (Figures 2A and 2B). Culture-derived DCs and monocytes retained appropriate expression of TFs IRF4 and IRF8 (Figure 2C).

Two observations suggested that CD1c$^+$ DC subsets were generated independently of monocytes and of each other. First, CD1c$^+$ DCs appeared early, ahead of monocytes (Figure 2D). Second, *ex vivo* PB CD1c$^+$ DC subsets and monocytes remained stable in culture for 7 days and did not interconvert (Figures 2E and S2B). Although some PB DCs gained CD14 expression *in vitro*, this was restricted to CD14$^-$ (CD163$^+$BTLA$^-$) DC3s (Figure 2F). Thus, *in vitro*, CD14 functions as a more inclusive marker for DC3 than in fresh PB, where it marks only the pole of this phenotype. There was some loss of CD5 expression on DC2s *in vitro*, but this did not hamper the separation of DCs and monocytes by CD88, CD1c, CD2, and CD163, which all remained stable (Figure 2E).

The identity of *in-vitro*-generated DC2s, DC3s, and monocytes generated in this system was validated by transcriptomic and functional analyses. Principal-component analysis (PCA) of NanoString gene expression data showed that *in-vitro*-generated CD14$^-$ DC2s and CD14$^+$ DC3s were appropriately polarized relative to cDC1s and monocytes (Figures 2G and S2C). Key signature transcripts of sorted PB DCs were also expressed in the corresponding cultured cells, including *BTLA*, *CD5*, and *HLA-DOB* in DC2s, *IL1B* in DC3s, and *ZBTB16* in monocytes (Figure 2H). Genes defining DC2s, DC3s, and monocytes (Villani et al., 2017) were appropriately enriched in culture-derived populations (Figures S2D and S2E), which also generated similar cytokine profiles to fresh PB DCs and monocytes on TLR stimulation (Figures 2I and S2F).

High IRF8 Expression Defines LMPP-Associated DC Progenitors

This *in vitro* culture system was used to map DC potential in sorted fractions of human BM. In describing immature cells,

Figure 2. CD14 Expression Distinguishes between CD1c$^+$DC Subsets Generated *In Vitro*

(A) Gating strategy used to identify DCs and monocytes generated from HC BM CD34$^+$ progenitors at day 21 (D21) of culture on OP9 in the presence of SCF, FL, and GM-CSF. A minimum of two antigens was used to define the following populations: CD141$^+$CLEC9A$^+$ cDC1s, CD123$^+$CD303$^+$CD304$^+$ pDCs, CD2$^+$CD1c$^+$ DCs encompassing CD14$^+$ and CD5$^+$ populations, and CD14$^+$CD1c$^-$CD2$^-$ monocytes.

(B) Flow analysis of the expression of population-specific markers across *in vitro*-generated monocytes (black), CD14$^+$ (orange), CD5$^+$ (red), or CD5$^-$ (pink) CD14$^-$CD1c$^+$ DCs as defined in (A).

(C) Intracellular flow evaluation of the expression of IRF4 and IRF8 by PB and culture-derived monocytes and DCs, gated as shown in Figure 1A and (A), respectively.

(D) Kinetics of DC culture output over 21 days plotted as the number of DCs or monocytes generated per CD34$^+$ progenitor. n = 6 donors with minimum n = 3 at each time point. Dots and bars show mean and SEM.

(E) Flow analysis of the expression of population-specific markers by FACS-purified PB monocytes and CD1c$^+$ subsets at day 7 of culture.

(F) Flow analysis of CD14 expression by FACS-purified PB CD1c$^+$subsets at day 7 of culture. Histogram shows a representative example from n = 7 (CD14$^-$ DC3 and CD5$^-$ DC2) or n = 5 (CD5$^+$ DC2) HC donors, summarized in the graph. Bars represent mean ± SEM. Circles represent individual donors. ***$p < 0.005$ by paired two-tailed t test.

(G) PCA of NanoString gene expression of FACS-purified PB DCs (“PB”) (n = 3) and DCs derived from BM CD34$^+$ progenitors at D21 of culture (“C”; black outline) (n = 3) after removal of a “culture signature” generated by pairwise comparison of all PB versus all culture-generated cells.

(H) Heatmap of *Z* scores of differentially expressed signature genes (NanoString) derived from pairwise comparisons of PB CD1c$^+$ DC subsets and monocytes, shown next to the *Z* scores of expression of the same genes by culture-derived CD14$^-$ and CD14$^+$ DCs and monocytes.

(I) Intracellular flow analysis of *in vitro* cytokine elaboration (percentage of positive cells) in response to TLR agonists, as described in Figure 1D, by CD14$^+$CD1c$^-$ monocytes (black bars), CD14$^+$ DC3s (orange), and CD14$^-$ DC2s (red) generated from n = 4 BM CD34$^+$ progenitors at day 21 of culture. p values from paired two-tailed t tests; *$p < 0.05$; **$p < 0.01$; ***$p < 0.005$. Bars show mean ± SEM.

See also Figure S2.

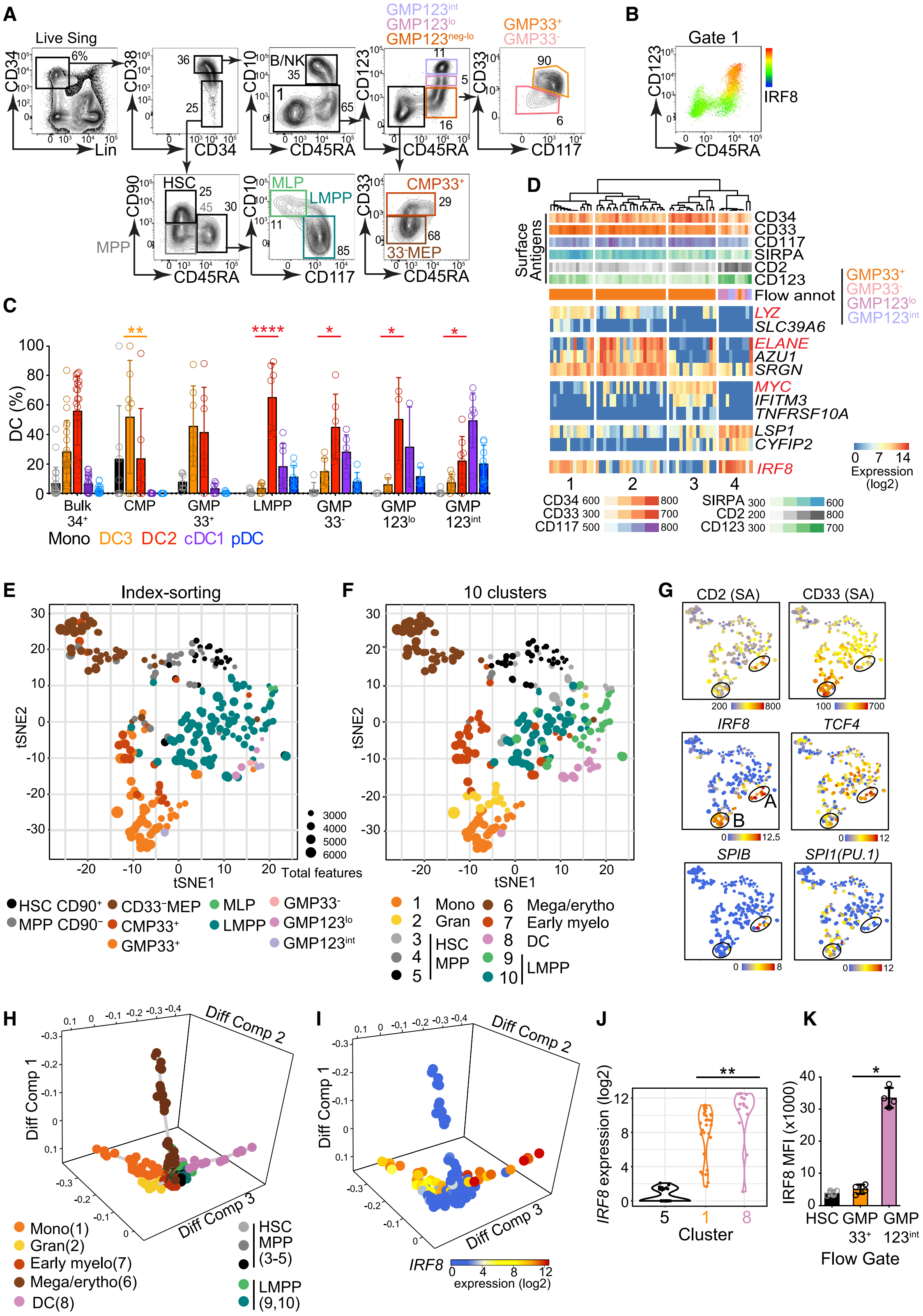

(legend on next page)

the terms "progenitor" and "precursor" refer specifically to $CD34^+$ and $CD34^{neg\text{-}int}$ populations, respectively (Table S4). Human DCs have previously been derived from classical myeloid progenitors (CMPs and GMPs), LMPPs, and $CD123^+$ fractions of the GMP, which were included here for comparison. HSCs and MPPs were identified in $CD38^{lo}$ gates (Figure 3A). $CD10^+$ MLP and $CD10^-CD117^+$LMPP fractions were selected from the $CD38^{lo}CD45RA^+$ population. From the $CD38^{hi}$ fraction were isolated $CD45RA^-$CMP megakaryocyte-erythroid progenitors (MEPs) and $CD45RA^+$ GMPs; $CD10^+$ B and natural killer (NK) cell progenitors (B/NKs) were excluded. Within the GMP gate, surface expression of CD123 correlated with intracellular expression of IRF8, so CD123 negative-low, low, and intermediate fractions were gated prospectively for differentiation studies (Figures 3B and S3A). Myeloid antigens CD33 and CD117 were expressed by a subset of $CD123^{neg\text{-}lo}$ GMPs ($GMP33^+$); $CD33^-CD117^-$ cells ($GMP33^-$) within this gate were analyzed separately (Figure 3A). CD33 was also expressed by cells in the $CD38^+CD45RA^-$ compartment, known to contain CMP and MEP populations. $CD33^+$ CMPs, with low expression of CD123, were sorted for comparison with $CD33^-$ cells, predicted to contain mostly MEPs (Figure S3B).

DC potential was found in CMPs, GMPs, and LMPPs (Figures 3C and S3C–S3E). pDCs, cDC1s, and DC2s mapped to LMPPs and $CD33^-$, $CD123^{lo}$, and $CD123^{int}$ fractions of GMPs. DC3 potential was principally found in the CMP and $CD123^{neg\text{-}lo}GMP33^+$ fractions (Figure 3C). Although there was incomplete dissociation of DC2 from DC3 potential using this apposed-gate strategy, the output ratio of DC2s to DC3s ranged widely, from 17.65 in LMPPs to 0.45 in $CD33^+$ CMPs (Figure S3D). The relatively higher production of pDCs, cDC1s, and DC2s in the $CD33^-$, $CD123^{lo}$, and $CD123^{int}$ GMP fractions was associated with increasing expression of IRF8 protein (Figures 3B, 3C, and S3A). In contrast, DC3 potential localized predominantly to $IRF8^{lo}$ progenitor fractions.

Transcriptional programming of the phenotype and culture potential seen in bulk populations was probed by index-sorted scRNA-seq of BM progenitors. Approximately equal numbers of $CD34^+$ progenitors (excluding $lineage^{lo}CD10^+$ B/NK progenitors) were sorted from the quadrants defined by a bivariate plot of CD45RA and CD38 (Figure S3F). scRNA-seq was performed with a modified SmartSeq2 protocol (Picelli et al., 2014). 262 out of 399 cells expressing 12,406 protein-coding genes passed quality control (QC) filters and cell-cycle-related transcripts were removed (STAR Methods; Table S3). The computational pipeline, including dimensionality reduction, hierarchical clustering, and trajectory analyses, was unbiased and driven solely by gene expression data. Clusters were then mapped to cell-surface phenotype from indexed flow data.

Hierarchical clustering of cells within the GMP compartment revealed close relationships among $CD33^-$, $CD123^{lo}$, and $CD123^{int}$ fractions of GMPs that formed a single $IRF8^{hi}$ cluster (cluster 4) distinct from $IRF8^{lo}$ $GMP33^+$ clusters associated with monocyte (cluster 1), granulocyte (cluster 2), and early myeloid gene expression (*LYZ*, *ELANE*, and *MYC*, respectively) (Paul et al., 2015; Wilson et al., 2004; Figures 3D and S3G–S3I).

Broadening the analysis to include scRNA-seq of HSCs, MPPs, MEPs, CMPs, LMPPs, and MLPs (Figure S3J), a single cluster contained cells with *in vitro* pDC, cDC1, and DC2 potential (cluster 8), marked by the expression of the DC-related genes *TCF4* and *RUNX* (Cisse et al., 2008; Satpathy et al., 2014). Cluster 8 was adjacent to LMPPs (clusters 9 and 10) but remote from CMPs and $GMP33^+$ (clusters 1 and 2) containing *in vitro* monocyte and DC3 potential.

Indexed phenotypes overlapped closely with cluster assignment visualized on t-distributed stochastic neighbor embedding (tSNE) plots (Figures 3E and 3F), although heterogeneity for DC-progenitor-related transcriptomes was revealed within phenotypic LMPPs. Phenotypic $CD33^+$ CMPs and GMPs also contained two clusters associated with monocytic or granulocytic gene expression, respectively (cluster 1, marked by *LYZ* and *CSF1R*; and cluster 2, marked by *ELANE*, *CALR*, and *FAM46A*) (Paul et al., 2015; Pellin et al., 2019; Figure S3J). The

Figure 3. High IRF8 Expression Defines LMPP-Associated DC Progenitors

(A) Flow gating strategy used to define and FACS-purify components of the $CD34^+$ $lin(CD3,14,16,19,20,7)^-$ compartment of human BM. HSC, hematopoietic stem cell; MPP, multipotent progenitor; MEP, megakaryocyte-erythroid progenitor; MLP, multilymphoid progenitor; LMPP, lymphoid-primed multipotent progenitor; CMP, common myeloid progenitor; GMP, granulocyte-macrophage progenitor.

(B) Heatmap of intracellular IRF8 protein expression across CMP and GMP as defined in (A) (gate 1).

(C) Monocyte and DC subset output from purified BM $CD34^+$ populations at day 14 of culture gated as in Figure 2A. Populations were quantified as percentage of the total cells captured by all DC and monocyte gates. Absolute output is shown in Figure S3C. Bulk $CD34^+$ (22 experiments from 13 donors: 22;13); CMP (7;5); $GMP33^+$ (7;6); LMPP (7;6); $GMP33^-$ (6;6); $GMP123^{lo}$ (3;3); $GMP123^{int}$ (8;7) (Table S4). Bars represent mean + SEM, and circles represent individual experiments. Significant differences in the proportional output of DC2s versus DC3s are indicated in red; *$p < 0.05$; **$p < 0.01$; ****$p = 0.0001$ (paired two-tailed t tests).

(D) Unsupervised hierarchical clustering of transcriptomes of single cells within the GMP index-sorting gate, using all protein-coding, non-cell-cycle genes, independent of surface antigen expression. Marker genes for four clusters identified within the single-cell consensus clustering 3 (SC3) tool ($p < 0.1$, AUROC > 0.75) and *IRF8* are displayed. The top rows show fluorescence intensity of surface antigens from index-sorted cells. Flow annotation ("Flow annot") denotes the classification of cells by their surface phenotype (Figures 3A and S3F).

(E–G) tSNE visualization of the first 10 principal components (25% of total variance) of the transcriptomes of 262 $CD34^+$ progenitor cells, independently of their surface phenotype. tSNE plots are shown annotated by (E), gate of origin from index-linked flow (Figure S3F), or (F), 10 clusters from hierarchical clustering (Figure S3J), Heatmaps (G) show flow surface antigen expression ("SA") and log2 expression of key DC TFs, *IRF8*, *TCF4*, *SPIB*, and *SPI1(PU.1)*, displayed across the tSNE plot (E and F). Black circles represent regions of high ("A") or low ("B") *IRF8* expression.

(H and I) Diffusion map using all protein-coding, non-cell-cycle genes. (H) The key specifies the designated cluster color, identity, and cluster number from Figure S3J. (I) *IRF8* expression. Diff Comp, diffusion component.

(J) Violin plot of differential *IRF8* expression (log2) in progenitor clusters 5 (HSCs and MPPs), 1 (monocyte enriched), and 8 (DC related). **$p = 0.001$ by Mann-Whitney U.

(K) Median fluorescence intensity (MFI) of intracellular IRF8 by flow analysis across gates identifying HC BM $CD34^+$ HSCs and $CD123^{neg\text{-}lo}$ $CD33^+$ and $CD123^{int}$ GMPs (n = 4) as defined in (A). *$p = 0.028$ by Mann-Whitney U.

See also Figure S3.

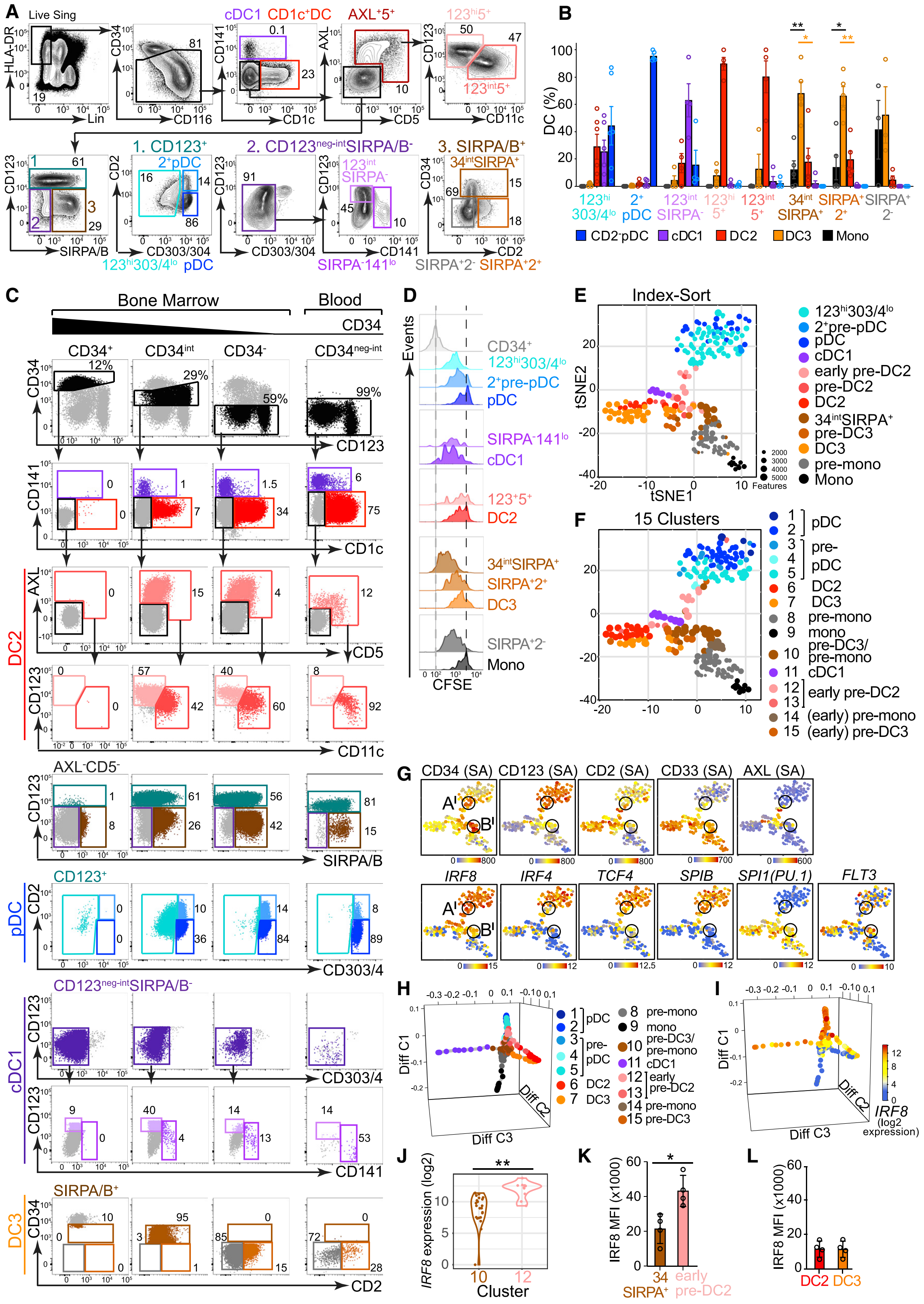

(legend on next page)

CellPress
OPEN ACCESS

majority of early progenitors did not express *IRF8*, but two signals were present: high expression associated with the DC cluster 8 and lower expression associated with the GMP33$^+$ monocytic cluster 1. The *IRF8^{hi}* region ("A"; Figure 3G) was also marked by high CD2, *TCF4*, and *SPIB*, and the *IRF8^{lo}* region ("B"; Figure 3G) expressed high CD33 and *SPI1 (PU.1)*. A and B are linked to DC precursor populations as described subsequently in Figure 4.

Diffusion mapping represented clusters 1 (monocyte enriched), 6 (MEPs), and 8 (DCs) as divergent trajectories (Figure 3H; Data S1). Clusters 9 and 10 (LMPPs) were located at the root of the DC trajectory and cluster 7 (early myeloid) at the root of the monocyte-gene enriched path. *IRF8* gene expression was statistically higher in the DC (cluster 8) compared to monocytic (cluster 1) trajectory (Figures 3I and 3J), as was the protein in corresponding indexed GMP populations (CD123^{int} versus CD33$^+$) (Figure 3K).

Taken together, the *in vitro* culture data, scRNA-seq analysis, and flow phenotypes are consistent with the transition of pDC, cDC1, and DC2 potential through LMPP phenotype space to CD33$^-$ and subsequently CD123$^+$ fractions of the GMP, where IRF8 is highly expressed. In contrast, DC3 potential segregates predominantly with monocyte development through a different region of IRF8^{lo} GMP parameter space marked by CD33 expression.

Two Trajectories of DC Development Connect the Progenitor Compartment with Mature DCs

The forward trajectories of DC potential were mapped within the CD34$^{neg\text{-}int}$ fraction of human BM. A gating strategy for these intermediate precursors was developed by iterative sorting and *in vitro* culture experiments (Figure 4A). After lineage$^+$ (lin), CD34$^+$, and mature DCs were removed, a population of AXL$^+$CD5$^+$ cells was identified, corresponding to pre-DC and "AS" DC populations previously described (See et al., 2017; Villani et al., 2017). AXL$^+$ cells expressed CD123, and variable CD11c inversely correlated with IRF8 expression (Figure S4A). Under our experimental conditions, CD1c$^-$ AXL$^+$CD5$^+$ cells contained only DC2 potential and were provisionally designated "early pre-DC2" (CD123^{hi}CD11c$^-$) and "pre-DC2" (CD123^{int} CD11c$^+$) (Figures 4B, S4B, and S4C). AXL$^-$CD5$^-$ cells were then gated on a bivariate plot of CD123 and SIRPA/B (Figure 4A). CD123$^+$ cells (gate 1, teal) contained CD2$^+$ and CD2$^-$ fractions of CD303^{hi}CD304^{hi} (CD303/4) pDCs, as previously reported (Matsui et al., 2009; Bryant et al., 2016). CD2$^+$ cells had precursor characteristics, with higher CD34 expression, more proliferative potential, and phenotypic conversion to CD2$^-$ pDCs *in vitro* (Figures S4D–S4F). Tri-lineage potential was observed in the CD123^{hi}CD303/4^{lo} gate (occupied by AXL$^+$CD5$^+$ cells, if not previously excluded) (Figure S4G).

The CD123$^{neg\text{-}int}$ SIRPA/B$^-$ population (gate 2, dark purple) contained CD34^{int}CD123^{int} cells enriched for cDC1 potential and adjacent to cells with low expression of the cDC1 marker CD141 (Figures 4A, 4B, and S4B). These CD34^{int}CD123^{int}SIRPA$^-$ "early pre-cDC1s" corresponded to CD34^{lo}CD100$^+$ cells with cDC1 potential detected previously (See et al., 2017; Villani et al., 2017), as confirmed by phenotypic and scRNA-seq analysis of PB, where these cells formed a distinct cluster marked by *NFIL3* (Figures 4C and S4L–S4R).

SIRPA/B$^+$ cells (Figure 4A, gate 3, dark brown) contained nearly all of the *in vitro* DC3 and monocyte potential. Among CD34$^-$ SIRPA/B$^+$ cells, CD2 expression enriched for DC3 potential (SIRPA$^+$2$^+$ "pre-DC3"). Under these experimental conditions, monocyte potential was relatively enriched in the CD2$^-$ "pre-monocyte" fraction (Figures 4B, S4B, and S4C).

This analysis demonstrated highly enriched single lineage DC potential within the CD34^{int} parameter space linking CD34$^+$ progenitors and CD34$^-$ mature DCs. This may be illustrated by applying the gating described in Figure 4A to CD34 high,

Figure 4. Two Trajectories of DC Development Connect the Progenitor Compartment with Mature DCs

(A) Flow gating strategy used to identify DCs and their precursors in BM, including CD141$^+$ cDC1s; CD1c$^+$ DCs; AXL$^+$CD5$^+$ cells composed of CD123^{hi}CD11c$^-$ (light pink) and CD123^{int}CD11c$^+$ (dark pink) fractions; CD2$^+$ (light blue) and CD2$^-$ (dark blue) pDCs; CD123$^+$CD303/4^{lo} cells (turquoise); SIRPA/B$^-$CD123^{int}CD141$^-$ (lightest purple) and CD141^{lo} (light purple) populations; and CD123$^-$SIRPA/B CD34^{int} (brown), CD34$^-$CD2$^+$ (dark orange), and CD34$^-$CD2$^-$ (gray) precursors.

(B) The output of *in vitro* culture of CD34^{int} DC precursors FACS-purified from BM using the gating strategy described in (A). Population-specific output is expressed as a proportion (%) of the total cells captured by all DC and monocyte gates. CD123^{hi}303/4^{lo} (six experiments from four donors; 6;4); CD2$^+$ pre-pDCs (5;3); CD123^{hi}5$^+$ (4;3); CD123^{int}5$^+$ (4;3); CD34^{int}CD123^{int} (4;4); CD34^{int}SIRPA$^+$ (5;5); SIRPA$^+$2$^+$ (4;4); SIRPA$^+$2$^-$ (4;4). Bars represent mean + SEM, and circles represent individual experiments. Significant differences in the proportional output of DC2s versus DC3s (red) or DC3s versus monocyte (black) are indicated: *$p < 0.05$; **$p < 0.01$ (paired, two-tailed t test).

(C) Flow gating strategy from (A) applied to lin$^-$HLA-DR$^+$ cells from HC BM fractionated by high, intermediate, and low CD34 expression, next to blood (columns) for comparison of antigen expression levels among progenitor, precursor, and mature populations. Individual DC lineages are ordered in rows.

(D) Proliferative potential of FACS-purified DC and DC precursors estimated by CFSE dilution (see STAR Methods). CD34$^+$progenitors and CD14$^+$ monocytes were included as positive and negative controls, respectively. The CFSE dilution histograms for each precursor are grouped and ordered according to their proposed position in the developmental trajectory for each DC lineage. Plots shown are representative of n = 3 experiments (summarized in Figure S4H).

(E and F) tSNE visualization of the first 20 principal components (explaining 35% total variance) of the transcriptomes of 244 single cells adaptively sampled from lin$^-$HLA-DR$^+$ CD34$^{neg\text{-}int}$ precursor and mature DC populations of BM. tSNE plots are annotated by the gate of origin from index-linked flow (E) or by 15 clusters generated from hierarchical clustering of all protein-coding non-cell-cycle genes (F), independently of surface phenotype (Figure S4K).

(G) Heatmaps showing the expression of key surface antigens (SAs) (index-linked flow) or log2 gene expression of TFs and FLT3 (scRNA-seq) across the tSNE plot in (E) and (F). Black circles represent regions of high or low *IRF8* expression, marked A^I or B^I, respectively. The differential expression patterns of these regions correspond to the patterns of regions "A" (IRF8^{hi}CD123^{int}GMP) and "B" (IRF8^{lo}GMP33$^+$) in Figures 3E–3G.

(H and I) Diffusion map generated with all protein-coding, non-cell-cycle genes to infer pseudo-temporal ordering of cells and reconstruct lineage branching. (H) Cells are colored according to the hierarchical clusters generated in Figure S4K. (I) *IRF8* expression (log2). Diff C, diffusion component.

(J) Violin plot of differential *IRF8* expression (log2) in clusters 10 (SIRPA$^+$34^{int}) and 12 (early pre-DC2). **$p < 0.001$ by Mann-Whitney U.

(K and L) MFI of intracellular IRF8 by flow analysis across gates identifying BM 34^{int}SIRPA$^+$ pre-DC3s and pre-mono and CD123^{hi}CD5$^+$ early pre-DC2s (K) and CD5$^+$ DC2s and CD5$^-$ DC3s (L) (n = 4). *p = 0.028 by Mann-Whitney U.

See also Figure S4.

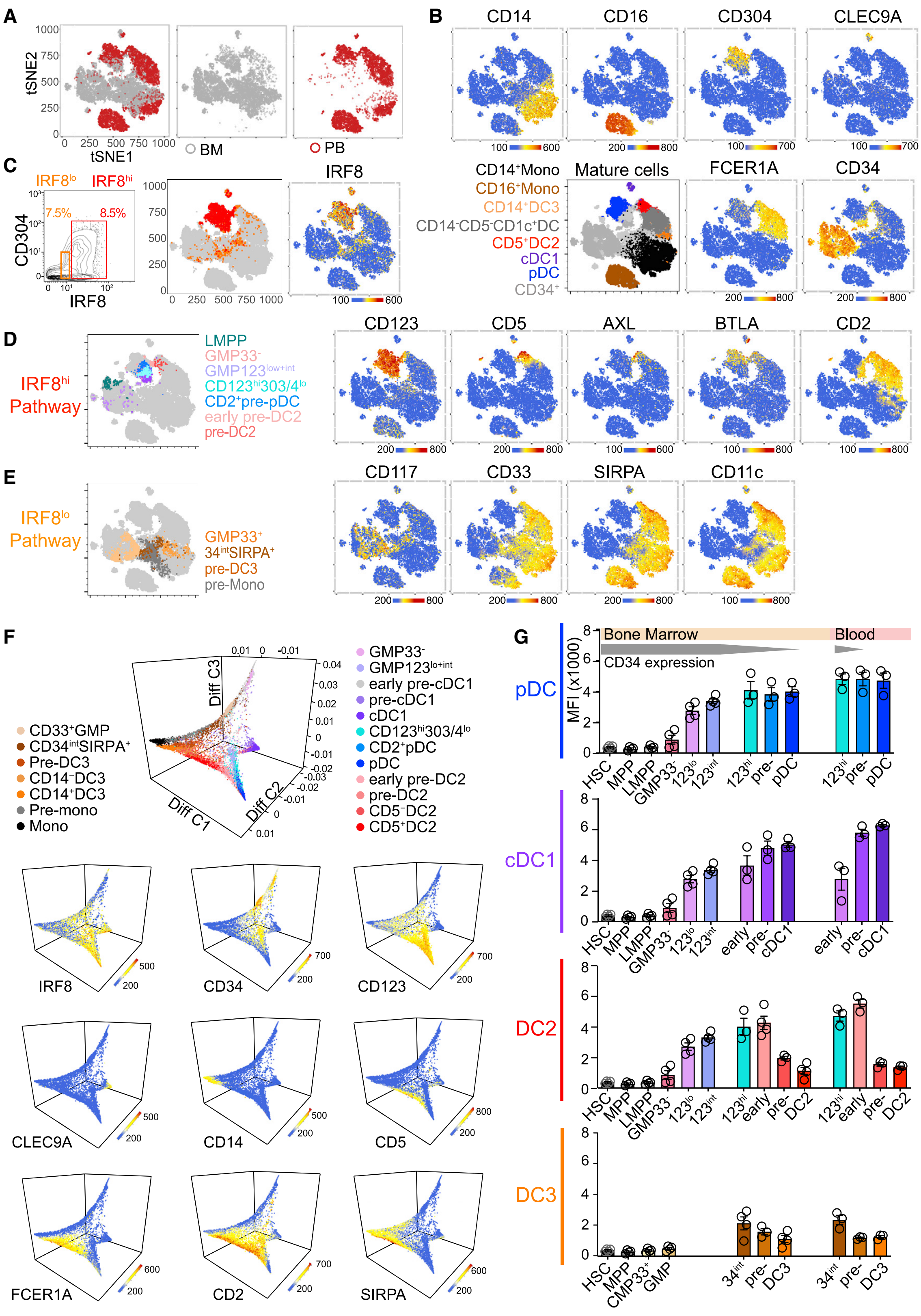

(legend on next page)

intermediate, and low fractions of lin^{-}HLA-DR^{+} BM and PB cells (Figure 4C). Carboxyfluorescein succinimidyl ester (CFSE) dilution assays showed a loss of proliferative potential in keeping with the proposed maturation trajectories (Figures 4D and S4H).

Seeking independent support for the proposed pathways, we performed scRNA-seq of CD34^{int} precursors and mature DCs from BM. Analysis used a computational pipeline driven only by gene expression data, independently indexed to the cell-surface phenotype used to define *in vitro* potential in the preceding experiments (Figures 4E–4J and S4I–S4K). 244 of 260 cells with expression of 12,137 protein-coding, non-cell-cycle genes passed QC (STAR Methods; Table S3).

Unsupervised hierarchical clustering generated clusters, annotated by their expression of known DC-subset-specific genes, that overlapped closely with indexed phenotypes (with the exception of CD123^{int}SIRPA^{-} cells, which were too rare to be identified discretely; Figures 4E, 4F, and S4I–S4K).

In tSNE visualization, DC2s and DC3s lay in adjacent halves of the CD1c^{+} DC population. DC2s (cluster 6), in the top half, were connected with cDC1s (cluster 11), AXL^{+} cells (cluster 12, expressing *SIGLEC6*), and pre-pDCs (clusters 3–5, expressing pDC genes *JCHAIN* and *MZB1*). DC3 (cluster 7, *CD14* and *VCAN*), in the lower half, were adjacent to CD34^{int} SIRPA^{+} cells (cluster 15, *VCAN*) and pre-monocytes (clusters 8 and 14, *MPO* and *AZU1*).

Two regions of the tSNE plot retained intermediate CD34 expression, marking immature precursor populations (A^{I} and B^{I},; Figure 4G). Their phenotypes, TF expression, and *in vitro* potentials corresponded very closely to the *IRF8^{hi}* and *IRF8^{lo}* regions identified in the progenitor analysis (A and B, respectively; Figure 3G). Specifically, A and A^{I} shared high *IRF8*, CD123, *TCF4*, and *SPIB* expression and gave rise to pDCs, cDC1s, and DC2 *in vitro*, while B and B^{I} expressed low amounts of *IRF8* but high CD33 and *SPI1(PU.1)* and generated predominantly DC3s and monocytes in culture. *FLT3* was expressed in all DC precursors, including SIRPA^{+}2^{+} pre-DC3, but not SIRPA^{+}2^{-}, pre-monocytes (Figure 4G).

Diffusion mapping defined distinct trajectories for cDC1s (cluster 11), pDCs (clusters 1–5), DC2s and DC3s (clusters 6 and 7), and monocytes (clusters 8 and 9; Figure 4H; Data S2). The DC2 trajectory originated in the CD123^{+}CD11c^{-}CD5^{+} early pre-DC2 population (clusters 12 and 13), adjacent to pDC origin, distinct from the origin of DC3 in CD34^{int}SIRPA^{+} pre-DC3 (clusters 10, 14, and 15), close to monocyte origin. IRF8 transcription and protein expression were higher in the DC2 trajectory than in DC3 (Figures 4J and 4K). As expected, IRF8 protein was low in both mature DC2s and DC3s (Figure 4L).

PB CD123^{int} precursors were similar to those isolated from BM with respect to scRNA-seq profiles and *in vitro* culture potential. IRF8^{hi}CD123^{+}CD2^{+}AXL^{+}CD5^{+} precursors, previously described as pre-DC (See) and AS DC (Villani), generated only DC2s (Figures S4L–S4R).

Differential IRF8 Expression Defines the Two Pathways of DC Development

Having identified the trajectories and key antigens mapping DC differentiation in BM and PB, we sought to integrate progenitors, precursors, and mature cells using an independent method. We used a CyTOF panel including progenitor markers (CD34 and CD117), intracellular TFs (IRF4 and IRF8), early DC lineage markers (AXL, SIGLEC6, CD123, CD2, CD33, and SIRPA), and mature DC and monocyte antigens (Figures 5A–5F and S5A–S5E; Table S2) to simultaneously analyze cells from BM and PB. Using tSNE dimension reduction, PB cells containing pDCs, cDC1s, CD1c^{+} DCs, and classical and nonclassical monocytes were located peripherally to progenitors and precursors present in BM. Populations were identified by key antigen expression or back-gating of sequentially gated populations (Figures 5A, 5B, S5B, and S5C). As previously shown, CD1c^{+} DCs, including the DC3 portion, were distinct from classical monocytes.

Superimposition of IRF8^{hi} (red) and IRF8^{lo} (orange) thresholds, from a bivariate plot of IRF8 and CD304, revealed distinct nonoverlapping regions of the tSNE plot (Figure 5C). IRF8^{hi} regions contained progenitors and precursors associated with pDC, cDC1, and DC2 lineages in the preceding analyses and AXL^{+}SIGLEC6^{+} (IRF8^{hi} CD123^{+}) pre-DCs, present in PB and BM, connected pDCs and cDC1s with the CD5^{+} BTLA^{+} pole of CD1c^{+} DCs (Figures 5B–5D and S5B–S5D).

In contrast, progenitors and precursors with monocyte or DC3 potential (mapped by CD117, CD33, SIRPA, and CD11c) segregated with low IRF8 expression and joined the CD1c^{+} DC cluster at a point discrete from AXL^{+}SIGLEC6^{+} cells (Figures 5E, S5B, and S5C). CD34^{int} expression was observed at both IRF8^{hi} and IRF8^{lo} contact points with the CD1c^{+} DC population (Figure 5B). As previously demonstrated (Figures 2C and 4L), IRF8 was not expressed in mature CD1c^{+} DCs.

Figure 5. Differential IRF8 Expression Defines the Two Trajectories of DC Development

(A–E) CyTOF analysis of FACS-purified CD45^{+}lin(CD3,19,20,56,161)$^{-}$ PB and BM progenitors, precursors, and mature DCs and monocytes using a panel of 33 surface antigens and two intracellular stains (IRF4 and IRF8). (A) tSNE visualization of lin^{-}HLA-DR^{+} cells, down-sampled to select 75,000 cells (20,000 CD11b^{+}CD14^{+} monocytes, 4,000 CD11b^{+}CD16^{+} monocytes, and 50,000 non-monocyte cells). PB (red) and BM (gray) cells were distinguished by differential CD45^{+} conjugate staining and displayed across tSNE space. (B) Heatmap of DC or monocyte-subset-specific antigens displayed on tSNE plots as in (A) (blue-yellow-red scales represent channel values). “Mature cells” plot shows the location of DC and monocyte subsets and CD34^{+} progenitors, identified by back-gating from bivariate plots (Figures S5B–S5D). (C) The location in tSNE space of IRF8^{hi} (red) and IRF8^{lo} (orange) expressing cells identified by (1) standard gating on a bivariate plot of IRF8 versus CD304 and superimposition of these gated cells on tSNE space and (2) a heatmap of IRF8 expression across all cells. (D and E) Location in tSNE space of progenitors and precursors with pDC, cDC1, or DC2 (D) and DC3 or monocyte (E) potential as defined by previous experiments, identified by back-gating from bivariate plots (Figures S5B and S5C), and heatmaps of associated antigens.

(F) Diffusion map generated with 14,000 cells including GMPs, precursor and mature DCs, and monocytes. Populations were identified and color-coded according to Figures 3A (progenitors) and 4A (precursors, DCs, and monocytes), applied to CyTOF data as shown in Figures S5B and S5C. Heatmaps show the expression (log2) of IRF8 and key antigens superimposed across the diffusion map trajectories. See also Figure S5E. Diff C, diffusion component.

(G) Histograms summarizing IRF8 protein expression by flow cytometry (MFI) in progenitors, precursors, and mature cells of pDC, cDC1, DC2, and DC3 lineages from BM and PB. Bars show mean ± SEM. Circles show individual donors (BM progenitors, n = 4; BM and PB precursors and mature DCs, n = 3).

See also Figure S5.

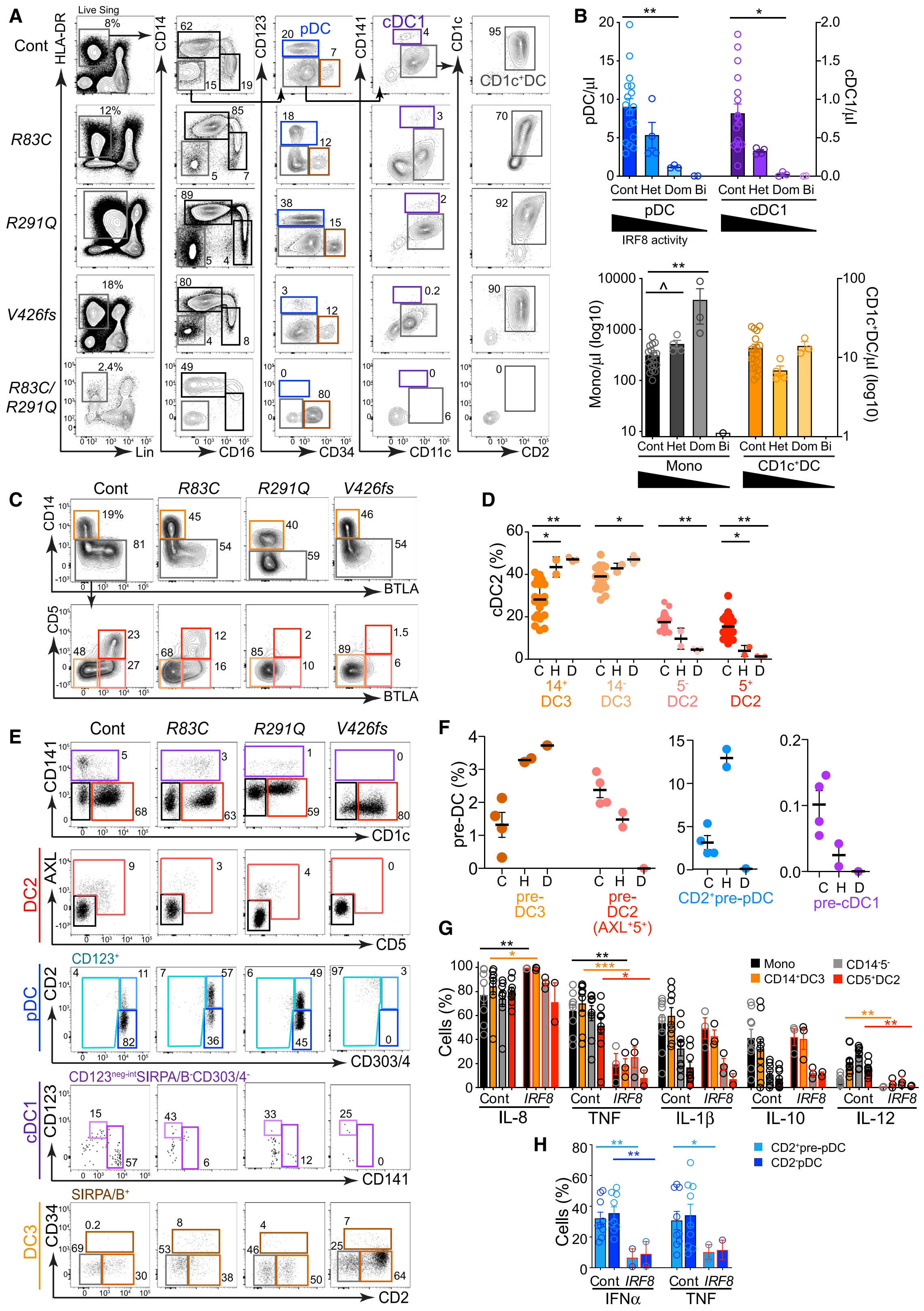

(legend on next page)

CellPress
OPEN ACCESS

Diffusion mapping of 14,000 randomly sampled GMP, precursor, and mature populations from the experiment produced a result coherent with the preceding *in vitro* culture outputs and with trajectories driven by scRNA-seq data (Figure 5F; Data S3). The analysis generated a tetrahedron in Euclidean space with progenitors at the apex and monocytes, pDCs, and cDC1s at the vertices. DC3 and DC3 precursors lay close to the monocyte pathway linked to the GMP33^{+} by IRF8^{lo} populations (brown and rust). DC2 precursors descended closer to pDCs through CD123$^{lo\text{-}int}$ IRF8^{hi} GMPs (lilac). As expected, mature DC2s and DC3s lay between monocytes and DCs along diffusion component 1. Both mature populations expressed FCER1, SIRPA, and CD2, but there was mutually exclusive expression of CD14 and CD5. These pathways could be visualized on standard bivariate plots (Figures S5F and S5G). Intracellular flow was used to pinpoint the stage-specific expression of IRF8 protein along each pathway of DC development (Figure 5G).

IRF8^{hi} and IRF8^{lo} Pathways Are Differentially Compromised in IRF8 Deficiency

We analyzed nine individuals from three kindreds with *IRF8* mutation to define the dependence of each pathway of DC development on IRF8 activity. Bi-allelic *IRF8*$^{K108E/K108E}$ and *IRF8*$^{R83C/R291Q}$ patients were compared with their minimally affected heterozygous parents (Hambleton et al., 2011; Bigley et al., 2018) and three individuals from a third kindred with an autosomal-dominant phenotype due to dominant-negative *IRF8*^{V426fs} (unpublished data).

Heterozygous parents of the child carrying *IRF8*$^{R83C/R291Q}$ had 20%–50% loss of pDCs, cDC1s, and CD1c^{+} DCs (Figures 6A and 6B). In retrospect this matched the phenotype of heterozygous *IRF8*^{K108E} (Hambleton et al., 2011) and is in keeping with a gene-dosage effect of *IRF8* on DC development. *IRF8*^{V426fs} mutation produced an intermediate cellular phenotype congruent with clinical manifestations that were more severe than heterozygotes (*IRF8*^{R83C} and *IRF8*^{R291Q}) but less than bi-allelic *IRF8* deficiency (*IRF8*$^{R83C/R291Q}$) (Figures 6A, 6B, and S6A). Both pDCs and cDC1s were depleted with *V426fs* mutation. A trend toward monocytosis in the asymptomatic heterozygotes (*IRF8*^{R83C} and *IRF8*^{R291Q}) became significant in *IRF8*^{V426fs}.

CD1c^{+} DCs presented a paradox; although *IRF8*^{R83C} and *IRF8*^{R291Q} were lower than controls, lower IRF8 activity in *IRF8*^{V426fs} restored CD1c^{+} DCs (Figure 6C). The proportion of DC2s and DC3s accounted for this anomaly; DC2s, pDCs, and cDC1s decreased with loss of IRF8, but this was compensated for by an increase in DC3 such that CD1c^{+} DC population of *IRF8*^{V426fs} consisted almost entirely of DC3s (Figure 6D). Parallel effects occurred in pre-DCs as defined by the preceding analysis; AXL^{+}CD5^{+} pre-DC2s were lost in parallel with DC2s, but SIRPA/B^{+}CD2^{+} pre-DC3s increased proportionately with DC3s and monocytes (Figures 6E and 6F). In heterozygotes with sufficient cells to analyze, loss of IRF8 reduced tumor necrosis factor (TNF) and IL-12 in DC2s and DC3s while IFN-α and TNF production was decreased in CD2^{+}pre-pDCs and pDCs (Figures 6G, 6H, S6B, and S6C).

IRF8 Deficiency Causes Dose-Dependent Blockade of the IRF8^{hi} Pathway

Seeking further evidence of a dissociation between IRF8^{hi} and IRF8^{lo} DC pathways, we probed the progenitor and DC precursor compartments of BM for dose-dependent effects of *IRF8*^{V426fs} and *IRF8*$^{R83C/R291Q}$ (Figures 7A, 7B, and S7A). We inferred the point of developmental blockade by expansion of a proximal population coupled with loss of cells immediately distal to it (red arrows, Figure 7C). In the IRF8^{hi} pathway, defects occurred increasingly more proximally; heterozygous mutations affected the precursors, and dominant-negative and bi-allelic mutations impacted the proportions of CD123^{lo}, CD123^{int}, and CD33^{-}CD117^{-} GMPs in a stepwise fashion. In contrast, the IRF8^{lo} pathway leading to DC3s was only sensitive to complete bi-allelic loss of *IRF8,* late in the precursor compartment.

In vitro differentiation and transcriptomic analyses provided evidence of two pathways of DC development, distinguished by their high or low IRF8 expression, giving rise to cDC1s, pDCs, and DC2s or DC3s and monocytes, respectively.

In partial IRF8 deficiency, the incremental loss of subsets derived from the IRF8^{hi} trajectory, associated with preservation or expansion of IRF8^{lo} populations, demonstrates the differential IRF8 requirement of these pathways in the intact human.

DISCUSSION

CD1c^{+} DCs are heterogeneous by phenotype, gene expression, and function. DC2s are enriched for classical cDC1-related

Figure 6. IRF8^{hi} and IRF8^{lo} Pathways Are Differentially Compromised in IRF8 Deficiency

(A) PB flow analysis of monocytes and DCs in subjects carrying heterozygous *IRF8*^{R83C} or *IRF8*^{R291Q} mutation (Het), their child carrying *IRF8*$^{R83C/R291Q}$ (Bi), and a carrier of dominant-negative heterozygous mutation *IRF8*^{V426fs} (Dom) compared with HC (Cont).

(B) Trucount quantification of PB DCs and monocytes in subjects carrying *IRF8* mutations (gating shown in Figure S6A; Hambleton et al., 2011; Bigley et al., 2018)). Cont, n = 25; Het, n = 4 (*IRF8*^{R83C}, *IRF8*^{R291Q}, and two subjects carrying *IRF8*^{K108E}); Dom, n = 3 (*IRF8*^{V426fs}); Bi, n = 2 (*IRF8*$^{R83C/R291Q}$ and *IRF8*$^{K108E/K108E}$).

(C) Flow cytometry phenotyping of CD1c^{+} DC subsets derived from the CD1c^{+}CD2^{+} gate (gray) in (A) to identify CD14^{+} DC3s (orange), CD14^{-}BTLA^{-} DC3s (light orange), CD5^{-}BTLA^{+} DC2s (light red), and CD5^{+}BTLA^{+} DC2s (red).

(D) Proportion of CD1c^{+} DC subsets (gated as in C, from the individuals represented in B). C, control; H, heterozygous parents; D, dominant-negative heterozygotes (*IRF8*^{V426fs}).

(E) Flow analysis of DC and monocyte precursors in PB of subjects carrying *IRF8* mutations as shown, gated as in Figure 4C.

(F) Proportion of DC and monocyte precursors out of all pre-DCs in PB of subjects carrying *IRF8* mutations, gated as in (E). C, control; H, heterozygous; D, *IRF8*^{V426fs}

(G and H) Intracellular flow analysis of *in vitro* cytokine elaboration (percentage of positive cells) by CD14^{+} monocytes (black), CD14^{+} DC3s (orange), CD14^{-}CD5^{-}CD1c^{+} DCs (gray), and CD5^{+} DC2s (red) (G) and CD2^{+} pre-pDCs and pDCs from HC (n = 8) and subjects carrying heterozygous *IRF8*^{R83C}, IRF8^{R291Q} (mean of technical duplicates) or IRF8^{V426fs} (IRF8, red-outlined bars) (H). See also STAR Methods and Figure 1H.

Bars show mean ± SEM, and circles represent individual subjects. *p < 0.05; **p < 0.01; ***p < 0.001; ^p = 0.053, Mann-Whitney U. See also Figure S6.

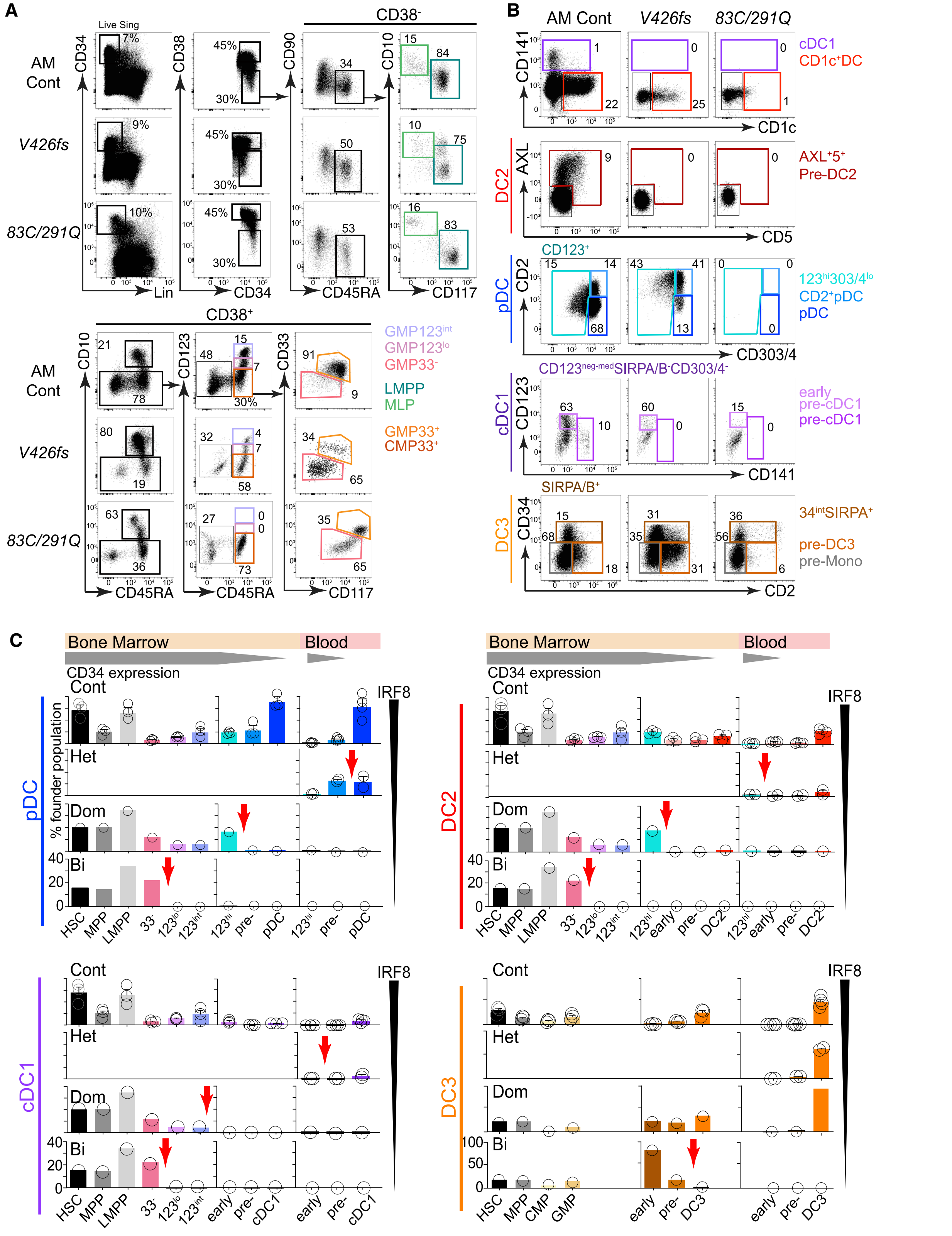

(legend on next page)

properties, while DC3s are closer to monocytes (Schrøder et al., 2016; Yin et al., 2017; Alcántara-Hernández et al., 2017; Korenfeld et al., 2017; Villani et al., 2017; Bourdely et al., 2020). Here, we have shown that this heterogeneity originated in two distinct pathways of hematopoiesis, with differential requirements for IRF8. Using *in vitro* differentiation assays tuned to distinguish between DC2 and DC3 outputs, we showed that their developmental potentials lie in mutually exclusive populations of progenitors and precursors. Two developmental trajectories were apparent from high-dimensional analysis of antigen expression, unbiased scRNA-seq, and diffusion mapping. Finally, a human *IRF8* allelic series revealed differential sensitivity of the two pathways to loss of IRF8 activity.

The DC2 IRF8^{hi} pathway followed a classical DC trajectory closely related to pDCs and cDC1s. Distinct DC2 potential was first evident in LMPPs, which were heterogeneous at single-cell resolution, and was traceable through CD123^{lo}CD33^{-} CD117^{-} GMPs. Progressive enrichment of IRF8^{hi} pathway DC potential was observed with increasing CD123 expression in the GMP compartment. The maximum expression of CD123 by GMPs was CD123^{int} relative to CD123^{hi} expression in PB. The CD123^{+} tip of the GMP generated the CD123^{hi} cells found among CD34^{int} BM and PB cells, containing restricted pDC and DC2 potential. CD5 and transient expression of AXL and SIGLEC6 separated these two components. As CD123 expression was lost, the characteristic CD1c^{+} DC markers CD11c and CD1c were progressively acquired, IRF8 was downregulated, and IRF4 dominance was acquired. pDCs developed along a CD123^{hi} trajectory marked by continued high expression of IRF8 and IRF4, acquiring CD303/4 as CD34 expression was lost. The cDC1 trajectory, characterized by the highest IRF8 expression, took a variant route from the GMP. The most enriched flux appeared to leave the CD123^{int} tip of the GMP as a small population of cells retaining residual proliferative capacity and CD34^{int} expression. These became CD123^{int}CD11c^{lo} PB cells that subsequently acquired CD141, CLEC9A, and a state of high IRF8 unopposed by IRF4. This population was previously detected among multiply lineage-negative cells by co-expression of CD100 and CD34 but not connected to the cDC1 trajectory (Villani et al., 2017). Pre-DC2s and pre-cDC1s were much more obvious when lin^{-}HLA-DR^{+} BM cells were fractionated by decrements of CD34 expression.

The DC3 pathway was related to monocyte development, marked by low expression of IRF8. However, DC3s are not "monocyte derived" for the following reasons: (1) they were observed fully formed in the BM compartment, (2) their potential was highest in a phenotypically defined precursor group independent of monocytes (SIRPA/B^{+}CD2^{+}), (3) they appeared in progenitor cell culture earlier than monocytes, and (4) they developed under conditions that prevent monocyte differentiation into DC3.

Enrichment for DC2 and DC3 potential within discrete progenitor populations is congruent with lineage-primed descriptions of hematopoiesis, in which DC potential is specified at an early stage (Schlitzer et al., 2015; Lee et al., 2017). Specifically, we observed DC2 trajectory transcriptomes in subsets of LMPP and CD123$^{neg\text{-}lo}$CD33^{-} GMP and DC3 and monocyte-related transcriptomes, distinct from granulocyte precursors, in the CD123$^{neg\text{-}lo}$CD33^{+} GMP.

The developmental trajectories of pDCs, cDC1s, and CD1c^{+} DCs (DC2 and DC3 together) have been previously mapped at single-cell resolution by Lee et al. (2017), who demonstrated that the CD123^{int} GMP contains only unipotent cell potential (pDCs, cDC1s, or CD1c^{+} DCs) and that the CD123$^{neg\text{-}lo}$ GMP contains cells with dual cDC1 and CD1c^{+} DC or CD1c^{+} DC and mono- potential. Herein, we have shown that the cDC1 and CD1c^{+}DC potential of CD123$^{lo\text{-}int}$GMP gave rise almost exclusively to DC2 while the greatest enrichment of monocyte and DC3 potential was found in the CD123$^{neg\text{-}lo}$CD33^{+} subset of GMP. The use of single cultures would not alter the interpretation of our data that the outputs of these two GMP fractions are distinct. Where single-cell cultures will be essential, in future experiments, is to explore lineage-priming at the origin of the DC2 and DC3 pathways in primitive HSC or MPP populations. It also remains to be determined whether alternative exogenous factors can modulate the potentials demonstrated *in vitro*.

Many reports have highlighted dose-dependent effects of *Irf8* on murine DC development (Tailor et al., 2008; Grajales-Reyes et al., 2015; Sichien et al., 2016). Collectively, these show that cDC1s are most sensitive to Irf8 loss, requiring high expression at the terminal stages of differentiation. Murine pDCs survive Irf8 deficiency but are functionally altered, while the equivalents of CD1c^{+} DCs (cDC2s) are preserved. Monocytes are not affected until *Irf8* is ablated, when they are blocked at the GMP stage.

In humans, reduced IRF8 activity in asymptomatic heterozygotes and individuals with the dominant-negative *V426fs* allele was associated with reduction or depletion of all of the IRF8^{hi} pathway classical DCs (pDCs, cDC1s, and DC2s). In contrast, IRF8^{lo} DC3s and monocytes were maintained or even expanded until IRF8 activity was completely absent in the patients carrying bi-allelic mutations, resulting in the loss of all DCs and monocytes. In contrast to mouse, human pDCs were almost as sensitive as cDC1s to loss of IRF8. However, in asymptomatic heterozygotes, where there was only partial depletion, we observed an increased proportion of CD2^{+} pDCs and functional deficits in IFN-α and TNF production similar to that reported in mice (Sichien et al., 2016). When CD1c^{+}DCs are considered as a single

Figure 7. IRF8 Deficiency Causes Dose-Dependent Blockade of the IRF8^{hi} Pathway

(A and B) Flow cytometry analysis of BM CD34^{+} progenitors (A) and DC and monocyte precursors (B) from the subjects carrying dominant-negative *IRF8^{V426fs}* and bi-allelic *IRF8* mutations and an age-matched control (AM Cont). BM was not available from healthy heterozygotes *IRF8^{R83C}* and *IRF8^{R291Q}*. Gating and color coding as in Figures 3A and 4C.

(C) The relative proportions of progenitors and precursors in BM and PB from controls (n = 3 BM, n = 4 PB) and individuals carrying heterozygous *IRF8^{R83C}* and *IRF8^{R291Q}* (PB, Het), *IRF8^{V426fs}* (Dom), or *IRF8$^{R83C/R291Q}$ (Bi)* to pinpoint the block associated with progressive loss of IRF8 activity for each DC lineage. CD34^{+} populations were expressed as a proportion of total gated CD34^{+} cells. Precursor and mature DC populations were expressed as a proportion of the total number of gated CD34$^{neg\text{-}int}$ cells. Likely points of blockade are indicated by red arrows.

See also Figure S7.

entity, the total population appeared to remain intact in the context of partial depletion of IRF8, as observed in asymptomatic heterozygotes and individuals with the dominant-negative *V426fs* allele. However, separation of CD1c$^+$ DCs into IRF8^{hi} pathway DC2 and IRF8^{lo} pathway DC3 components showed that DC3s populated the parameter space left empty by missing DC2s. A dominant-negative allele of *IRF8* has been previously reported with the substitution *T80A* (Hambleton et al., 2011). Although this was originally thought to have intact cDC1s and a defect of CD1c$^+$ DCs, improved analysis using CLEC9A recently confirmed that cDC1s are indeed selectively depleted in these heterozygous individuals (Kong et al., 2018). Lower expression of CD1c is also possibly explained by replacement of DC2 by DC3. Thus, all *IRF8*-mutated individuals now show congruous cellular phenotypes.

These observations highlight the phenomenon that cellular deficiency due to hematopoietic TF mutation often results in expansion of related lineages, owing to the unopposed action of competing TFs. We have previously described the marked neutrophilia accompanying bi-allelic *IRF8* deficiency (Hambleton et al., 2011; Bigley et al., 2018), probably due to the action of unopposed CEBPα (Becker et al., 2012; Kurotaki et al., 2014). In this study, we have reported monocytosis and expansion of DC3 in *IRF8* heterozygous states potentially related to excessive SPI1 (PU.1) activity when IRF8 is partially absent (Lee et al., 2017; Giladi et al., 2018).

Our results reinforce the view that gene-dosage effects and autosomal-dominant patterns of inheritance often occur in TFs controlled by super-enhancers (Afzali et al., 2017). Experimentally, this proved critical in analyzing the differential requirement for IRF8 between two DC pathways. Through this analysis, we have refined the concept of "classical DCs" as cDC1 and DC2 dependent on the IRF8^{hi} pathway and distinct from DC3 and monocyte development by the IRF8^{lo} pathway. Although we do not have a biochemical means of assessing total IRF8 "activity" in the intact hematopoietic system and made inferences from the severity of clinical phenotypes, all the mutations described are deleterious in reporter assays (Bigley et al., 2018).

In conclusion, our data support a model whereby CD1c$^+$ DC heterogeneity arises from distinct lineage trajectories within the CD34$^+$ progenitor compartment, progressing along pathways distinguished by high or low IRF8 expression, comprised of phenotypically identifiable precursors. Distal convergence to a CD1c$^+$ DC phenotype results in the observed phenotypic, transcriptomic, and functional heterogeneity of CD1c$^+$ DCs.

STAR★METHODS

Detailed methods are provided in the online version of this paper and include the following:

- KEY RESOURCES TABLE
- RESOURCE AVAILABILITY
  - Lead Contact
  - Materials Availability
  - Data and Code Availability
  - Subject Details
  - Patients and healthy donors
- METHOD DETAILS
  - Flow cytometry and cell sorting
  - *In vitro* generation of dendritic cells
  - Dendritic cell functional analysis
  - NanoString nCounter analysis
  - Single cell RNA sequencing
  - Alignment of reads to the human reference genome
  - Gene and cell filtering
  - Cell clustering
  - tSNE analysis
  - Diffusion maps and lineage tracing
  - Helios Mass Cytometer (CyTOF) analysis
  - tSNE analysis
  - Diffusion maps and lineage tracing
- QUANTIFICATION AND STATISTICAL ANALYSIS

SUPPLEMENTAL INFORMATION

Supplemental Information can be found online at https://doi.org/10.1016/j.immuni.2020.07.003.

ACKNOWLEDGMENTS

We gratefully thank healthy donors, patients and their families for their participation. We thank NIHR BioResource volunteers for their participation, and we gratefully acknowledge the NIHR Bioresource Centre, NHS Trusts, and staff for their contribution. We thank the National Institute for Health Research, NHS Blood and Transplant, and Health Data Research UK as part of the Digital Innovation Hub Programme. The views expressed are those of the author(s) and not necessarily those of the NHS, the NIHR or the Department of Health and Social Care. We also thank staff at the Newcastle University Flow Cytometry Core Facility (FCCF) for assistance with the generation of flow cytometry data, as well as Lorna Witty and James Docker from the Oxford Genomics Centre (Wellcome Centre for Human Genetics, Oxford, UK). This work was supported by grants from the Wellcome Trust (101155/Z/13/Z to V.B. and U.C.), CRUK (C30484/A21025 to M.C.), Newcastle University Single Cell Unit, and the NIHR Newcastle Biomedical Research Centre at Newcastle upon Tyne Hospitals.

AUTHOR CONTRIBUTIONS

Conceptualization, M.C. and V.B.; Methodology, U.C., A.R., S.P., K.G., P.M., S.M., D.D., G.H., A.F., B.C., R.Q., M.C., and V.B.; Software, A.R. and R.Q.; Formal Analysis, U.C., A.R., K.G., R.Q., and V.B.; Investigation, U.C., A.R., S.P., K.G., P.M., S.M., D.D., G.H., B.C., R.Q., G.D., and V.B.; Resources, S.H., R.H., H.L.A., J.E.D.T., G.D., M.C., and V.B.; Data Curation, U.C., A.R., S.P., D.D., and V.B.; Writing – Original Draft, U.C., A.R., M.C., and V.B.; Writing – Review & Editing, D.M., G.H., A.F., S.H., and G.D.; Visualization, U.C., A.R., M.C., and V.B.; Supervision, M.C. and V.B.; Funding Acquisition, M.C. and V.B.

DECLARATION OF INTERESTS

The authors declare no competing interests.

Received: March 26, 2019
Revised: December 27, 2019
Accepted: July 2, 2020
Published: July 30, 2020

REFERENCES

Afzali, B., Grönholm, J., Vandrovcova, J., O'Brien, C., Sun, H.W., Vanderleyden, I., Davis, F.P., Khoder, A., Zhang, Y., Hegazy, A.N., et al. (2017). BACH2 immunodeficiency illustrates an association between super-enhancers and haploinsufficiency. Nat. Immunol. *18*, 813–823.

CellPress
OPEN ACCESS

Alcántara-Hernández, M., Leylek, R., Wagar, L.E., Engleman, E.G., Keler, T., Marinkovich, M.P., Davis, M.M., Nolan, G.P., and Idoyaga, J. (2017). High-dimensional phenotypic mapping of human dendritic cells reveals interindividual variation and tissue specialization. Immunity *47*, 1037–1050.e6.

Anders, S., Pyl, P.T., and Huber, W. (2015). HTSeq—a Python framework to work with high-throughput sequencing data. Bioinformatics *31*, 166–169.

Angerer, P., Haghverdi, L., Büttner, M., Theis, F.J., Marr, C., and Buettner, F. (2016). destiny: diffusion maps for large-scale single-cell data in R. Bioinformatics *32*, 1241–1243.

Becker, A.M., Michael, D.G., Satpathy, A.T., Sciammas, R., Singh, H., and Bhattacharya, D. (2012). IRF-8 extinguishes neutrophil production and promotes dendritic cell lineage commitment in both myeloid and lymphoid mouse progenitors. Blood *119*, 2003–2012.

Bigley, V., Maisuria, S., Cytlak, U., Jardine, L., Care, M.A., Green, K., Gunawan, M., Milne, P., Dickinson, R., Wiscombe, S., et al. (2018). Biallelic interferon regulatory factor 8 mutation: A complex immunodeficiency syndrome with dendritic cell deficiency, monocytopenia, and immune dysregulation. J. Allergy Clin. Immunol. *141*, 2234–2248.

Bolger, A.M., Lohse, M., and Usadel, B. (2014). Trimmomatic: a flexible trimmer for Illumina sequence data. Bioinformatics *30*, 2114–2120.

Bourdely, P., Anselmi, G., Vaivode, K., Ramos, R.N., Missolo-Koussou, Y., Hidalgo, S., Tosselo, J., Nuñez, N., Richer, W., Vincent-Salomon, A., et al. (2020). Transcriptional and Functional Analysis of CD1c^{+}Human Dendritic Cells Identifies a CD163^{+}Subset Priming CD8^{+}CD103^{+}T Cells. Immunity *53*, https://doi.org/10.1016/j.immuni.2020.06.002.

Bryant, C., Fromm, P.D., Kupresanin, F., Clark, G., Lee, K., Clarke, C., Silveira, P.A., Suen, H., Brown, R., Newman, E., et al. (2016). A CD2 high-expressing stress-resistant human plasmacytoid dendritic-cell subset. Immunol. Cell Biol. *94*, 447–457.

Cisse, B., Caton, M.L., Lehner, M., Maeda, T., Scheu, S., Locksley, R., Holmberg, D., Zweier, C., den Hollander, N.S., Kant, S.G., et al. (2008). Transcription factor E2-2 is an essential and specific regulator of plasmacytoid dendritic cell development. Cell *135*, 37–48.

Collin, M., and Bigley, V. (2018). Human dendritic cell subsets: an update. Immunology *154*, 3–20.

Cytlak, U., Resteu, A., Bogaert, D., Kuehn, H.S., Altmann, T., Gennery, A., Jackson, G., Kumanovics, A., Voelkerding, K.V., Prader, S., et al. (2018). Ikaros family zinc finger 1 regulates dendritic cell development and function in humans. Nat. Commun. *9*, 1239.

Dickinson, R.E., Milne, P., Jardine, L., Zandi, S., Swierczek, S.I., McGovern, N., Cookson, S., Ferozepurwalla, Z., Langridge, A., Pagan, S., et al. (2014). The evolution of cellular deficiency in GATA2 mutation. Blood *123*, 863–874.

Dobin, A., and Gingeras, T.R. (2015). Mapping RNA-seq reads with STAR. Curr. Protoc. Bioinformatics *51*, 11.14.1–11.14.19.

Durinck, S., Moreau, Y., Kasprzyk, A., Davis, S., De Moor, B., Brazma, A., and Huber, W. (2005). BioMart and Bioconductor: a powerful link between biological databases and microarray data analysis. Bioinformatics *21*, 3439–3440.

Giladi, A., Paul, F., Herzog, Y., Lubling, Y., Weiner, A., Yofe, I., Jaitin, D., Cabezas-Wallscheid, N., Dress, R., Ginhoux, F., et al. (2018). Single-cell characterization of haematopoietic progenitors and their trajectories in homeostasis and perturbed haematopoiesis. Nat. Cell Biol. *20*, 836–846.

Grajales-Reyes, G.E., Iwata, A., Albring, J., Wu, X., Tussiwand, R., Kc, W., Kretzer, N.M., Briseño, C.G., Durai, V., Bagadia, P., et al. (2015). Batf3 maintains autoactivation of Irf8 for commitment of a CD8α(+) conventional DC clonogenic progenitor. Nat. Immunol. *16*, 708–717.

Granot, T., Senda, T., Carpenter, D.J., Matsuoka, N., Weiner, J., Gordon, C.L., Miron, M., Kumar, B.V., Griesemer, A., Ho, S.-H., et al. (2017). Dendritic cells display subset and tissue-specific maturation dynamics over human life. Immunity *46*, 504–515.

Guilliams, M., Dutertre, C.A., Scott, C.L., McGovern, N., Sichien, D., Chakarov, S., Van Gassen, S., Chen, J., Poidinger, M., De Prijck, S., et al. (2016). Unsupervised high-dimensional analysis aligns dendritic cells across tissues and species. Immunity *45*, 669–684.

Haghverdi, L., Buettner, F., and Theis, F.J. (2015). Diffusion maps for high-dimensional single-cell analysis of differentiation data. Bioinformatics *31*, 2989–2998.

Hambleton, S., Salem, S., Bustamante, J., Bigley, V., Boisson-Dupuis, S., Azevedo, J., Fortin, A., Haniffa, M., Ceron-Gutierrez, L., Bacon, C.M., et al. (2011). IRF8 mutations and human dendritic-cell immunodeficiency. N. Engl. J. Med. *365*, 127–138.

Helft, J., Anjos-Afonso, F., van der Veen, A.G., Chakravarty, P., Bonnet, D., and Reis e Sousa, C. (2017). Dendritic cell lineage potential in human early hematopoietic progenitors. Cell Rep. *20*, 529–537.

Kirkling, M.E., Cytlak, U., Lau, C.M., Lewis, K.L., Resteu, A., Khodadadi-Jamayran, A., Siebel, C.W., Salmon, H., Merad, M., Tsirigos, A., et al. (2018). Notch signaling facilitates in vitro generation of cross-presenting classical dendritic cells. Cell Rep. *23*, 3658–3672.e6.

Kiselev, V.Y., Kirschner, K., Schaub, M.T., Andrews, T., Yiu, A., Chandra, T., Natarajan, K.N., Reik, W., Barahona, M., Green, A.R., and Hemberg, M. (2017). SC3: consensus clustering of single-cell RNA-seq data. Nat. Methods *14*, 483–486.

Kong, X.F., Martinez-Barricarte, R., Kennedy, J., Mele, F., Lazarov, T., Deenick, E.K., Ma, C.S., Breton, G., Lucero, K.B., Langlais, D., et al. (2018). Disruption of an antimycobacterial circuit between dendritic and helper T cells in human SPPL2a deficiency. Nat. Immunol. *19*, 973–985.

Korenfeld, D., Gorvel, L., Munk, A., Man, J., Schaffer, A., Tung, T., Mann, C., and Klechevsky, E. (2017). A type of human skin dendritic cell marked by CD5 is associated with the development of inflammatory skin disease. JCI Insight *2*, 2.

Kurotaki, D., Yamamoto, M., Nishiyama, A., Uno, K., Ban, T., Ichino, M., Sasaki, H., Matsunaga, S., Yoshinari, M., Ryo, A., et al. (2014). IRF8 inhibits C/EBPα activity to restrain mononuclear phagocyte progenitors from differentiating into neutrophils. Nat. Commun. *5*, 4978.

Laurenti, E., and Göttgens, B. (2018). From haematopoietic stem cells to complex differentiation landscapes. Nature *553*, 418–426.

Lee, J., Breton, G., Oliveira, T.Y., Zhou, Y.J., Aljoufi, A., Puhr, S., Cameron, M.J., Sékaly, R.P., Nussenzweig, M.C., and Liu, K. (2015). Restricted dendritic cell and monocyte progenitors in human cord blood and bone marrow. J. Exp. Med. *212*, 385–399.

Lee, J., Zhou, Y.J., Ma, W., Zhang, W., Aljoufi, A., Luh, T., Lucero, K., Liang, D., Thomsen, M., Bhagat, G., et al. (2017). Lineage specification of human dendritic cells is marked by IRF8 expression in hematopoietic stem cells and multipotent progenitors. Nat. Immunol. *18*, 877–888.

Li, H., Handsaker, B., Wysoker, A., Fennell, T., Ruan, J., Homer, N., Marth, G., Abecasis, G., and Durbin, R.; 1000 Genome Project Data Processing Subgroup (2009). The sequence alignment/map format and SAMtools. Bioinformatics *25*, 2078–2079.

Macosko, E.Z., Basu, A., Satija, R., Nemesh, J., Shekhar, K., Goldman, M., Tirosh, I., Bialas, A.R., Kamitaki, N., Martersteck, E.M., et al. (2015). Highly parallel genome-wide expression profiling of individual cells using nanoliter droplets. Cell *161*, 1202–1214.

Matsui, T., Connolly, J.E., Michnevitz, M., Chaussabel, D., Yu, C.I., Glaser, C., Tindle, S., Pypaert, M., Freitas, H., Piqueras, B., et al. (2009). CD2 distinguishes two subsets of human plasmacytoid dendritic cells with distinct phenotype and functions. J. Immunol. *182*, 6815–6823.

McCarthy, D.J., Campbell, K.R., Lun, A.T., and Wills, Q.F. (2017). Scater: pre-processing, quality control, normalization and visualization of single-cell RNA-seq data in R. Bioinformatics *33*, 1179–1186.

Murphy, T.L., Grajales-Reyes, G.E., Wu, X., Tussiwand, R., Briseño, C.G., Iwata, A., Kretzer, N.M., Durai, V., and Murphy, K.M. (2016). Transcriptional control of dendritic cell development. Annu. Rev. Immunol. *34*, 93–119.

Naik, S.H., Perié, L., Swart, E., Gerlach, C., van Rooij, N., de Boer, R.J., and Schumacher, T.N. (2013). Diverse and heritable lineage imprinting of early haematopoietic progenitors. Nature *496*, 229–232.

Nakano, T., Kodama, H., and Honjo, T. (1994). Generation of lymphohematopoietic cells from embryonic stem cells in culture. Science *265*, 1098–1101.

Notta, F., Zandi, S., Takayama, N., Dobson, S., Gan, O.I., Wilson, G., Kaufmann, K.B., McLeod, J., Laurenti, E., Dunant, C.F., et al. (2016). Distinct routes of lineage development reshape the human blood hierarchy across ontogeny. Science *351*, aab2116.

Ouwehand, W.H. (2019). Whole-genome sequencing of rare disease patients in a national healthcare system. bioRxiv. https://doi.org/10.1101/507244.

Paul, F., Arkin, Y., Giladi, A., Jaitin, D.A., Kenigsberg, E., Keren-Shaul, H., Winter, D., Lara-Astiaso, D., Gury, M., Weiner, A., et al. (2015). Transcriptional heterogeneity and lineage commitment in myeloid progenitors. Cell *163*, 1663–1677.

Pellin, D., Loperfido, M., Baricordi, C., Wolock, S.L., Montepeloso, A., Weinberg, O.K., Biffi, A., Klein, A.M., and Biasco, L. (2019). A comprehensive single cell transcriptional landscape of human hematopoietic progenitors. Nat. Commun. *10*, 2395.

Picelli, S., Faridani, O.R., Björklund, A.K., Winberg, G., Sagasser, S., and Sandberg, R. (2014). Full-length RNA-seq from single cells using Smart-seq2. Nat. Protoc. *9*, 171–181.

Risso, D., Ngai, J., Speed, T.P., and Dudoit, S. (2014). Normalization of RNA-seq data using factor analysis of control genes or samples. Nat. Biotechnol. *32*, 896–902.

Salem, S., Langlais, D., Lefebvre, F., Bourque, G., Bigley, V., Haniffa, M., Casanova, J.L., Burk, D., Berghuis, A., Butler, K.M., et al. (2014). Functional characterization of the human dendritic cell immunodeficiency associated with the IRF8(K108E) mutation. Blood *124*, 1894–1904.

Satpathy, A.T., Briseño, C.G., Cai, X., Michael, D.G., Chou, C., Hsiung, S., Bhattacharya, D., Speck, N.A., and Egawa, T. (2014). Runx1 and Cbfβ regulate the development of Flt3+ dendritic cell progenitors and restrict myeloproliferative disorder. Blood *123*, 2968–2977.

Schlitzer, A., Sivakamasundari, V., Chen, J., Sumatoh, H.R., Schreuder, J., Lum, J., Malleret, B., Zhang, S., Larbi, A., Zolezzi, F., et al. (2015). Identification of cDC1- and cDC2-committed DC progenitors reveals early lineage priming at the common DC progenitor stage in the bone marrow. Nat. Immunol. *16*, 718–728.

Schrøder, M., Melum, G.R., Landsverk, O.J., Bujko, A., Yaqub, S., Gran, E., Aamodt, H., Bækkevold, E.S., Jahnsen, F.L., and Richter, L. (2016). CD1c-expression by monocytes: implications for the use of commercial CD1c+ dendritic cell isolation kits. PLoS ONE *11*, e0157387.

See, P., Dutertre, C.A., Chen, J., Günther, P., McGovern, N., Irac, S.E., Gunawan, M., Beyer, M., Händler, K., Duan, K., et al. (2017). Mapping the human DC lineage through the integration of high-dimensional techniques. Science *356*, eaag3009.

Sichien, D., Scott, C.L., Martens, L., Vanderkerken, M., Van Gassen, S., Plantinga, M., Joeris, T., De Prijck, S., Vanhoutte, L., Vanheerswynghels, M., et al. (2016). IRF8 transcription factor controls survival and function of terminally differentiated conventional and plasmacytoid dendritic cells, respectively. Immunity *45*, 626–640.

Spinelli, L., Carpentier, S., Montañana Sanchis, F., Dalod, M., and Vu Manh, T.P. (2015). BubbleGUM: automatic extraction of phenotype molecular signatures and comprehensive visualization of multiple gene set enrichment analyses. BMC Genomics *16*, 814.

Street, K., Risso, D., Fletcher, R.B., Das, D., Ngai, J., Yosef, N., Purdom, E., and Dudoit, S. (2018). Slingshot: cell lineage and pseudotime inference for single-cell transcriptomics. BMC Genomics *19*, 477.

Tailor, P., Tamura, T., Morse, H.C., 3rd, and Ozato, K. (2008). The BXH2 mutation in IRF8 differentially impairs dendritic cell subset development in the mouse. Blood *111*, 1942–1945.

Thaventhiran, J.E.D., Lango Allen, H., Burren, O.S., Farmery, J.H.R., Staples, E., Zhang, Z., Rae, W., Greene, D., Simeoni, I., Maimaris, J., et al. (2018). Whole genome sequencing of primary immunodeficiency reveals a role for common and rare variants in coding and non-coding sequences. bioRxiv. https://doi.org/10.1101/499988.

Velten, L., Haas, S.F., Raffel, S., Blaszkiewicz, S., Islam, S., Hennig, B.P., Hirche, C., Lutz, C., Buss, E.C., Nowak, D., et al. (2017). Human haematopoietic stem cell lineage commitment is a continuous process. Nat. Cell Biol. *19*, 271–281.

Villani, A.C., Satija, R., Reynolds, G., Sarkizova, S., Shekhar, K., Fletcher, J., Griesbeck, M., Butler, A., Zheng, S., Lazo, S., et al. (2017). Single-cell RNA-seq reveals new types of human blood dendritic cells, monocytes, and progenitors. Science *356*, eaah4573.

Wilson, A., Murphy, M.J., Oskarsson, T., Kaloulis, K., Bettess, M.D., Oser, G.M., Pasche, A.C., Knabenhans, C., Macdonald, H.R., and Trumpp, A. (2004). c-Myc controls the balance between hematopoietic stem cell self-renewal and differentiation. Genes Dev. *18*, 2747–2763.

Yin, X., Yu, H., Jin, X., Li, J., Guo, H., Shi, Q., Yin, Z., Xu, Y., Wang, X., Liu, R., et al. (2017). Human blood CD1c+ dendritic cells encompass CD5high and CD5low subsets that differ significantly in phenotype, gene expression, and functions. J. Immunol. *198*, 1553–1564.

CellPress
OPEN ACCESS

STAR★METHODS

KEY RESOURCES TABLE

REAGENT or RESOURCE	SOURCE	IDENTIFIER
Antibodies		
Mouse anti-APC 176Yb, clone APC003	Fluidigm	Cat# 3176007B
Mouse anti-human AXL APC, clone 108724	R&D Systems	Cat# FAB154A
Mouse anti-human AXL purified, clone 108724	R&D Systems	Cat# MAB154; RRID:AB_2062558
Mouse anti-human BTLA 163Dy, clone MIH26	Fluidigm	Cat# 3163009B
Mouse anti-human BTLA BV650, clone J168-540	BD Biosciences	Cat# 564803; RRID:AB_2738962
Mouse anti-human CD1c APC-Cy7, clone L161	BioLegend	Cat# 331520; RRID:AB_10644008
Mouse anti-human CD1c PE-Cy7, clone L161	BioLegend	Cat# 331516; RRID:AB_2275574
Mouse anti-human CD1c PerCP-Cy5.5, clone L161	BioLegend	Cat# 331513; RRID:AB_1227536
Mouse anti-human CD1c purified, clone L161	BioLegend	Cat# 331502; RRID:AB_1088995
Mouse anti-human CD2 151Eu, clone TS1/8	Fluidigm	Cat# 3151003B
Mouse anti-human CD2 BV421, clone TS1/8	BioLegend	Cat# 309217; RRID:AB_10915139
Mouse anti-human CD2 PE-CF594, clone RPA-2.10	BD Biosciences	Cat# 562300; RRID:AB_11153492
Mouse anti-human CD3 AF700, clone SK7 (Leu-4)	BioLegend	Cat# 344822; RRID:AB_2563420
Mouse anti-human CD3 FITC, clone SK7(Leu-4)	BD Biosciences	Cat# 345763
Mouse anti-human CD3 PE, clone SK7(Leu9)	BD Biosciences	Cat# 345765
Mouse anti-human CD5 BUV737, clone UCHT2	BD Biosciences	Cat# 564451; RRID:AB_2714177
Mouse anti-human CD5 purified, clone L17F12	BioLegend	Cat# 364002; RRID:AB_2564477
Mouse anti-human CD7 FITC, clone Leu-9	BD Biosciences	Cat# 347483; RRID:AB_400309
Mouse anti-human CD7 PE, clone M-T701	BD Biosciences	Cat# 332774
Mouse anti-human CD10 156Gd, clone HI10a	Fluidigm	Cat# 3156001B
Mouse anti-human CD10 BV650, clone HI10a	BD Biosciences	Cat# 563734; RRID:AB_2738393
Mouse anti-human CD11b 144Nd, clone ICRF44	Fluidigm	Cat# 3144001B
Mouse anti-human CD11c 159Tb, clone Bu15	Fluidigm	Cat# 3159001B
Mouse anti-human CD11c AF700, clone B-ly6	BD Biosciences	Cat# 561352; RRID:AB_10612006
Mouse anti-human CD11c APC-Cy7, clone Bu15	BioLegend	Cat# 337218; RRID:AB_10662746
Mouse anti-human CD11c BV711, clone B-ly6	BioLegend	Cat# 301630; RRID:AB_2562192
Mouse anti-human CD14 BV650, clone M5E2	BioLegend	Cat# 301835; RRID:AB_11204241
Mouse anti-human CD14 FITC, clone M5E2	BD Biosciences	Cat# 555397; RRID:AB_395798
Mouse anti-human CD14 PE, clone M5E2	BD Biosciences	Cat# 555398; RRID:AB_395799
Mouse anti-human CD14 PE-Cy7, clone HCD14	BioLegend	Cat# 325618; RRID:AB_830691
Mouse anti-human CD14 purified, clone M5E2	BioLegend	Cat# 301802; RRID:AB_314184
Mouse anti-human CD15 164Dy, clone W6D3	Fluidigm	Cat# 3164001B
Mouse anti-human CD15 BUV395, clone HI98	BD Biosciences	Cat# 563872; RRID:AB_2738461
Mouse anti-human CD15 BV605, clone W6D3	BD Biosciences	Cat# 562979; RRID:AB_2744292
Mouse anti-human CD16 209Bi, clone 3G8	Fluidigm	Cat# 3209002B
Mouse anti-human CD16 AF700, clone 3G8	BioLegend	Cat# 302026; RRID:AB_2278418
Mouse anti-human CD16 FITC, clone 3G8	BD Biosciences	Cat# 335035
Mouse anti-human CD16 PE, clone 3G8	BD Biosciences	Cat# 555407; RRID:AB_395807
Mouse anti-human CD16 PE-Dazzle594, clone 3G8	BioLegend	Cat# 302054; RRID:AB_2563639
Mouse anti-human CD19 AF700, clone 4G7/HIB19	BioLegend	Cat# 302226; RRID:AB_493751
Mouse anti-human CD19 FITC, clone 4G7	BD Biosciences	Cat# 345776
Mouse anti-human CD19 PE, clone HIB19	BD Biosciences	Cat# 555413; RRID:AB_395813
Mouse anti-human CD20 AF700, clone L27/2H7	BioLegend	Cat# 302322; RRID:AB_493753
Mouse anti-human CD20 FITC, clone L27	BD Biosciences	Cat# 345792

(*Continued on next page*)

Continued

REAGENT or RESOURCE	SOURCE	IDENTIFIER
Mouse anti-human CD20 PE, clone L27	BD Biosciences	Cat# 345793
Mouse anti-human CD33 158Gd, clone WM53	Fluidigm	Cat# 3158001B
Mouse anti-human CD33 APC, clone P67.6	BD Biosciences	Cat# 345800
Mouse anti-human CD33 BV711, clone WM53	BD Biosciences	Cat# 563171; RRID:AB_2738045
Mouse anti-human CD34 166Er, clone 581	Fluidigm	Cat# 3166012B
Mouse anti-human CD34 APC-Cy7, clone 581	BioLegend	Cat# 343514; RRID:AB_1877168
Mouse anti-human CD34 BV605, clone 581	BioLegend	Cat# 343529; RRID:AB_2562193
Mouse anti-human CD34 FITC, clone 8G12	BD Biosciences	Cat# 345801
Mouse anti-human CD34 PE-CF594, clone 581	BD Biosciences	Cat# 562383; RRID:AB_11154586
Mouse anti-human CD36 155Gd, clone 5-271	Fluidigm	Cat# 3155012B
Mouse anti-human CD38 PE-Cy7, clone HB7	BD Biosciences	Cat# 335825
Mouse anti-human CD38 purified, clone HB-7	BioLegend	Cat# 356602; RRID:AB_2561794
Mouse anti-human CD45 89Y, clone HI30	Fluidigm	Cat# 3089003B
Mouse anti-human CD45 APC-Cy7, clone 2D1	BD Biosciences	Cat# 557833; RRID:AB_396891
Mouse anti-human CD45 V450, clone 2D1	BD Biosciences	Cat# 642275; RRID:AB_1645755
Mouse anti-human CD45RA 153Eu, clone HI100	Fluidigm	Cat# 3153001B
Mouse anti-human CD45RA BV510, clone HI100	BioLegend	Cat# 304142; RRID:AB_2561947
Rat anti-human CD52 PE, clone YTH34.5	Bio-Rad	Cat# SFL1642PE; RRID:AB_324131
Mouse anti-human CD56 FITC, clone NCAM16.2	BD Biosciences	Cat# 345811
Mouse anti-human CD88 PE, clone S5/1	BioLegend	Cat# 344304; RRID:AB_2067175
Mouse anti-human CD88 purified, clone C5AR	BioLegend	Cat# 344302; RRID:AB_2259318
Mouse anti-human CD90 161Dy, clone 5E10	Fluidigm	Cat# 3161009
Mouse anti-human CD90 AF700, clone 5E10	BioLegend	Cat# 328120; RRID:AB_2203302
Mouse anti-human CD90 PerCP-Cy5.5, clone 5E10	BioLegend	Cat# 328118; RRID:AB_2303335
Human anti-human CD100 APC-Vio770, clone REA316	Miltenyi Biotec	Cat# 130-104-604; RRID:AB_2654328
Mouse anti-human CD100 purified, clone A8	BioLegend	Cat# 328401; RRID:AB_1236386
Mouse anti-human CD115 purified, clone 9-4D2-1E4	BioLegend	Cat# 347302; RRID:AB_2085375
Mouse anti-human CD116 BV421, clone hGMCSFR-M1	BD Biosciences	Cat# 564045; RRID:AB_2738561
Mouse anti-human CD116 BV650, clone hGMCSFR-M1	BD Biosciences	Cat# 564044; RRID:AB_2738560
Mouse anti-human CD116 purified, clone 4H1	BioLegend	Cat# 305902; RRID:AB_314568
Mouse anti-human CD117 BV605, clone 104D2	BD Biosciences	Cat# 562687; RRID:AB_2737721
Mouse anti-human CD117 PE, clone 104D2	BD Biosciences	Cat# 332785
Mouse anti-human CD117 purified, clone 104D2	BioLegend	Cat# 313201; RRID:AB_314980
Mouse anti-human CD123 143Nd, clone 6H6	Fluidigm	Cat# 3143014B
Mouse anti-human CD123 BUV395, clone 7G3	BD Biosciences	Cat# 564195; RRID:AB_2714171
Mouse anti-human CD123 BV421, clone 6H6	BioLegend	Cat# 306018; RRID:AB_10962571
Mouse anti-human CD123 PerCP-Cy5.5, clone 7G3	BD Biosciences	Cat# 558714; RRID:AB_1645547
Mouse anti-human CD135 BV711, clone 4G8	BD Biosciences	Cat# 563908; RRID:AB_2738479
Mouse anti-human CD135 purified, clone BV10A4H2	BioLegend	Cat# 313302; RRID:AB_314987
Mouse anti-human CD141 BV510, clone 1A4	BD Biosciences	Cat# 563298; RRID:AB_2728103
Mouse anti-human CD141 purified, clone M80	BioLegend	Cat# 344102; RRID:AB_2201808
Mouse anti-human CD161 PE-Cy7, clone HP-3G10	Thermo Fisher Scientific	Cat# 25-1619-42; RRID:AB_10807086
Mouse anti-human CD303 147Sm, clone 201A	Fluidigm	Cat# 3147009B
Mouse anti-human CD303 APC, clone 201A	BioLegend	Cat# 354206; RRID:AB_11150412
Mouse anti-human CD303 BV605, clone 201A	BioLegend	Cat# 354224; RRID:AB_2572149
Mouse anti-human CD304 169Tm, clone 12C2	Fluidigm	Cat# 3169018B
Mouse anti-human CD304 APC, clone 12C2	BioLegend	Cat# 354506; RRID:AB_11219600
Mouse anti-human CD304 BV605, clone U21-1283	BD Biosciences	Cat# 743130; RRID:AB_2741297
Mouse anti-human CLEC9A PE, clone 8F9	BioLegend	Cat# 353804; RRID:AB_10965546

(*Continued on next page*)

Continued

REAGENT or RESOURCE	SOURCE	IDENTIFIER
Mouse anti-human CLEC9A purified, clone 8F9	BioLegend	Cat# 353802; RRID:AB_10983070
Rat anti-human CX3CR1 APC, clone 2A9-1	BioLegend	Cat# 341610; RRID:AB_2087424
Mouse anti-human FceRI 150Nd, clone AER-37 (CRA-1)	Fluidigm	Cat# 3150027B
Mouse anti-FITC purified, clone FIT-22	BioLegend	Cat# 408305; RRID:AB_2563769
Mouse anti-human HLA-DR 173Yb, clone L243	Fluidigm	Cat# 3173005B
Mouse anti-human HLA-DR AF700, clone G46-6	BD Biosciences	Cat# 560743; RRID:AB_1727526
Mouse anti-human HLA-DR BV785, clone L243	BioLegend	Cat# 307642; RRID:AB_2563461
Mouse anti-human HLA-DR PerCP-Cy5.5, clone L243	BioLegend	Cat# 307629; RRID:AB_893575
Mouse anti-human ID2 purified, clone 4E12G5	Thermo Fisher Scientific	Cat# MA5-17095; RRID:AB_2538566
Mouse anti-human IFN-a PE, clone LT27:295	Miltenyi Biotec	Cat# 130-092-601; RRID:AB_871560
Rat anti-human IL-10 APC, clone JES3-9D7	BioLegend	Cat# 501410; RRID:AB_315176
Mouse anti-human IL-12p40/p70 BV421, clone C8.6	BD Biosciences	Cat# 565023; RRID:AB_2739045
Mouse anti-human IL-1b FITC, clone JK1B-1	BioLegend	Cat# 508206; RRID:AB_345362
Mouse anti-human IL-8 PE-Cy7, clone E8N1	BioLegend	Cat# 511416; RRID:AB_2565291
Rat anti-human IRF4 PE, clone 3E4	Thermo Fisher Scientific	Cat# 12-9858-80; RRID:AB_10853179
Mouse anti-human IRF4 purified, clone IRF4.3E4	BioLegend	Cat# 646402; RRID:AB_2280462
Mouse anti-human IRF8 efluor710, clone 3GYWCH	Thermo Fisher Scientific	Cat# 46-9852-80; RRID:AB_2573903
Mouse anti-human IRF8 purified, clone GW4CML3	Thermo Fisher Scientific	Cat# 14-7888-82; RRID:AB_2572907
Goat anti-human KLF4 APC, clone POLY	R&D Systems	Cat# IC3640A; RRID:AB_2044690
Mouse anti-PE purified, clone PE001	BioLegend	Cat# 408105; RRID:AB_2563787
Mouse anti-human SIGLEC-6 purified, clone 767329	R&D Systems	Cat# MAB2859
Mouse anti-human SIRPA purified, clone 15-414	BioLegend	Cat# 372102; RRID:AB_2629807
Mouse anti-human SIRPA/B AF700, clone SE5A5	BioLegend	Cat# 323816; RRID:AB_2687275
Mouse anti-human SIRPA/B APC, clone SE5A5	BioLegend	Cat# 323809; RRID:AB_11219399
Mouse anti-human SIRPA/B PE, clone SE5A5	BioLegend	Cat# 323805; RRID:AB_830704
Mouse anti-human SLAN PE, clone DD1	Miltenyi Biotec	Cat# 130-093-029; RRID:AB_871582
Biological Samples		
Healthy human peripheral blood mononuclear cells (PBMC)	Newcastle Biobank	REC 12/NE/0395
Healthy human bone marrow mononuclear cells (BMMC)	Newcastle Bone and Joint Biobank and Project ethics	REC 14/NE/1212<br>REC 13/NE/1136
IRF8 patient tissues	As previously described in Hambleton et al. (2011) and Bigley et al. (2018). Adult material project specific. Paediatric material through Newcastle Biobank	REC 08/H0906/72<br>REC 16/NE/0002
Chemicals, Peptides, and Recombinant Proteins		
Carboxyfluorescein succinimidyl ester (CFSE, final concentration: 0.5 μM)	Invitrogen	Cat# C34554
Lymphoprep density gradient solution	Stem Cell Technologies	Cat# 07851
Dulbecco's phosphate-buffered saline (PBS)	Sigma	Cat# D8537-500ml
Fetal bovine serum, South American origin, batch 50115	Labtech	Cat# FCS-SA/500
Ethylenediaminetetraacetic acid (EDTA)	Sigma	Cat# E7889
DAPI	Partec	Cat# D8417
Zombie UV Fixable Viability Kit	Biolegend	Cat# 423108
MEM Alpha Medium w/o Nucleosides (αMEM)	Life Technologies	Cat# 22561-021
RPMI-1640	Sigma	Cat# R0883-500ml
L-Glutamine	Sigma	Cat#G7513-100ml
Penicillin-Streptomycin	Sigma	Cat# P0781

(Continued on next page)

Continued

REAGENT or RESOURCE	SOURCE	IDENTIFIER
Recombinant Human Stem Cell Factor (SCF)	Immunotools	Cat# 11343325
Recombinant human Granulocyte-Macrophage-Colony Stimulating Factor (GM-CSF)	R&D systems	Cat# CAA26822
Recombinant Human Flt-3 ligand	Immunotools	Cat# 11343305
IgG from mouse serum	Sigma	Cat# I5381
Triton X-100	Sigma	Cat# 9002-93-1
Recombinant RNase inhibitor (2U/μl)	Takara Clontech	Cat# 2313B
polyinosinic:polycytidylic acid (poly(I:C), final concentration: 10 μg/ml)	Invivogen	Cat #tlrl-pic
Lipopolysaccharide (LPS, final concentration: 5ng/ml)	Sigma	Cat# L2654
CL075 (final concentration: 1 μg/ml)	Invivogen	Cat# tlrl-c75
CpG oligonucleotide (ODN 2216) (final concentration: 7.5μM)	Invivogen	Cat# tlrl-2216
Brefeldin A (final concentration: 10 μg/ml)	Sigma	Cat# B7651-5MG
Formaldehyde	TAAB Laboratories	Cat# F017/3
Cisplatin	Fluidigm	Cat# 201064
Irridium	Fluidigm	Cat# 201192A
EQ Four Element Calibration Beads	Fluidigm	Cat# 201078
Critical Commercial Assays		
Foxp3 Transcription Factor Staining Buffer Set	Thermo Fisher Scientific	Cat# 00-5523
Maxpar antibody labeling kit	Fluidigm	N/A
Deposited Data		
Single cell RNA sequencing data	Human BM progenitors	GSE142999 https://www.ncbi.nlm.nih.gov/geo/query/acc.cgi?acc=GSE142999
Single cell RNA sequencing data	Human BM dendritic cells and precursors	GSE143002 https://www.ncbi.nlm.nih.gov/geo/query/acc.cgi?acc=GSE143002
Single cell RNA sequencing data	Human peripheral blood CD123+ dendritic cell precursors	GSE143158 https://www.ncbi.nlm.nih.gov/geo/query/acc.cgi?acc=GSE143158
Experimental Models: Cell Lines		
Mouse OP9 cell line	ATCC	CRL-2749
Software and Algorithms		
FACSDIVA 8.0.1 or 8.0 software	BD Biosciences	N/A
FlowJo 10.5.3	Treestar, Inc	N/A
CyTOF software v 6.7.1014	Fluidigm	https://www.fluidigm.com/software
GraphPad Prism v5.0a	GraphPad Software, Inc.	N/A
Nanostring nSolver	NanoString	https://www.nanostring.com/products/analysis-software/nsolver
Other		
LSRFortessa X20	BD Biosciences	H656385K01
FACS Aria Fusion Sorter	BD Biosciences	P656700000018
Helios CyTOF	Fluidigm	N/A
Greiner CELLSTAR® 96 well plates	Greiner	M9436
Corning® 96 Well TC-Treated Microplates size 96 wells, clear, polystyrene, round bottom, case of 50 (individually wrapped), sterile, lid	Corning	CLS3799
50 μm sterile filter	Sysmex Partec	04-004-2327

RESOURCE AVAILABILITY

Lead Contact

Further information and requests for resources and reagents should be directed to and will be fulfilled by the Lead Contact, Venetia Bigley (venetia.bigley@ncl.ac.uk)

Materials Availability

This study did not generate new unique reagents

Data and Code Availability

Single cell RNA-Seq datasets generated in this study are deposited in the Genome Expression Omnibus under the following accession numbers:

Human BM progenitors GSE142999
(https://www.ncbi.nlm.nih.gov/geo/query/acc.cgi?acc=GSE142999)
Human BM dendritic cells and precursors GSE143002
(https://www.ncbi.nlm.nih.gov/geo/query/acc.cgi?acc=GSE143002)
Human PB dendritic cell precursors GSE143158
(https://www.ncbi.nlm.nih.gov/geo/query/acc.cgi?acc=GSE143158)

Subject Details

The study was performed in accordance with the Declaration of Helsinki. Written informed consent was obtained from participants or their parents. The study was approved by local review board NRES Committee North East-Newcastle and North Tyneside: 08/H0906/72 and REC 14/NE/1136; REC 14/NE/1212, 17/NE/0361.

Patients and healthy donors

Details of individuals carrying *IRF8*$^{K108E/K108E}$ and *IRF8*$^{A83C/R291Q}$ are previously described (Hambleton et al., 2011; Bigley et al., 2018). The kindred carrying *IRF8*^{V426fs} was identified through BRIDGE whole genome sequencing initiative (Ouwehand, 2019; Thaventhiran et al., 2018). Details and molecular characterization of the mutation will be published independently. Healthy bone marrow was obtained from hematopoietic stem cell transplant donors (pediatric or adult) or from hip arthroplasty (adult).

METHOD DETAILS

Flow cytometry and cell sorting

Healthy control mononuclear cells from peripheral blood (PBMC) or bone marrow (BMMC), isolated by density centrifugation, were stained in aliquots of 3 x10^6 cells/50 µL of Dulbecco's phosphate-buffered saline (PBS) with 0.1%–2% fetal calf serum (FCS, GIBCO) and 0.4% EDTA for 30min at room temperature (RT). Non-specific staining was blocked with 3 µL mouse IgG prior to staining. Dead cells, usually < 5%, were excluded by DAPI (Partec) or Zombie (Biolegend) staining. Analysis was performed with a BD LSRFortessa X20 and sorting with a FACS Aria Fusion Sorter (BD Biosciences) running BD FACSDIVA 8.0.1 or 8.0 software, respectively. Purity of > 98% was achieved in sorted populations. Data were processed with FlowJo 10.5.3 (Tree Star, Inc). Intracellular staining was performed after surface staining, lysis and fixation (eBioscience) according to manufacturer's instructions. Absolute cell counts were obtained using TruCount tubes (BD Biosciences) with 200 µL whole blood and 900 µL of red cell lysis buffer added after staining. For proliferation studies, BMMC were stained with carboxyfluorescein succinimidyl ester (CFSE, 0.5 µM, Invitrogen) prior to FACS purification according to gating strategy in Figure 4A. and cultured in standard DC differentiation conditions. CFSE dilution was assessed by flow cytometry on day 3. CD34$^+$progenitors and CD14$^+$monocytes were included as positive and negative controls, respectively. A full list of antibodies is provided in the Key Resources Table.

In vitro generation of dendritic cells

FACS-purified human PB or BM CD34$^+$ progenitors, progenitor subsets or pre-DC were cultured in 96 well U-bottomed plates (Corning) with pre-seeded OP9 stromal cells (5000vwell) in 200 µL alpha-MEM (αMEM, GIBCO) supplemented with 1% penicillin/streptomycin (Sigma), 10% FCS, 20ng/ml granulocyte-macrophage colony-stimulating factor (GM-CSF, R&D systems), 100ng/ml Flt3-ligand (FLT3, Immunotools), 20ng/ml stem cell factor (SCF, Immunotools). CD34$^+$ cells were seeded at 3000/well or 500/well for serial time points. Pre-DC were seeded at 500-3000 cells/well, determined by the number of cells available after FACS-purification. Half the volume of media, with cytokines, was replaced weekly. Cells were harvested on ice at day 14 or 21; or at days 3, 5, 7, 9, 11, 14 and 21 for serial time points, passed through a 50 µm filter (Sysmex Partec), washed in PBS, and stained for flow cytometric analysis or FACS-purification.

Dendritic cell functional analysis

TLR stimulation: PBMC or *in vitro* generated cells were incubated in RPMI plus 10% FCS in the presence of polyinosinic:polycytidylic acid (poly(I:C) (10 µg/ml, Invivogen), lipopolysaccharide (LPS) (5ng/ml, Sigma), CL075 (1 µg/ml, Invivogen) and CpG (7.5µM,

Invivogen) for 14h at 37°C, 5% CO_2 with addition of Brefaldin A (10 μg/ml, eBioscience) after 3h. Dead cells (usually < 30%) were excluded with Zombie amine dye (Biolegend). Intracellular cytokine staining was performed after surface staining, fixation and permeabilization (eBioscience) according to manufacturer's instructions.

NanoString nCounter analysis

Dendritic cell subsets and monocytes were FACS-purified (> 98% purity) from *ex vivo* PBMC or cells generated from BM CD34$^+$progenitors after 21 days in culture and lysed in RLT buffer containing 1% β-mercaptoethanol at a concentration of 2000 cells/μl. Samples were analyzed on the NanoString nCounter® platform using the Immunology V2 panel supplemented with 30 genes, as described in Kirkling et al. (2018).

Counts were normalized within the nSolver software (advanced analysis module version 1.1.4). The log2 transformed output data were analyzed using R v 3.3.3. For principal component analysis (PCA), genes with normalized expression values below 16 in more than half of the samples were removed (293/608 for *ex vivo* dataset and 288/608 for combined *ex vivo* and culture dataset). The remaining genes were used for the PC analyses.

A culture signature was derived by performing pairwise comparisons (two-tailed t test with Benjamini-Hochberg correction of p values) of all culture versus all *ex vivo* populations. 110 genes with adjusted p values < 0.05 (the 'culture signature') were excluded from further analysis. The remaining 210 genes were used to construct the combined *ex vivo* and culture-derived cell PCA plot.

Statistical computation of the signature genes for the blood CD1c$^+$DC subsets and monocytes was performed with Bubble GUM, a tool based on Gene Set Enrichment Analysis (GSEA) algorithm (Spinelli et al., 2015). Heatmaps were generated in R and display the scaled expression of the top signature genes across the 5 blood and 3 cultured subsets. 129 signature genes with significant FDR were identified for blood monocytes (top 32 based on fold change displayed on heatmap), 32 and 16 genes for CD14$^+$DC3 and CD5$^+$DC2, respectively. No signature genes were identified for the CD14$^-$BTLA$^-$DC3 or CD5$^-$BTLA$^+$DC2 fractions.

Single cell RNA sequencing

Single human PBMC or BMMC were index-sorted into 96-well round-bottom plates

containing 2 μL cold RNA lysis buffer (RNase-free water, 2U/μl RNase inhibitor and 0.2% Triton X-100, Sigma) (three BM progenitor plates) or SMARTer Dilution buffer (SMARTer Kit, Fluidigm) with the addition of 2U/μl RNase inhibitor (three BM precursor plates and one BM DC plate). Plates were immediately centrifugated at 500xg for 1 minute, frozen on dry ice then stored at −80oC. Each plate included 2 controls; one blank and one well containing purified mouse RNA. The reverse transcription (RT) was performed using an adapted Smart-Seq2 protocol (Picelli et al., 2014). Briefly, modifications included 21 PCR cycles and duplicate Ampure clean-up steps, following cDNA generation. The library prep was performed using the Nextera XT DNA Library Prep Kit. The Illumina HiSeq 4000 platform was employed to generate paired-end reads (75bp x 2).

Alignment of reads to the human reference genome

reads were trimmed based on quality with Trimmomatic v 0.36 (Bolger et al., 2014). Bases with quality scores below Q10 (inferred base cell accuracy below 90%) were trimmed and reads shorter than 60bp were dropped. The remaining reads were aligned in the STAR mapping algorithm v 2.4.0 (Dobin and Gingeras, 2015) to the human reference genome version GRCh38.p7 (GENCODE release 25) supplemented with External RNA Controls Consortium (ERCC) spike-in controls. The files were converted from SAM format to the more compressed BAM format with SAMtools v 1.3 (Li et al., 2009).The count tables were obtained using HTSEQ v 0.6.1 (Anders et al., 2015). ENSEMBL IDs were converted to HGNC gene names using biomaRt v 2.30.0 (Durinck et al., 2005).

Gene and cell filtering

further analysis of the data was undertaken in R and Rstudio v 1.0.143. Quantitative details are documented in Table S3. The Scater R package v 1.2.0 was used to perform cell and gene QC and filtering (McCarthy et al., 2017). To remove technical outlier genes with poor coverage, only genes expressed at > 2 counts in > 2 cells were retained (range across datasets 14,458-18,791 genes). Low quality cells were removed based on number of total features, total counts, percentage of counts derived from ERCC spike-ins and % of mitochondrial gene counts (> 20%) (Table S3). After filtering, the number of cells remaining out of the total FACS sorted for each dataset were: BM CD34$^+$ progenitors, 262/399; BM CD34^{int} pre- and mature DC, 244/260; PB pre-DC, 116/184. The normalization was performed with the RUVg method (Risso et al., 2014) combined with counts per million (CPM) adjustment for library size and log transformation [log2(CPM+1)] for all downstream analyses. Only the genes annotated as protein coding in the "gene_type" column of the GENCODE reference genome GTF file were retained. To minimize the effect of cell-division cycle on the clustering performed in future steps, genes associated with cell cycle activity were downloaded from Macosko et al. (2015) and removed from all our analyses. The number of protein-coding, non-cell cycle genes retained for each dataset were: BM CD34$^+$ progenitors, 12406; BM CD34^{int} pre- and mature DC, 12137; PB pre-DC, 10346 (Table S3).

Cell clustering

clustering was performed with all the protein-coding, non-cell cycle genes using the Single-Cell Consensus Clustering (SC3) R package v 1.3.18 (Kiselev et al., 2017). The SC3 tool requires the *k* number of number of clusters to be specified by the user. A range of clusters (2 to 15) was visualized and interrogated for each of the datasets. The output from the "sc3_estimate_k" function guided the minimum number of clusters to be considered for each of the datasets. Clustering solutions took into account cluster stability indices

('average silhouette width' > 0.45 and 'stability index' within SC3, detailed in Table S3), known cell phenotypes from index sorting parameters, cluster marker genes defined in previous literature and minimum number of expected populations within the dataset.

Heatmaps with marker genes were generated within SC3. The area under the receiver operating characteristic curve (AUROC) and p values assigned by a Wilcoxon signed rank test and corrected using the Holm method were used to define the marker genes (thresholds for statistics are stated in figure legends and Table S3). Clusters were annotated based on the top statistically significant marker genes from the SC3 output, and correlated with index-sorting phenotype and culture output.

tSNE analysis

the tSNE technique for dimensionality reduction was used to visualize the clusters. First, SC3 gene filter was applied to further remove genes with low expression, and those ubiquitously expressed. The remaining genes (quantified for each dataset in Table S3) were used for tSNE analysis with the Rtsne package v 0.13. An initial PCA step was introduced to reduce dimensionality and eliminate noise. Top principal components accounting for most variance (25%–35%) were retained for the tSNE algorithm. The number of PCs is stated in the relevant figure legends).

For the BM mature DC dataset, the DC2 and DC3 signatures were downloaded from Villani et al., 2017 (90 genes) (Villani et al., 2017). 71 of the genes were identified in our dataset and used for SC3 clustering and tSNE analysis (Table S2), as described above.

For the signature scores displayed on tSNE embeddings or as a boxplot, the normalized counts for all genes present both in our datasets and in the DC signatures identified in Villani et al., 2017 were rescaled from 0 to 1. The average scaled signature score was then displayed on tSNE plots produced as described above. Graphics were generated with the ggplot2 package v 3.0.0.

Diffusion maps and lineage tracing

diffusion maps were used to infer a pseudo-temporal ordering and reconstruct lineage branching (Haghverdi et al., 2015). All protein coding genes that were not known to play a role in cell cycle were used in the diffusion map calculation with the destiny tool v 2.14.0 (Angerer et al., 2016). An initial PCA step was employed to reduce noise, and PCs accounting for most variance (total of approximately 40% for both datasets) were retained for destiny. Diffusion components 1-3 were used for trajectory tracing with slingshot v 1.2.0 (Street et al., 2018) and visualized on 3D plots. Graphics were generated with the rgl package v 0.100.19.

Helios Mass Cytometer (CyTOF) analysis

Pre-conjugated antibodies (Fluidigm), purified antibodies conjugated to their respective lanthanide metals using the Maxpar antibody labeling kit (as per manufacturer's instructions; DVS Sciences) or fluorophore-conjugated primary with anti-fluorophore metal-conjugated secondary antibodies were used for surface or intracellular staining (Table S2; Key Resources Table).

Healthy control CD45^{+}lineage^{-} (CD3,19,20,56,161) PBMC (3x10^{6} cells) or BMMC (1.5x 10^{6}) were FACS-purified into 1ml CyTOF staining buffer (PBS plus 2% FCS). Cell staining was performed at room temperature in a final staining volume of 100ul. Centrifugation was performed at 500xg for 5 minutes unless otherwise stated. 'Barcoding' of PBMC and BMMC samples was achieved by staining with 0.5ug anti-CD45-Irr115 or anti-CD45-89Y, respectively, (30mins) in CyTOF staining buffer before washing twice in PBS. Barcoded PBMC and BMMC were combined before addition of 2.5 μM cisplatin for 5 minutes in PBS for live/dead cell discrimination, then washed promptly in CyTOF staining buffer. Successive primary and secondary surface staining was performed using approximately 0.5 μg of each antibody in CyTOF staining buffer (30mins) before washing twice with PBS. The cells were fixed in 500ml eBioscience fixation buffer (eBioscience FoxP3 fix perm kit) with the addition of 500 μL of 3.2% formaldehyde (final concentration 1.6%) and incubated for 30 minutes, before washing twice with eBioscience perm buffer. Cells were stained successively in perm buffer for 1hr each with intracellular primary and secondary antibodies then washed twice with PBS. Cells were resuspended in 500 μL 250nM Irridium in PBS (final concentration 125nM) and 500 μL 3.2% formaldehyde (final concentration 1.6%) and incubated for 1hr, before centrifugation and resuspension in 500 μL CyTOF wash buffer for overnight storage at 4°C. Prior to CyTOF acquisition, cells were washed twice in 200 μL MilliQ water (800xg for 8 minutes), counted, diluted to a maximum final concentration of 0.55x10^{6}/ml in MilliQ water and filtered through a 40 μm filter (BD). EQ beads were added (10% by volume) and 1.5x10^{6} cells were acquired on the Helios mass cytometer running CyTOF software v 6.7.1014.

tSNE analysis

within the CyTOF software, the resultant flow cytometry file (.fcs) was normalized against the EQ bead signals and randomized for a uniform negative distribution. FlowJo software was used to deconvolute live, lin(CD3,19,20,56)$^{-}$HLA-DR^{+} PB or BM cells by manual gating. For individual PB and BM DC and monocyte phenotyping analyses (Figure 1G), random sampling without replacement was performed to select up to 2,300 CD141^{+}Clec9A^{+} cDC1, 8,000 CD123^{+}CD303^{+} pDC, 10,000 CD2^{+}FCER1A^{+} CD1c^{+}DC, 10,000 CD88^{+}CD14^{+} monocytes and 4,000 CD88^{+}CD16^{+} monocytes which were concatenated as a .fcs file and subjected to t-distributed stochastic neighbor embedding (tSNE) dimension reduction with perplexity 15 from 1000 iterations, using CD markers 36, 11b, 123, 14, 5, 1c, 11c, 2, 141, 303, 304, 88, 123 and BTLA, AXL, SIGLEC6, IRF4, IRF8, FCER1A, SIRPA. Heat plots of marker expression (ArcSinh scale, with cofactor of 5) on the reduced dimensions were generated within FlowJo.

For combined PB and BM progenitor, pre-DC, DC and monocyte analysis (Figures 5A–5F), combined lin^{-}HLA-DR^{+} cells were down-sampled to select 75,000 cells consisting of 20,000 CD11b^{+}CD14^{+} monocytes, 4,000 CD11b^{+}CD16^{+} monocytes and 50,000 non-monocyte cells. The concatenated .fcs file was subjected to tSNE dimension reduction with perplexity 30 from 1000 iterations using CD markers 14, 16, 123, 11b, 116, 303, 304, 2, 38, 10, 33, 11c, 90, 141, 34, 88, 117, 1c, 5 and Clec9A, AXL, SIGLEC6,

SIRPA, IRF4, IRF8, FCER1A, and BTLA. tSNE plots and marker expression heat plots were generated in ggplot2 R package using tSNE co-ordinates exported from FlowJo.

Diffusion maps and lineage tracing

cells were down-sampled using random sampling within FlowJo, according to the gating strategy in Figures S5B and S5C, to select a total of 14,000 cells consisting of up to 500 or 1000 cells per progenitor or precursor and mature cell population, respectively: GMP33^{+}(300), GMP33^{-}(200), CD123$^{lo\text{-}int}$GMP(298), CD123^{hi}303/4^{lo}(499), CD2^{+}pDC(490), pDC(490), early pre-DC2(498), pre-DC2(491), CD5^{-}DC2(498), CD5^{+}DC2(800), early pre-DC1(500), pre-DC1(254), cDC1(800), pre-DC3/mono(500), pre-DC3(298), CD14^{-}DC3(498), CD14^{+}DC3(1000), pre-mono(500), mono(999). Further analysis was undertaken in R version 3.6.0. Diffusion map calculation was performed with the destiny tool v 2.14.0 (Angerer et al., 2016) using log2-transformed values for the following CD markers: 14, 16, 123, 11b, 116, 303, 304, 2, 38, 10, 33, 11c, 90, 141, 34, 88, 117, 1c, 5, 15 and Clec9A, AXL, SIGLEC6, SIRPA, IRF4, IRF8, FCER1A, BTLA and FLT3. 3D graphics were produced with the rgl package v 0.100.30.

QUANTIFICATION AND STATISTICAL ANALYSIS

Graphs were plotted and statistical analyses performed with Prism 8 (GraphPad software Inc) or in R v3.3.3. Replicate numbers, p values and statistical tests are detailed in the figure legends.

Immunity, Volume *53*

Supplemental Information

Differential IRF8 Transcription Factor Requirement Defines Two Pathways of Dendritic Cell Development in Humans

Urszula Cytlak, Anastasia Resteu, Sarah Pagan, Kile Green, Paul Milne, Sheetal Maisuria, David McDonald, Gillian Hulme, Andrew Filby, Benjamin Carpenter, Rachel Queen, Sophie Hambleton, Rosie Hague, Hana Lango Allen, James E.D. Thaventhiran, Gina Doody, Matthew Collin, and Venetia Bigley

Table S1. DC2 and DC3 marker genes identified by Villani et al., (related to Figure S1)

Transcripts identified as DC2 and DC3 marker genes by Single Cell RNA-Seq in Villani et al., and present in our BM Single Cell RNA-Seq dataset, used to cluster BM DCs in Figure S1J,K

DC2 Genes	DC3 Genes	
SLC2A3	MGST1	IL1RN
FCGR2B	MTMR11	ASGR1
PTGS1	VCAN	NLRP12
CD33	SLC2A3	ADAM15
AREG	RAB27A	S100A8
CLEC4A	FCN1	MPP7
CCR6	LAT2	HNMT
CD2	LYZ	NR4A2
MBOAT7	RETN	PID1
CLEC10A	IL27RA	CD1D
ENTPD1	RAB3D	LMNA
ADAM8	BST1	ITGA5
NR4A2	HBEGF	NLRP3
PID1	CSF3R	S100A9
CLIC2	PLBD1	S100A12
CACNA2D3	CSTA	MNDA
ETS2	NFE2	NOD2
CD1D	F13A1	RNASE2
CD1C	TREM1	PTAFR
CD1E	EREG	CD14
ITGA5	IL1B	FPR1
PEA15	IL13RA1	CD163
NOD2	CLEC10A	FCER1A
PTAFR	MICAL2	TMEM173
PER1	ANXA1	CES1
FCER1A	CD36	NAIP
CLEC17A	AOAH	

Table S2. CyTOF Panel (Related to Figures 1 and 5)

Antigens included in the CyTOF panel and the antibody metal conjugate. For antibody details see **Key Resources Table**

Antigen	Metal	Surface/intracellular
CD14	113In	S
CD370/CLECL9A	141Pr	S
AXL	142Md	S
CD123	143Nd	S
CD11b	144Nd	S
CD116	145Nd	S
SIGLEC6	146Nd	S
CD303	147Sm	S
SIRPA	148Nd	S
IRF8	149Sm	IC
FCER1	150Nd	S
CD2	141Eu	S
IRF4	152Sm	IC
CD45RA	153Eu	S
CD38	154Sm	S
CD36	155Gd	S
CD10	156Gd	S
Lineage-FITC (CD3, 19, 20, 56)	157Gd (secondary)	S
CD33	158Gd	S
CD11c	159Tb	S
ID2	160Gd	S
CD90	161Dy	S
SLAN-PE	162Dy (secondary)	S
BTLA	163Dy	S
CD15	164Dy	S
CD141	165Ho	S
CD34	166Er	S
CD115	167Er	S
CD100	168Er	S
CD304	169Tm	S
CD135	170Er	S
CD88	171Yb	S
CD117	172Yb	S
HLA=DR	173Yb	S
CD1c	174Yb	S
CD5	175Lu	S
CX3CR1-APC	176Yb	S
CD16	209Bi	S
CD45 (PBMC)	115In	S
CD45 (BM)	89Y	S
Cisplatin	195Pt	
DNA	191Ir	

Table S3. Single Cell analysis parameters (related to Figures 1, 3, 4, S3, S4)

**Dataset**		**BM CD34+ Progenitors**	**BM CD34med pre-DC and DC**	**PB pre-DC**
**Figures**		Figures 3E-J; S3D-H	1H, S1J-L, S4E-K, S4H-J	S4K-Q
**Raw Data Analysis and QC CELLS**	Total cells sequenced	399	260	184
	ERCC % threshold (adjusted for concentration of ERCC spike added)	30%	70%	25%
	Mitochondrial % threshold	25%	15%	30%
	Total features threshold	>2500	>1700	>3000, <5000
	Total counts threshold	>50000	>50000	>25000
	Number of cells retained after cell filtering	262	244	116
**Raw Data Analysis and QC GENES**	Number of genes expressed at > 2 counts in > 2 cells	18791	17850	14458
	Number of genes retained after removing cell cycle genes	18181	17237	13920
	Number of protein-coding genes retained	12406	12137	10346
**Whole dataset (all cells, all genes)**	**Hierarchical Clustering**	**Figure 3F, S3H**	**Figure F, S4J**	**Figure S4N**
	Cells in analysis	262	244	116
	Genes retained for analysis by SC3	6,846	6,838	7,265
	Clustering solutions explored	2:10	2:15	2:15
	Clustering solution	10	15	8
	Average Silhouette width	0.45	0.58	0.27
	Highest cluster stability index	0.63	0.7	0.4
	Marker gene p value threshold	0.01	0.01	0.05
	AUROC threshold	0.75	0.85	0.7
	**tSNE**	**Figure 3E-G**	**Figure 4E-G, S4I**	**Figure S4O-P**
	Cells in analysis	262	244	116
	Genes in analysis	6,846	6,838	7,265
	PC for tSNE	10	20	5
	Total variance explained by PCs selected for tSNE	25	35	26
	Perplexity setting for tSNE	13	15	20
	**Diffusion Map**	**Figure 3H-I**	**Figure 4H-I**	
	Number of PC selected for diffusion map	20	10	
**Partial dataset (cell subsets)**	**Hierarchical Clustering**	**Figure 3D (GMP only)**	**Figure 1H (mature DC only, all genes)**	
	Cells in analysis	58	88	
	Genes retained for analysis by SC3	6,846	6,838	
	Clustering solutions explored	2:15	2:15	
	Clustering solution	4	8	
	Average Silhouette width	0.5	0.67	

	Highest cluster stability index	0.3	0.8	
	Marker gene p value threshold	0.1	0.01	
	AUROC threshold	0.75	0.85	
	**tSNE**	**Figure S3E-G**		
	Cells in analysis	58		
	Genes in analysis	6,846		
	PC for tSNE	5		
	Total variance explained by PCs selected for tSNE	30		
	Perplexity setting for tSNE	13		
**Partial dataset (cell and gene subsets)**	**Hierarchical Clustering**		**Figure S1K (mature DC only, DC2/3 genes)**	
	Cells in analysis		88	
	Genes retained for analysis by SC3		61 out of 71 genes found in Villani et al.	
	Clustering solutions explored		2:15	
	Clustering solution		7	
	Average Silhouette width		0.53	
	Highest cluster stability index		0.75	
	Marker gene p value threshold		0.05	
	AUROC threshold		0.75	
	**tSNE**		**Figure S1L (71 DC2/3 genes)**	
	Cells in analysis		88	
	Genes in analysis		71	
	PC for tSNE		35	
	Perplexity setting for tSNE		15	

Table S4. Cell input/output and donor details for *in vitro* DC Culture experiments (related to figures 3, 4, S3, S4).

Figure	Input subset	Donor	Input Cell No	Output Cell No				
				Mono	CD14+ DC3	CD14- DC2	cDC1	pDC
CD34^{+} Progenitor analysis 3C, S3B	Bulk CD34+	1	1000	243	393	7515	1690	408
		1	3258	529	1614	5617	217	12
		1	3258	554	1081	2037	275	9
		2	496	10	343	1332	44	24
		3	500	868	3520	1250	355	50
		3	3090	53	262	742	180	120
		3	3090	27	265	844	145	33
		3	3090	400	374	288	145	65
		4	2600	208	1588	8185	347	179
		4	1642	0	22	1256	125	138
		5	792	6	295	4664	712	106
		6	800	94	328	1583	91	23
		7	6200	355	2250	2556	195	57
		8	3135	45	838	2783	624	50
		8	3135	53	367	1014	279	8
		8	3248	56	61	424	67	14
		9	3056	46	1477	4577	315	51
		10	1712	6	625	706	11	7
		10	2911	29	1574	2650	17	66
		10	2911	802	4996	1925	43	37
		11	848	0	10	120	10	6
		12	1343	5250	5734	1043	0	0
		13	446	22	1854	343	0	0
	CMP	1	3362	122	301	95	0	0
		1	1426	31	272	33	0	0
		2	500	12	0	0	0	0
		4	1558	0	0	1489	0	2
		5	2409	0	210	120	0	0
		14	3040	0	1	0	0	0
		14	1646	51	99	11	1	0
	GMP33+	1	2000	643	4369	11989	1511	51
		2	507	324	1570	397	2	0
		3	500	1538	4467	4346	633	226
		4	1168	142	1572	12966	24	20
		5	1670	144	1169	2211	271	52
		6	669	858	99	39	0	0
		6	70	140	20	0	0	0
	LMPP	1	1500	126	605	3546	3589	827
		1	2957	150	223	1525	1277	757
		2	502	238	618	6479	714	413
		3	428	99	298	7017	4556	3800
		4	215	0	6	9628	358	778
		5	1729	2	21	7262	793	993
		14	1708	7	105	2439	169	86

	GMP33-	1	3042	268	750	456	451	178
		3	558	37	511	3541	2649	480
		4	364	0	246	1148	495	59
		5	848	22	194	2998	437	77
		10	502	0	27	30	36	23
		14	1709	4	64	163	205	34
	GMP123low	3	550	9	278	1502	119	222
		4	419	0	10	192	23	52
		5	491	1	19	363	145	40
	GMP123med	1	700	0	35	271	709	234
		1	2462	0	54	37	466	159
		3	445	0	52	65	394	39
		4	252	0	9	191	45	134
		5	152	0	34	138	190	37
		10	953	0	6	10	20	2
		14	497	0	0	3	4	4
		15	1913	26	75		312	158
**CD34^{med} Precursor analysis 4B, S4C**	123high 303/4low	1	2182	0	12	202	75	191
		1	2182	0	5	150	73	35
		8	1622	0	0	7	40	30
		8	1622	0	1	23	23	2
		16	1521	0	0	2	5	50
		17	1126	0	2	11	1	57
	CD2+ pre-pDC	15	1229	0	0	2	10	181
		16	1005	0	1	2	4	84
		16	1005	0	1	6	1	103
		17	536	0	0	1	0	55
		17	536	0	0	0	0	49
	CD123high CD5+ early pre-DC2	1	434	0	0	10	0	0
		5	444	0	14	65	4	1
		8	469	0	5	41	0	0
		8	1194	0	4	97	1	3
	CD123med CD5+ pre-DC2	3	548	1	113	108	0	3
		4	717	0	0	11	2	1
		8	679	0	2	34	0	0
		8	483	0	0	4	0	0
	CD34med CD123med SIRPA-	1	683	0	16	64	159	0
		9	1596	0	36	54	156	8
		18	630	0	0	16	29	3
		19	1701	0	13	54	144	194
	CD34med SIRPA+	3	3818	16	109	39	18	0
		8	3040	15	27	0	0	0
		10	2703	59	295	29	0	0
		15	1802	0	24	30	0	0
		20	869	18	2557	118	8	0
	SIRPA+CD2+ pre-DC3	3	4045	2	6	1	0	0
		4	329	0	21	17	0	0
		5	2449	18	37	13	0	0
		10	2724	6	6	1	0	0
	SIRPA+CD2- pre-mono	3	4942	14	5	0	0	0
		10	3176	4	3	1	0	0
		15	4966	1	0	0	0	0
		20	1419	6	810	53	1	0

Figure S1, related to Figure 1

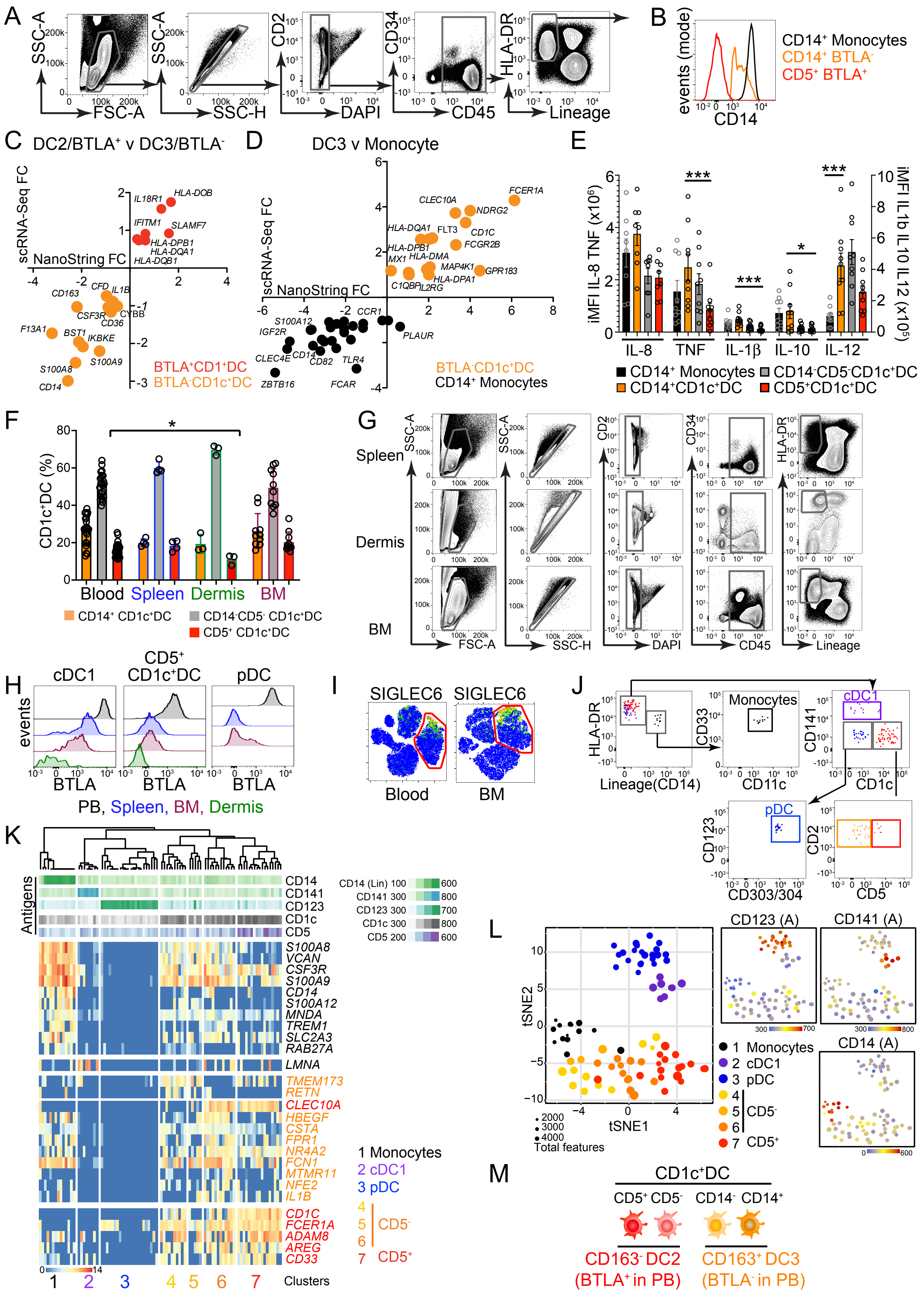

Figure S1: CD1c^{+}DC heterogeneity is evident in human bone marrow
A. Upstream gating steps for the flow cytometric analysis of HC PB monocyte and DC subsets identified PBMC by light scatter properties, excluded doublets, dead and CD45^{-} cells before lin(CD3, CD16, CD19, CD20, CD56)$^{-}$HLA-DR^{+} cells were selected for further analysis in **Figure 1A**. Representative example of n=22

B. Histogram of CD14 expression on CD5^{+}BTLA^{+}CD1c^{+}DCs (red), CD14^{+}CD1c^{+}DCs (orange) and CD14^{+}CD88^{+} monocytes (black), by flow cytometric analysis

C. Correlation of expression fold change of differentially expressed genes by NanoString analysis with the fold change of differentially expressed genes in single cell RNA-Seq analysis described by Villani et al., comparing BTLA^{+}CD1c^{+}DCs (red) (mean of n=3) and BTLA^{-}CD1c^{+}DCs (orange) (mean of n=3) with DC2 and DC3 described by Villani et al. 2017.

D. Correlation of expression fold change of differentially expressed genes by NanoString analysis with the fold change of differentially expressed genes in single cell RNA-Seq analysis described by Villani et al., comparing BTLA^{-}CD1c^{+}DCs(orange) (mean of n=3) and monocytes (black) (mean of n=3) with DC3 and monocytes described by Villani et al. 2017.

E. Intracellular flow cytometric analysis of *in vitro* cytokine elaboration by PB monocytes (black), CD14^{+}CD1c^{+}DC (orange), CD14^{-}CD5^{-}CD1c^{+}DC (gray) and CD5^{+}CD1c^{+}DC (red) from n=9 healthy donors in response to 14hrs stimulation with TLR agonists (CpG, poly(I:C), CL075, LPS). Integrated median fluorescence intensity (iMFI) was calculated by multiplying the frequency of positive cells by the MFI of a given marker. P values were derived from paired two-tailed t-tests (* $p<0.05$; ** $p<0.01$; *** $p<0.005$). Bars show mean±SEM and circles represent individual donors.

F. Relative proportions of CD5^{+}CD1c^{+}DCs (red), CD14^{+}CD1c^{+}DCs (orange) and CD14^{-}CD5^{-}CD1c^{+}DCs (gray) in HC PB (n=22) and BM (n=13) expressed as a percentage of the total CD1c^{+}DC (gated as shown in **Figure 1A**). * $p=0.046$ (Mann Whitney U, two-tailed). Bars show mean±SD and circles represent individual donors.

G. Upstream gating steps for the flow cytometric analysis of HC spleen, skin (dermis) and BM monocyte and DC subsets identified mononuclear cells by light scatter properties, excluded doublets, dead and CD45^{-} cells before lin(CD3, CD16, CD19, CD20, CD56)$^{-}$HLA-DR^{+} cells were selected for further analysis in **Figure 1E** (representative example of n=3 for each tissue) and **F** (representative example of n=13)

H. Histograms of BTLA expression on cDC1, CD5^{+}CD1c^{+}DC and pDC from PB, spleen, BM and dermis (pDC absent), by flow cytometric analysis.

I. tSNE visualization of the expression of TFs IRF4 and IRF8 and surface markers across HC PB and BM lin(CD3,19,20,56,161)$^{-}$HLA-DR^{+} cells by simultaneous CyTOF analysis as in **Figure 1G**. Heat map shows SIGLEC6 expression.

J. Flow cytometric identification of index sorted human BM DC and monocytes for single cell transcriptomic analysis in **Figure 1H** and **S1K,L**.

K. Hierarchical clustering of single cell transcriptomes of mature DC from BM using 71 DC2 and DC3 marker genes identified in Villani et al. to define 7 clusters used to annotate **Figure S1L** (in SC3 software $p<0.05$, AUROC>0.75). Heatmap shows log2 expression. Genes enriched in DC2 or DC3 are shown in red and orange type, respectively. The top rows display fluorescence intensity of surface antigens ('Antigens') from index sorted cells.

L. tSNE plots of the first 35 principal components of the DC2 and DC3 single cell transcriptome dataset (as described in **J,K**) of index sorted pDC, cDC1, CD1c^{+}DC and monocytes. The large panel depicts cells annotated by 7 clusters generated in **K**. Smaller panels show the expression of key subset-specific antigens taken from indexed flow cytometry.

M. Schematic of the phenotypic definition of DC2 and DC3 in PB and tissues; CD163^{-} CD5$^{+/-}$(BTLA^{+} in PB) DC2 and CD163^{+}CD14$^{+/-}$(BTLA^{-} in PB) DC3.

Figure S2, related to Figure 2

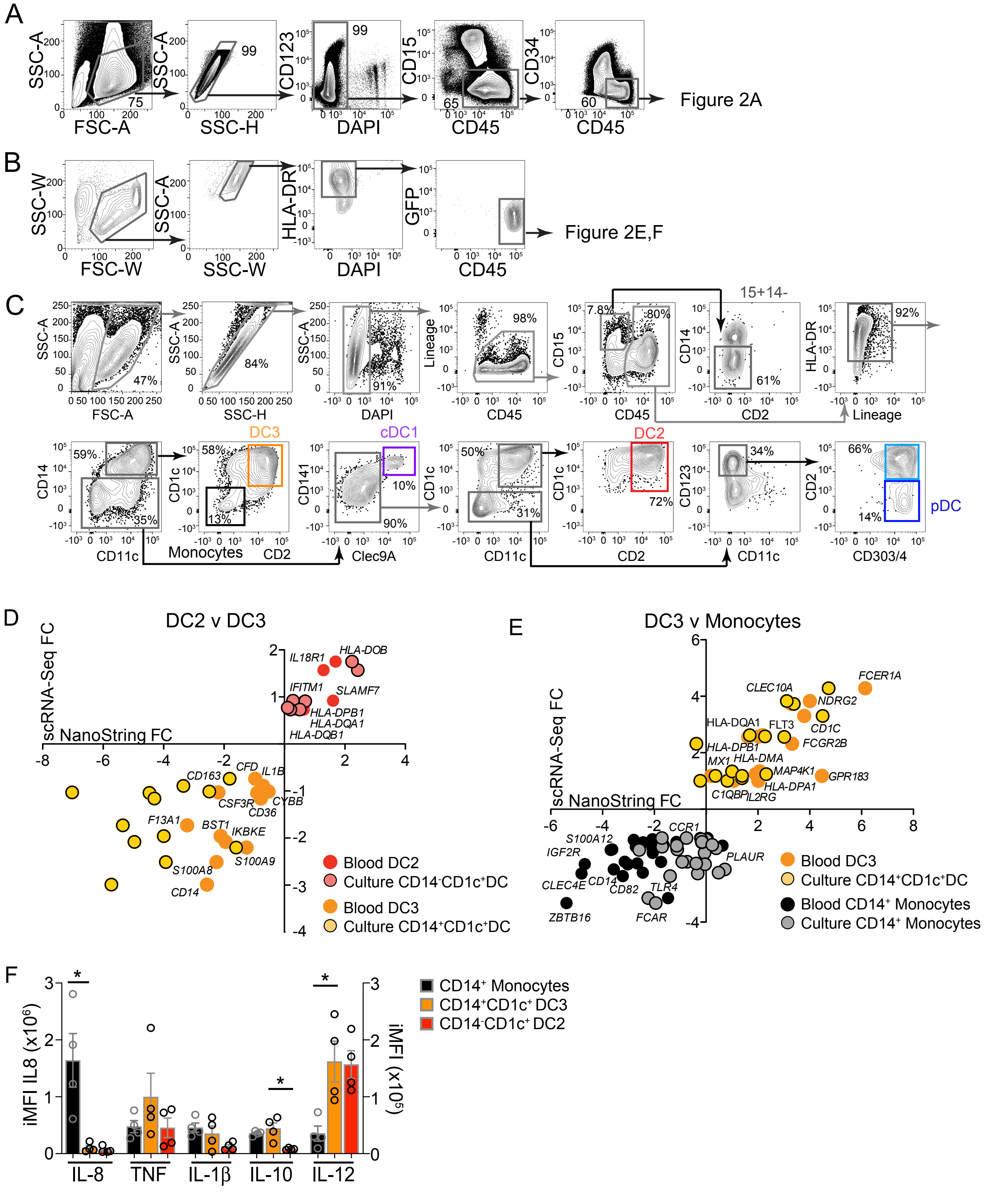

Figure S2: CD14 expression distinguishes between CD1c^{+}DC subsets generated *in vitro*

A. Upstream gating steps for the flow cytometric analysis of *in vitro* derived DC and monocyte subsets. Gates identified live, singlet, cells and excluded CD15^{+} neutrophils and CD34^{+}progenitors before DC and monocyte identification as shown in **Figure 2A**.

B. Upstream gating steps for the analysis of FACS-purified CD1c^{+}DC subsets after 7 days of culture (**Figure 2E,F**). A representative example of the upstream gating from the culture output of CD14^{-}BTLA^{-}DC3 (n=7).

C. Full gating strategy for FACS-purification of *in vitro* generated DC and monocyte subsets derived from BM CD34^{+} progenitors after 21days in culture, for NanoString gene expression analysis (**Figure 2G,H**) and TLR elaboration (**Figure 2I**).

D, E. Correlation of expression fold change of differentially expressed genes by NanoString analysis with the fold change of differentially expressed genes in single cell RNA-Seq described by Villani et al. (in a similar analysis to **Figure S1**), comparing D) DC2 (red) and DC3 (orange) or E) DC3 and monocytes (black). PB DC (BTLA^{+}CD1c^{+}DC2 and BTLA^{-}CD1c^{+}DC3) and their culture-derived counterparts (CD14^{-}CD1c^{+}DC2 and CD14^{+}CD1c^{+}DC3, outlined dots) are shown. Fold change derived from mean expression of n=3 for PB and n=3 for culture-derived cells.

F. Intracellular flow cytometric analysis of *in vitro* cytokine elaboration by CD14^{+}1c^{-} monocytes (black), CD14^{+}CD1c^{+}DC3 (orange) and CD14^{-}CD1c^{+}DC2 (red) generated from n=4 BM donors after 21 days in culture, in response to TLR agonists (CpG, poly(I:C), CL075, LPS). Integrated median fluorescence intensity (iMFI) was calculated by multiplying the frequency of positive cells by the MFI of a given marker. P values were derived from paired two-tailed t-tests (* $p<0.05$). Bars show mean±SEM and circles represent individual donors.

Figure S3, related to Figure 3

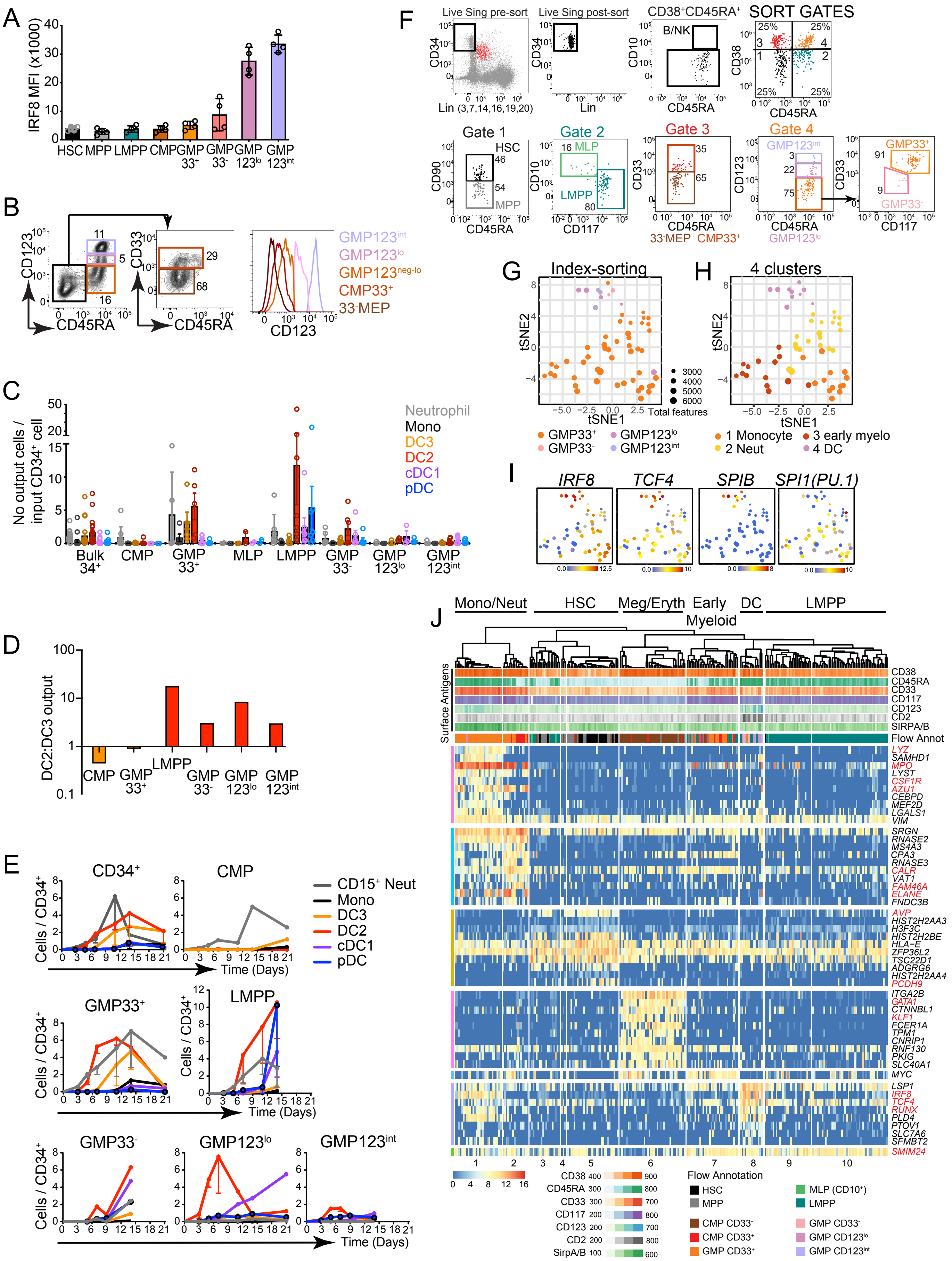

Figure S3. High IRF8 expression defines LMPP-associated DC progenitors

A. Median Fluorescence intensity of Intracellular IRF8 by flow analysis across gates identifying CD34+progenitor populations of HC BM (n=4). HSC, hematopoietic stem cell; MPP, multipotent progenitor; MEP, megakaryocyte erythroid progenitor; MLP, multilymphoid progenitor; LMPP, lymphoid primed multipotent progenitor; CMP, common myeloid progenitor; GMP, granulocyte macrophage progenitor. Bars represent mean±SD.

B. Flow analysis of $CD34^{+}CD38^{+}CD10^{-}$ progenitor populations (as shown in **Figure 3A**) showing the relative levels of CD123 expression across $CD33^{+}CD123^{neg\text{-}lo}$ (predicted CMP) and $CD33^{-}CD123^{-}$ (predicted MEP) $CD45RA^{-}$ populations and $CD45RA^{+}$GMP fractions.

C. Absolute proliferative capacity and differentiation potential of progenitor fractions subjected to 14 days in DC differentiation culture, as depicted in **Figure 3C**, expressed as the number of cells generated per input $CD34^{+}$ cell. $CD15^{+}$ neutrophils are included. Bars show mean±SEM, circles represent individual donors.

D. The relative output (ratio) of DC2 and DC3 from the specified $CD34^{+}$progenitor fractions. Orange bars DC2<DC3; red bars DC2>DC3.

E. Kinetics of DC, monocyte and neutrophil output from progenitor fractions over 14-21 days in DC differentiation culture. $CD34^{+}$ n=3 (n=2-3/timepoint), CMP n=1, $GMP33^{+}$ n=3 (n=1-3/timepoint), LMPP n=3 (n=2-3/timepoint), $GMP33^{-}$ n=3 (n=1-2/timepoint), $GMP123^{lo}$ n=2 (n=1-2/timepoint), $GMP123^{int}$ n=3 (n=1-3/timepoint). Dots and bars represent mean–SEM.

F. Flow identification of index sorted human BM progenitors for single cell transcriptomic analysis in **Figures 3D-J and S3G-J.** A tight lin(CD3, 7, 14, 16, 19, $20)^{-}$ gate was used to select $CD34^{+}$ progenitors for single cell sorting. This excluded $lineage^{lo}$ $CD10^{+}$B/NK progenitors, as defined in **Figure 3A**, shown backgated (red dots) onto the pre-sort BM live, singlet, mononuclear cells (gray). Top middle panel shows the same $Lin^{-}34^{+}$ gate on sorted single cells. Top right panel demonstrates the absence of $CD10^{+}$B/NK cells in the sorted $CD38^{+}CD45RA^{+}$ population.

G-I. tSNE visualization of the first 5 principal components (30% total variance) of the transcriptomes of 58 single GMP, analyzed independently of surface phenotype. tSNE plots are annotated by **G**, gate of origin from index-linked flow (**Figure S3F**) or **H**, 4 hierarchical clusters (**Figure 3D**) showing clustering of cells from the $CD33^{-}$, $CD123^{lo}$, and $CD123^{int}$ gates (cluster 4) away from clusters 1-3 containing $GMP33^{+}$ cells. Heatmaps (**I**) show log2 expression of key DC TF genes on tSNE plot in **G** and **H**.

J. Unsupervised hierarchical clustering of single cell transcriptomes of all progenitor cells, using all protein-coding, non-cell cycle genes (independent of surface antigen expression). Marker genes for 10 clusters generated within SC3 (p<0.01, AUROC>0.75) are displayed, examples of which identify cluster 1, monocyte (*LYZ*, *CSF1R*); cluster 2, granulocyte (*CALR*, *ELANE*), clusters 3-5, HSC and MPP (*AVP*, *PCDH9*), cluster 6, MEP (*GATA1*, *KLF1*), cluster 8, DC (*IRF8*, *TCF4*, *RUNX*), clusters 9-10, LMPP (*SMIM24*). Heatmap shows log2 gene expression. The top rows display fluorescence intensity of surface antigens (‘Surface Antigens’) from index sorted cells, ‘Flow annotation (Flow annot)’ denotes the classification of index sorted cells by their surface phenotype (**Figure S3F**).

Figure S4, related to Figure 4

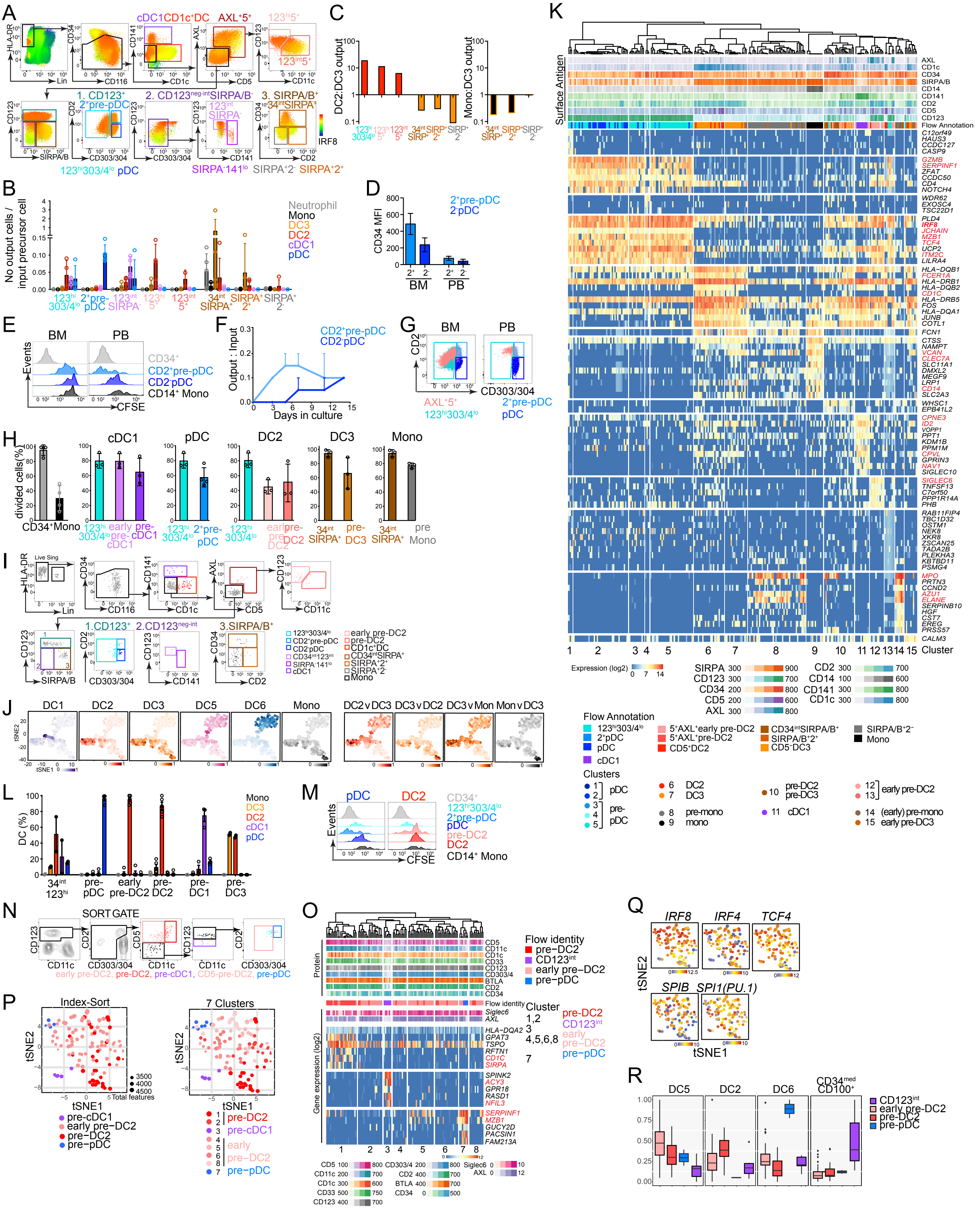

Figure S4. Two trajectories of DC development connect the progenitor compartment with mature DC

A. Heatmaps showing IRF8 expression by intracellular flow cytometric analysis across sequential bivariate plots, using the gating strategy to identify DC and pre-DC shown in **Figure 4A**. Heatmaps generated in FlowJo 10.6.1.

B. Absolute proliferative capacity and differentiation potential of CD34^{int} precursor fractions subjected to 14 days in DC differentiation culture, as depicted in **Figure 4C**, expressed as the number of cells generated per input precursor cell. CD15^{+} neutrophils are included. Bars show mean±SEM, circles represent individual donors.

C. The relative output (ratio) of DC2 versus DC3 (left graph) or Monocyte versus DC3 (right graph) from the specified CD34^{int} precursor fractions. Red bars DC2>DC3; orange bars DC3>DC2, black bars with orange outline DC3>monocyte.

D. Mean fluorescence intensity (MFI) of CD34 by flow cytometric analysis of CD2^{+} and CD2^{-} pDC from BMMC (n=3) and PBMC (n=3). Plots show mean±SEM.

E. CFSE dilution in CD2^{+} and CD2^{-} pDC from BM and PB after 3 days in DC culture, to assess proliferation. Histograms show CD34^{+} progenitors and monocytes as controls. Plots shown are representative of n=3 experiments in PB and BM.

F. Kinetics of the output generated from 14 days culture of CD2^{+} pDC, expressed as the number of cells generated per input cell. n=2 donors with n=2 analyses at each time point. Dots and bars show mean+SEM.

G. Flow cytometric analysis of Lin^{-}HLA-DR^{+}CD1c^{-}CD141^{-}CD123^{hi} PBMC and BMMC showing the location of AXL^{+}CD5^{+}CD123^{+}CD11c^{-} early pre-DC2 (pink) on the bivariate plot of CD2 versus CD303/4, overlying the CD34^{int} CD123^{hi}CD303/4^{lo} population (turquoise).

H. Summary of the proliferative capacity of BM DC precursors. BMMCs were CFSE stained then precursors FACS-purified and cultured in DC-culture conditions for 3 days before flow cytometric analysis of CFSE. Bulk CD34^{+} progenitors and CD14^{+} monocytes were included as positive and negative controls, respectively. Bars represent mean±SD of n=3-4 donors, circles represent individual donors.

I. Location, in flow cytometry parameter space, of index-sorted precursor cells purified for scRNA-Seq depicted in **Figure 4E-J, S4J-K)**.

J. DC subset signature scores generated from Villani et al, mapped across the tSNE plot of precursor and mature DC single cell transcriptomes (**Figure 4E-J**). To generate signature scores, the dataset was mined for expression of cell subset ‘signature’ genes defined by single cell RNA-Seq in Villani et al., including DC1 (cDC1), DC2 (cDC2a), DC3 (cDC2b), DC5 (AXL^{+}), DC6 (pDC) and monocytes. Normalized counts were then rescaled from 0 to 1 and the average scaled score displayed on t-SNE plots generated as described in the STAR methods. The left panel shows the level of expression of DC subset signature scores. The right panel shows the mapping across tSNE space of similar scores generated from differentially expressed genes after pairwise comparisons of subsets, as indicated.

K. Unsupervised hierarchical clustering of single cell transcriptomes of BM precursor and mature DC subsets and $CD14^+$ monocytes, using all protein-coding, non-cell cycle genes (independent of surface antigen expression). Marker genes for 15 clusters generated within SC3 ($p<0.01$, AUROC>0.85) are shown. Examples used to identify clusters are marked in red and derived from subset signature genes in Villani et al.. Clusters were used to annotate **Figure 4F, H**. The top rows display fluorescence intensity of surface antigens ('Surface Antigens') from index sorted cells. 'Flow Annotation' denotes the classification of index sorted cells by their surface phenotype (in **Figure S4I**), Cluster color code related to **Figure 4F,H**.

L. The output of *in vitro* culture of DC precursors from PB, FACS-purified using the gating strategy shown in **Figure 4C** showing lineage-specific enrichment from precursors, analogous to the output from phenotypically similar BM precursors. Output is expressed as the proportion of each DC and monocyte population as a percentage of the total cells captured by all DC and monocyte gates. n=3-5 donors for each population. Bars represent mean+SEM, circles represent individual experiments.

M. Proliferative potential of PB DC precursors at day 3 of culture, estimated by CFSE dilution. Bulk $CD34^+$ progenitors and $CD14^+$monocytes were included as positive and negative controls, respectively. The CFSE dilution histograms for each precursor are grouped and ordered according to their proposed position in the developmental trajectory for pDC or DC2 lineages. Results are representative of n=3 experiments.

N. Sorting strategy applied to $Lin^-HLA\text{-}DR^+$ $CD1c^-CD141^-$ cells, and location, in flow cytometry parameter space, of subsequently index-sorted PB precursor cells ($CD2^-$ pDC excluded) purified for scRNA-Seq analysis depicted in **Figure S4O-R**.

O. Hierarchical clustering of single cell transcriptomes of PB pre-DC populations showing signature genes that identify 8 clusters ($p<0.05$ and AUROC>0.7), used to annotate **Figure S4P**. These defined $CD11c^+$ pre-DC2 (cluster 1 and 2; marked by *CD1c* and *SIRPA* at RNA level), $AXL^+CD5^{+/-}CD11c^{lo}$ early pre-DC2 (clusters 4,5,6,8), $CD34^{int}CD123^{int}AXL^-$ cells, marked by *NFIL3* (critical for cDC1 development, Bagadia et al., 2019) and shown to have enriched cDC1 potential in culture (**Figure S4L**) and a small number of $AXL^-CD5^-CD303/4^{hi}$ pre-pDC (contained within cluster 7, marked by *SERPINF1*, *MZB1*).

P. tSNE of the first 5 principal components (26% total variance) of 116 single cell transcriptomes sampled from PB pre-DC populations, annotated by the gate of origin from index-linked flow cytometry (Index Sort) or by 7 clusters generated from hierarchical clustering **Figure S4O**. Pre-DC2 and pre-pDC were polarized in tSNE space, linked by early pre-DC2 which showed variable expression of both pDC and $CD1c^+$DC genes.

Q. Heatmaps show expression of key TF on the tSNE plot from **Figure S4P**.

R. Mapping of single cell transcriptomes to cell populations identified by Villani et al. Cells identified as early pre-DC2 and pre-DC2($CD123^+CD5^+CD303/4^{lo}$) showed enrichment of gene expression with 'AS' DC (DC5) and DC2; pre-pDC ($CD123^+CD2^+CD303/4^{hi}$) mapped to pDC (DC6) and the $CD34^{int}CD123^{int}SIRPA^-$ population (early pre-cDC1) showed enrichment of genes expressed by a $CD34^{int}CD100^+$ population also found in Villani et al. to contain cDC1 potential *in vitro*.

Figure S5, related to Figure 5

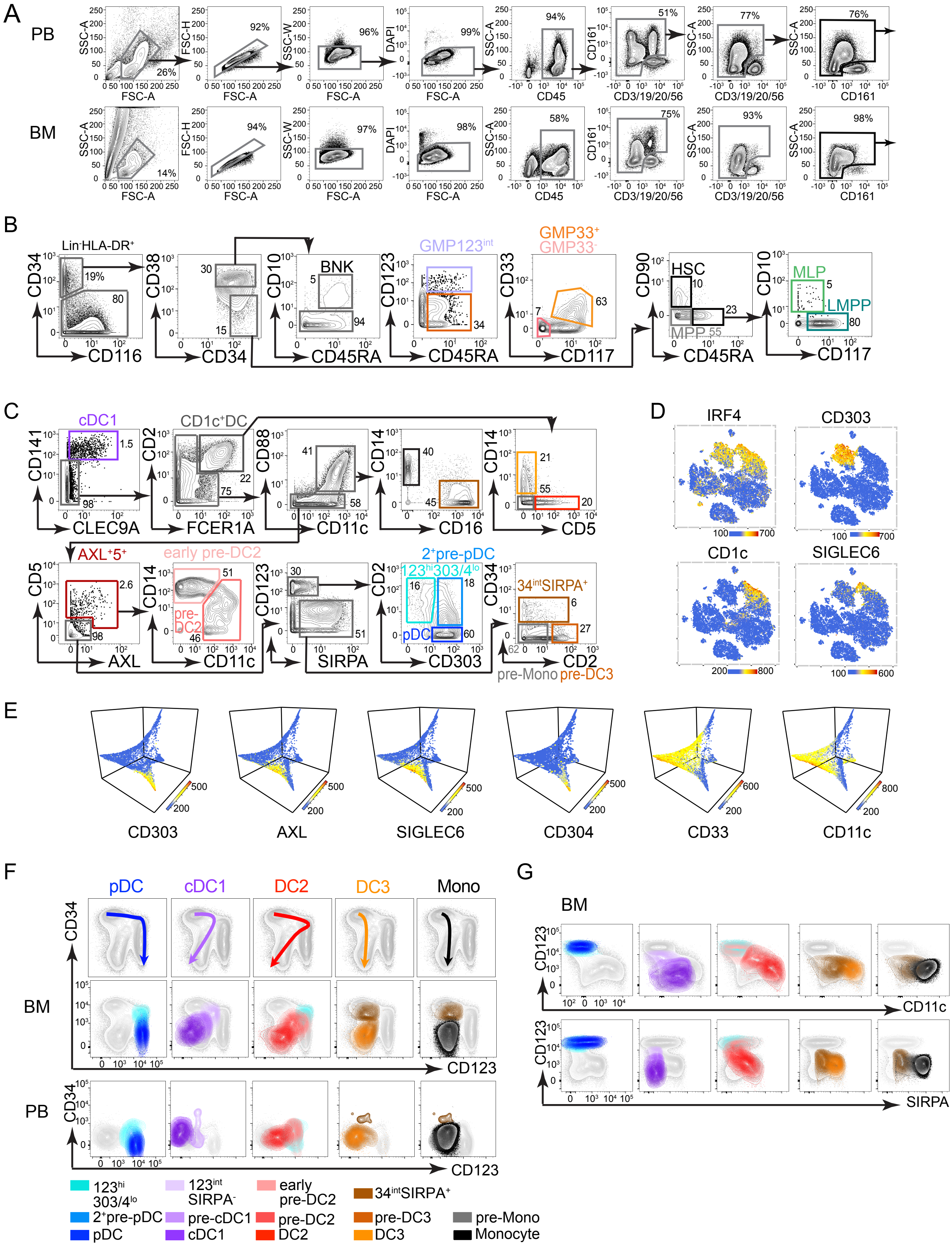

Figure S5. Differential IRF8 expression defines the two trajectories of DC development

A. Flow cytometric gating strategy for the FACS-purification of PBMC and BMMC to enrich for $CD45^{+}$ cells and exclude lin(CD3,19,20,56,161)$^{+}$ lymphocytes prior to CyTOF analysis (**Figure 5A-E**).

B. Gating of bivariate plots from CyTOF analysis to identify $CD34^{+}$ progenitor subsets, comparable with flow cytometric analysis in **Figure 3A**.

C. Gating of bivariate plots from CyTOF analysis to identify DC and monocyte subsets and their precursors, comparable with flow cytometric analysis in **Figure 4A**.

D. Heatmap expression of IRF4 and additional key antigens (CD303, CD1c, SIGLEC6) across the tSNE visualization plot shown in **Figure 5A-E**.

E. Heatmaps of the expression (log2) of additional key antigens superimposed across the diffusion map trajectories generated with 14,000 GMP, precursor and mature DC and monocyte cells and 29 markers to infer pseudo-temporal ordering of cells and reconstruct lineage branching, as shown in **Figure 5F**.

F. Back-gating of $CD34_{neg\text{-}int}$ DC precursors and DC subsets (defined in **Figure 4A**) on bivariate plots, to relate the DC developmental pathways to standard flow analysis. The relative expression of CD34 and CD123 is visualized across populations comprising lineage-specific developmental pathways as defined by the previous data. Schematic arrows summarize the proposed sequence of maturation of gated populations across the 2D space, from $CD34^{+}$ progenitor compartment to mature DC populations. The utility of BM as a source material and relative paucity of DC precursors in PB is also illustrated.

G. Lineage-specific $CD34_{neg\text{-}int}$ DC precursors and DC subsets from BM, as defined in **Figure 4A**, backgated onto bivariate plots of CD123 versus CD11c and CD123 versus SIRPA/B to visualize the relative expression of these antigens on populations comprising lineage-specific developmental pathways.

Figure S6, related to Figure 6

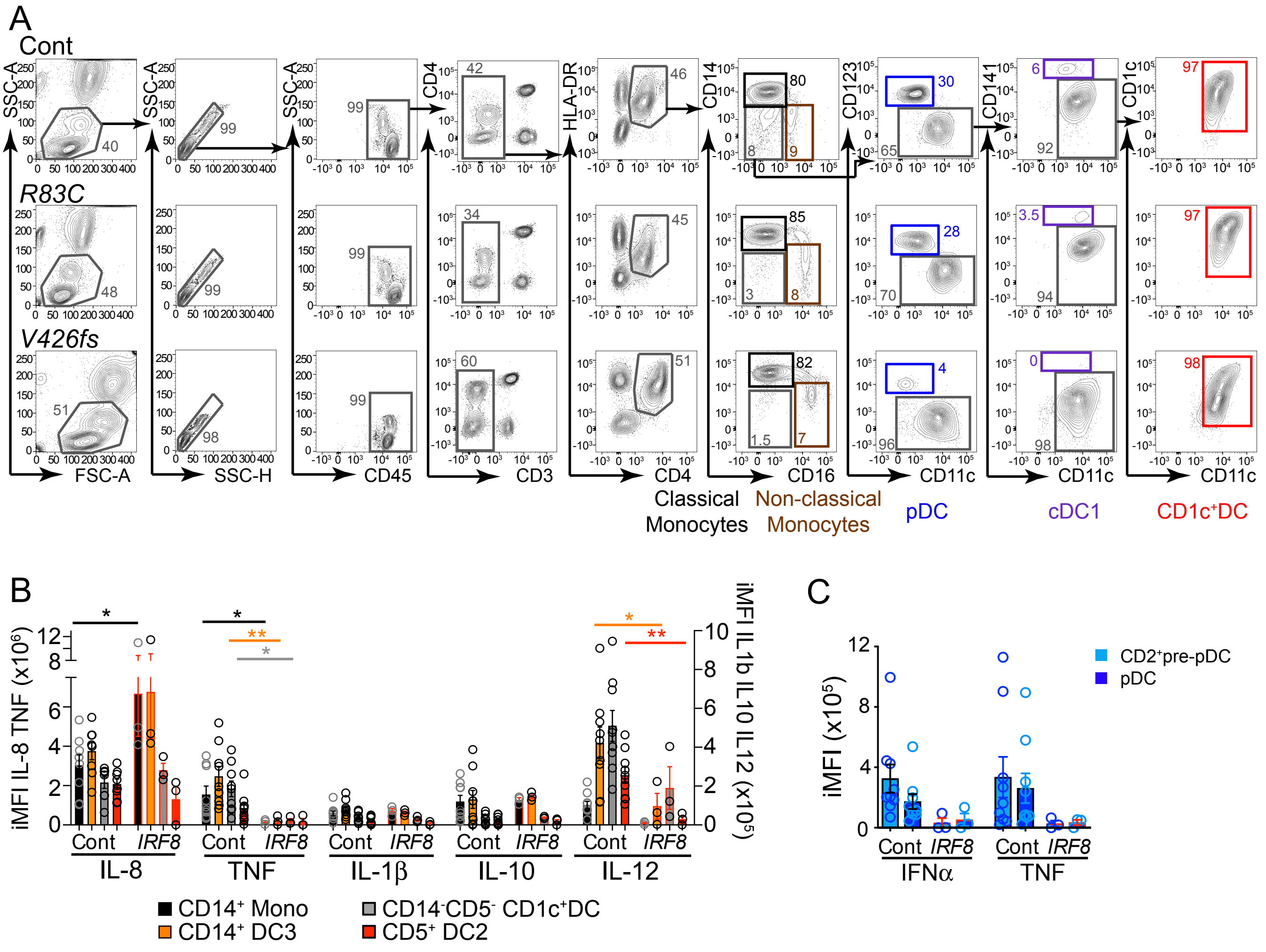

Figure S6. IRF8$_{hi}$ and IRF8$_{lo}$ pathways are differentially compromised in IRF8 deficiency

A. Flow cytometric gating strategy for whole PB Trucount™ analysis of a HC (Cont), subject carrying heterozygous *IRF8* mutation (*R83C*) and subject carrying dominant negative mutation (*V426fs*), summarized in **Figure 6B**. Numbers represent the percent of cells from the parent gate.

B-C. Intracellular flow cytometric analysis of *in vitro* cytokine elaboration by monocytes (black bars), CD14^{+}DC3 (orange), CD14^{-}CD5^{-}CD1c^{+}DC (gray) and CD5^{+}DC2 (red) (B) and CD2^{+}pre-pDC and pDC (C) from HC (n=8) and subjects carrying heterozygous *IRF8* mutations (*R83C*, *R291Q*, mean of technical duplicates; and *V426fs*) in response to 14hrs stimulation with TLR agonists (CpG, poly(I:C), CL075, LPS). Integrated median fluorescence intensity (iMFI) was calculated by multiplying the frequency of positive cells by the MFI of a given marker. Bars show mean±SEM. P values from Mann Whitney U analysis (* $p<0.05$, ** $p<0.01$, *** $p<0.001$).

Figure S7, related to Figure 7

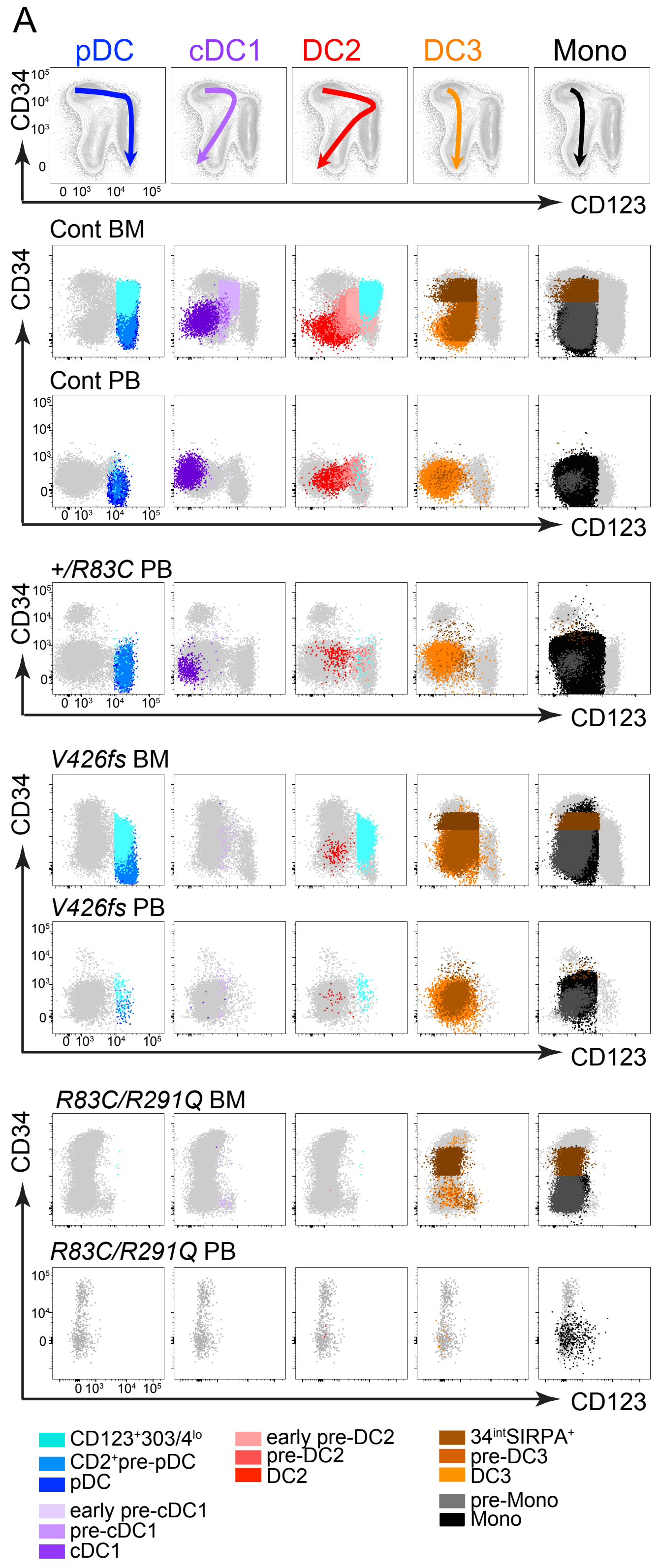

Figure S7. IRF8 deficiency causes dose-dependent blockade of the IRF8hi pathway

A. Summary of DC and monocyte differentiation pathways from BM (where available) and PB of subjects carrying heterozygous *R83C*, dominant negative *V426fs* and bi-allelic *R83C/R291Q IRF8* mutations. DC and precursor populations were gated as shown in **Figure 7B**. These were backgated on bivariate plots to visualize the relative expression of CD34 and CD123 on populations comprising lineage-specific developmental pathways. Arrows indicate the proposed sequence of maturation of gated populations from CD34+ progenitor compartment to mature DC populations.
